# A Novel Hydrogenation of Nitroarene Compounds with Multi Wall Carbon Nanotube Supported Palladium/Copper Nanoparticles (PdCu@MWCNT NPs) in Aqueous Medium

**DOI:** 10.1038/s41598-020-64988-0

**Published:** 2020-05-15

**Authors:** Haydar Göksu, Nursefa Zengin, Hakan Burhan, Kemal Cellat, Fatih Şen

**Affiliations:** 1 Kaynasli Vocational College, Düzce University, Düzce, 81900 Turkey; 2 Sen Research Group, Department of Biochemistry, Dumlupınar University, 43100 Kütahya, Turkey

**Keywords:** Catalysis, Nanoparticles

## Abstract

A novel nanocatalyst, multi-wall carbon nanotube supported palladium/copper (PdCu@MWCNT) nanoparticles, was synthesized for the reduction of nitroarene compounds. Characterization of the nanocatalyst was achieved by XRD, XPS, TEM, and Raman spectroscopy analysis. In this study, the hydrogenation of nitroarenes to primary amine compounds was achieved in aqueous medium at room temperature. The aniline derivatives were synthesized with high yields at mild conditions via novel PdCu@MWCNT nanocatalyst. The conversion of nitroarenes to amine derivatives was accomplished at 99% efficiency. In addition to its high activity, the PdCu@MWCNT catalyst was determined to be stable and reusable after the 3rd consecutive use for the reaction and provided 99% conversion of various compounds in the reduction reaction.

## Introduction

The reduction of nitroarene compounds using a facile and cost-effective method is very important in organic synthesis and industrial applications. The eco-friendliness and reusability should be in the priority for the design of the catalyst and synthesis methods^1,2^. The hydrogenation of organic compounds generally conducted using a suitable precious-metal catalyst such as palladium, iridium, ruthenium, and rhodium^3–6^. Catalytic heterogeneous hydrogenation processes are very important when considering synthetic transformations^7–10^. Catalysis is one of the factors that affect the rate of formation of chemical reactions under mild conditions. The catalysts are preferable since a large number of reactants can be converted with a small amount of the catalyst. In case of more than one product is produced at the end of the reaction, the catalyst may change the ratio of these products and contribute to achieving chemoselectivity which is a very important issue in the chemical industry^11^.

Heterogeneous catalysts have great importance in the production of fine chemicals and organic synthesis^7,12,13^. Metal nanoparticles exhibit superior catalytic and physical properties compared to their bulk form and received considerable attention in the last decades due to their unique structural, catalytic, optical and electronic properties and become preferable in technological applications as nano-electronic devices, sensors, biosensors, biomedical tools, and catalyst^6,14–17^. Pd, which commonly used in organic reactions as a catalyst, is a paramagnetic metal while Pd nanoparticle is ferromagnetic^18,19^. Moreover, Pd nanoparticles have high catalytic activity in hydrogenolysis and hydrogenation reactions^4,6,20^. The catalytic activity of the hydrogenation reactions strongly depended on the size and surface structure of the catalyst^21^. Recently, bimetallic nanoparticles are being utilized in many industrial and scientific applications. The combination of two different species of metals and their fine structures resulted in interesting physicochemical properties which are primarily due to the synergistic effect and demonstrate enhanced catalytic activity compared to monometallic catalysts. The combination of copper and palladium as a catalyst is one of the most popular examples and have been using in different reactions^22–24^.

Several studies have been conducted for catalytic reduction of nitroarene compounds. The main purpose of the catalytic systems is to reduce nitro groups with very high chemoselectivity. A significant progress has been accomplished with the use of noble metals until the recent past, and gold‐based catalysts are the most notable among them^25^. Corma *et al*.^26^ reported an innovative Au/TiO_2_ catalyst that exhibiting high selectivity of 95% for hydrogenation of the nitro group. Although the performance of noble metals is satisfied, high price and limited availability are their main drawbacks. When special supports are used, the percentage of noble metals can be decreased. Thus, the cost of the catalysts can be decreased while maintaining the physical properties^27,28^. Various methods are developed to control the dispersion of the metal on the support and to optimize the catalyst compositions^29,30^. Carbonaceous materials are suitable for use as a support material^5,15^. This is mainly due to their very good electronic properties, high surface area and good stability^31^. In the past decade, some important breakthroughs on non-noble metal catalysts have been reported. Wei *et al*.^32^ developed a catalyst containing cobalt, an earth-abundant non-noble metal, and nitrogen-doped carbon nanotube support for chemoselective catalysis of hydrogenation of nitroarenes. By the doping of nitrogen into the carbonaceous structure, dissociation energies of H_2_ were reduced and the H_2_ activation enabled. As a result of this catalyst design, a wide range of substituted nitroarenes were hydrogenated with very high (>99%) selectivity. In another study, Jagadeesh *et al*.^33^ have been worked on an iron oxide-based catalyst for the chemoselective hydrogenation of nitroarenes to anilines. Similarly, N-doped carbonaceous supported Fe_2_O_3_ catalyst improved the H_2_ activation and allowed selective hydrogenation of nitroarenes under industrially viable conditions. Recently, some metal oxides such as WOx and MoOx are also reported. Song *et al*.^34^ reported the oxygen-deficient tungsten oxide can be used for the dissociation and activation of hydrogen molecules.

Carbon nanotubes (CNTs), one of the various carbonaceous materials, attract attention due to their superior physical properties and used in various fields such as nanobioelectronics^35^, pharmacy^36^, fuel cells^37^, adsorption applications^38^, sensor technologies^39^, etc.

Carbon Nano Tubes (CNTs) have been using as support material in catalysts soon after their first discovery^40^. Their extraordinary properties such as high electrical and thermal conductivity, mechanical strength, low degradation rate, and 1D structure provide new opportunities for catalyst design^40^. Loading the catalytically active metal nanoparticles onto carbonaceous support materials has effectively reduced metal amounts as well as the cost of the catalyst. Moreover, the catalytic activity of precious metals greatly enhanced when supported. The main reason of the increased catalytic activity is due to a better-dispersed metal catalyst, and consequently, better interaction with substrate molecules. However, a functionalization process is necessary to obtain higher performances^41^. Covalent or non-covalent processes are applied to the functionalization of MWCNTs. In covalent functionalization, MWCNT is treated with acid and the closed ends are opened. Carboxyl groups are formed at the opening ends of MWCNT. In the non-covalent process, MWCNT does not deteriorate. The MWCNT with the substance to be bound is kept in the shaker or sonicator. At the end of the process, the desired molecule is coupled to MWCNT^42^.

Herein, we reported a new method for the hydrogenation of various nitroarenes with the PdCu@MWCNT nanocatalyst, synthesized by our group. Characterization of the nanocatalyst was achieved by XRD, XPS, TEM, and Raman spectroscopy analysis. In this study, sodium borohydride and water/methanol mixture were used as hydrogen sources and as a solvent, respectively. The reactions were completed in a short time at room temperature. The results indicated that the as-synthesized catalyst reduces the reaction time and the cost of the system.

## Experimental

### Synthesis of PdCu@MWCNT

The PdCu@MWCNT catalyst was prepared by the ultrasonic reduction method. For this aim, 0.25 mmol of PdCl_2_, 0.25 mmol of Cu_2_O and 100 mg of MWCNT were dispersed in ethanol and kept in an ultrasonic bath for 1 h. Then the resulting mixture was transferred to the Schlenk tube and stirred for one hour. During this stage, N_2_ gas was purged to maintain the inert atmosphere. The reduction process was finalized by the addition of Dimethylamine borane (DMAB).

### General procedure for the PdCu@MWCNT catalyzed hydrogenation of nitroarenes

2 mg of PdCu@MWCNT, 0.25 mmol nitroarene derivatives, and 1 ml of water: methanol mixture (7:3), and sodium borohydride were placed into a reaction vessel and stirred at room temperature. TLC analysis was performed to monitoring the progress of the reaction. After the completion of the reactions, the yields of the products were determined by ^1^H-NMR and ^13^C NMR analysis.

## Results and Discussion

**Figure 1:**
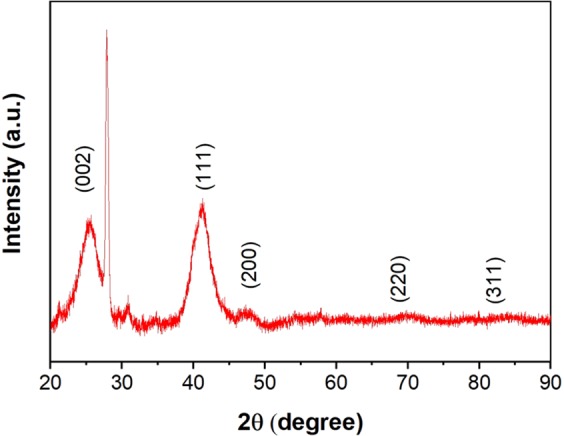
Powder XRD pattern of as-synthesized PdCu@MWCNT catalyst.

The characterization of the PdCu@MWCNT catalyst is achieved by TEM, XRD, and XPS spectroscopy techniques. The XRD pattern of the as-synthesized PdCu@MWCNT catalyst was shown in Fig. 1. It was observed that the XRD pattern consists of well-separated peaks which indicate a face-centered cubic (fcc) crystal lattice structure. The diffraction peaks detected at 2θ degrees of 41.1°, 47.6°, 69.6°, and 83.3° correspond to planes of (111), (200), (220), and (311), respectively. Furthermore, the peak at 2θ degree of 25.6^o^ (002) specified for MWCNT. No peaks were observed corresponded to CuO_2_, this is due to the fact that using a strong reducer was reduced all the CuO_2_ species. However, the forming of PdCu metal alloy shifted XRD pattern peaks to the lower positions. This shift corresponded to the atomic incorporation into the crystal lattice^43^. The average crystallite size was calculated from Pd (111) peak using the Scherrer equation and found to be 4.78 ± 0.43 nm.

**Figure 2:**
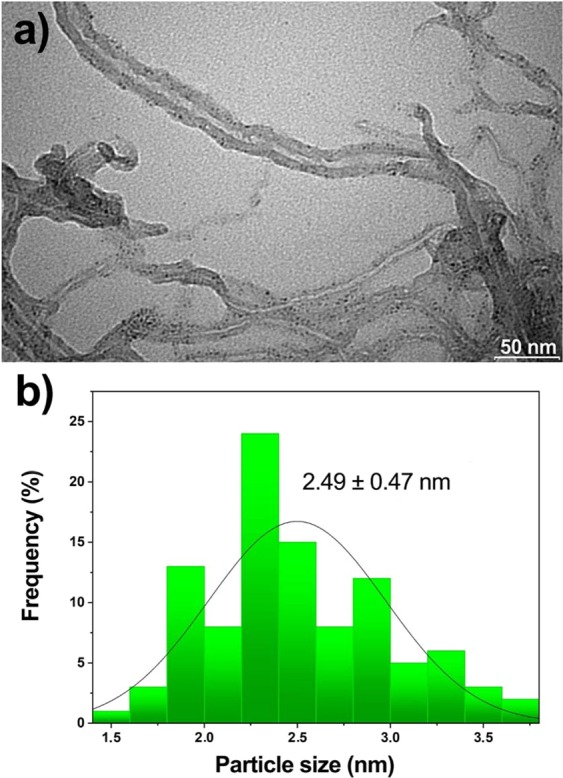
(**a**) TEM images of PdCu@MWCNT, (**b**) PdCu alloy particle size distribution.

Figure 2a shows the TEM image of the PdCu@MWCNT nanocatalyst. TEM image is revealed that there is no agglomeration between the nanoparticles and most of them are formed in spherical. In order to estimate the particle size of the catalyst approximately 300 particles were taken into account and it was found to be 2.49 ± 0.47 nm as shown in particle size histogram in Fig. 2b.

**Figure 3:**
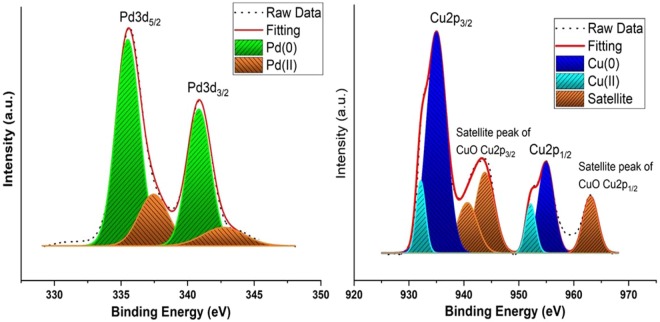
XPS spectra (**a**) Pd 3d region and (**b**) Cu 2p region.

The XPS characterization technique was used to determine the chemical oxidation state and surface composition. The Pd 3d and Cu 2p regions of the spectrum was shown in Fig. 3a,b. The XPS peaks were fitted using Origin Pro 2019b software. Shirley type background correction was applied and the Gaussian-Lorentzian function was used for the peak fitting. The determination of binding energy peaks in the XPS spectrum was evaluated by C 1 s peaks at 284.6 eV. As demonstrated in Fig. 3a, the Pd 3d spectrum of PdCu@MWCNT nanocatalyst, two doublets at the binding energies of 335.6 eV and 341.5 eV corresponded to metallic Pd (0) species. The two doublets of Pd (II) were detected at the binding energy of 337.4 eV and 343.9 eV^44,45^. Peak area comparison indicated that Pd was predominately present in the metallic form. In the XPS spectra of the Cu 2p level region, the Cu 2p_3/2_ and Cu 2p_1/2_ peaks appeared at binding energies of 932.4/934.8 eV and 952.1/954.8 eV, respectively^46^. Additionally, satellite peaks were also observed at 940.3, 943.2 eV and 962.4 eV^47^. When the peak areas of Cu (0) and Cu (II) are compared, it can be seen that Cu (0) is the predominant oxidation state. This is due to the fact that using DMAB, a strong reducing agent, combined with ultrasonic reduction method. Coexistence of low amount oxidized species seen in the XPS spectra are due to the partially surface oxidation of synthesized catalyst.

**Figure 4:**
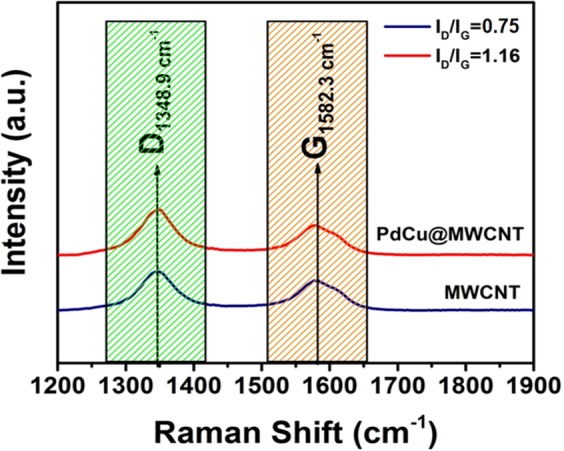
Raman spectra of MWCNT and PdCu@MWCNT.

In the Raman spectrum, the peaks observed at 1348 and 1582 cm^−1^ is related to D band and G band, respectively. The density ratio of the D and G bands shows the defects occurring in the carbon material. An increase in this ratio is associated with an increase in the defect in the MWCNT structure. In Fig. 4, the I_D_/I_G_ ratio increased from 0.75 to 1.16, when PdCu nanoparticles were immobilized to MWCNT. The results suggest the PdCu doping onto the MWCNT structure and free electrons of metal nanoparticles caused a change in sp^2^ atoms which characterized by the change in G band^48^.

**Figure 5:**
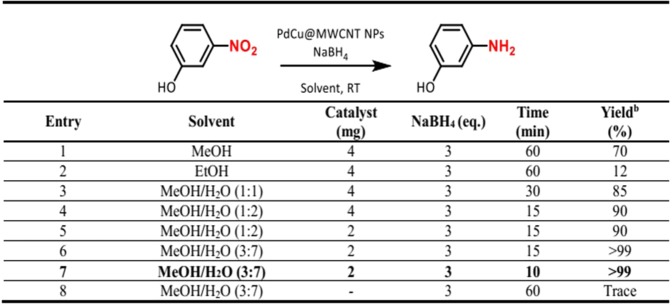
Optimization experiments for reduction of 3-nitrophenol to 3-aminophenol. (**a**) Reaction Conditions: 0.25 mmol substrate, PdCu@MWCNT catalyst (5% wt metal content), room temperature. (**b**) Determined by GC analysis.

The catalytic activity of the PdCu@MWCNT NPs was studied for the selective hydrogenation of 3-nitrophenol to 3-aminophenol in the presence of NaBH_4_ as a hydrogen source at room temperatures (Fig. 5). Firstly, different solvents such as methanol (MeOH), ethanol (EtOH) and H_2_O were tested. The H_2_O/MeOH mixture gave the best results. The compatibility of H_2_O/MeOH mixture with the substrate and product was also noteworthy. The addition of 3 mmol NaBH_4_ with 2 mg of catalyst in the presence of H_2_O/MeOH mixture showed a serious increase in the yield (Fig. 5, entry 5). Eventually, 0.25 mmol of substrate, 2.0 mg of catalyst and 0.75 mmol of NaBH_4_ gave sufficient performance for the conversion of nitroarenes into primary amines with only in 1.0 mL of water (Fig. 5, entry 7). However, there was no 3-aminophenol formation in the absence of catalyst and at room temperature (Fig. 5, entry 8). Some nitroarenes exhibited have high-performance reduction properties only in water. However, solubility problems in some substrates disrupt the standardization of the method.

**Figure 6:**
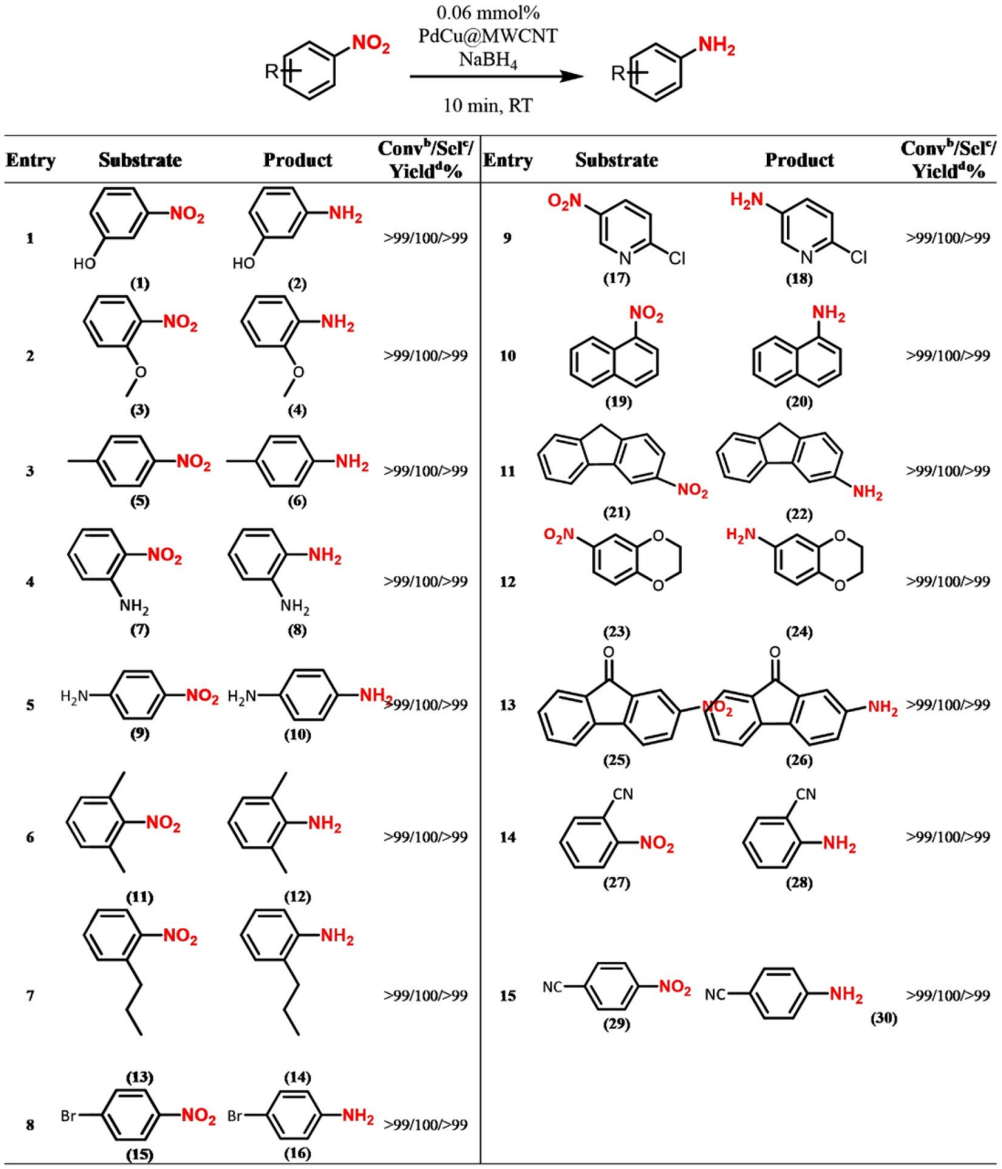
PdCu@MWCNT catalyzed reduction of various nitroarene compounds. (**a**) Reaction Conditions: 0.25 mmol substrate, 0.75 mmol NaBH_4_, 2 mg PdCu@MWCNT catalyst (5% wt metal content), 1 mL of water/methanol (v/v = 7/3), at room temperature. (**b**) GC conversion based on aromatic substrates. (**c**) Selectivity based on GC results. (**d**) GC yield.

Figure 6 summarizes the results obtained from PdCu@MWCNT catalyzed reduction reactions. In the series of nitroarene compounds tested, they were all reduced to the respective primary amine derivatives with excellent yields in 10 minutes at room temperature. The nitoarene derivatives containing electron-donor groups such as hydroxyl (-OH), methoxy (-OCH_3_), alkyl (-R) and amino (-NH_2_) at different positions were also reduced to the primary amine derivatives in high yields within 10 min of reaction time (Fig. 6, entries 1–7). 1-bromo-4-nitrobenzene (15) was converted to 4-bromoaniline (16) with high yields (Fig. 6, entry 8).

The PdCu@MWCNT NPs were also active in catalyzing hydrolysis of NaBH_4_ and hydrogenation of 2-chloro-5-nitropyridine (17), 2-nitro-naphthyl (19), 3-nitro-9H-fluorene (21) and 6-nitro-2,3-dihydrobenzo[b][1,4]dioxine (23) compounds. They were all converted to respective amine derivatives (18, 20, 22, 24) with the yields higher than 99% in 10 min (Fig. 6, entries 8–11).

**Figure 7:**
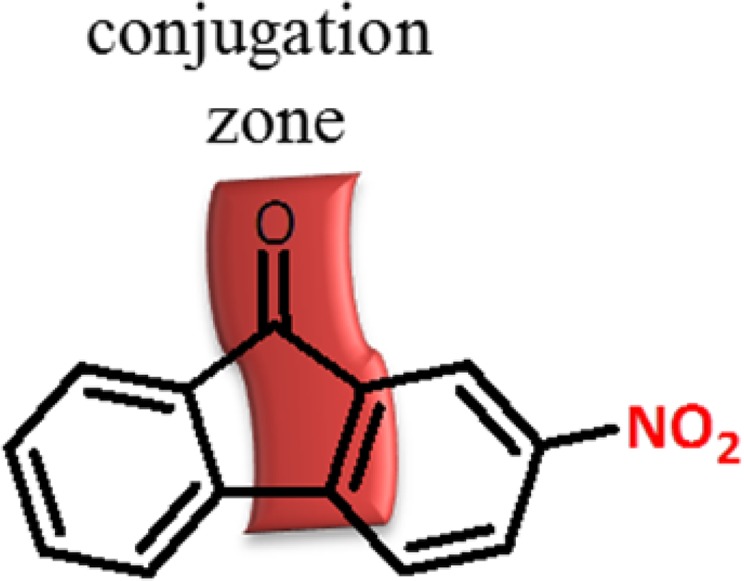
Schematic view for conjugation zone of 2-nitro-9H-fluorene-9-one.

2-nitro-9H-fluorene-9-one (25) was converted to 2-amino-9H-fluorene-9-one (26) at room temperature (Fig. 6, entry 12). However, the carbonyl group was not reduced due to conjugation (Fig. 7).

**Figure 8:**
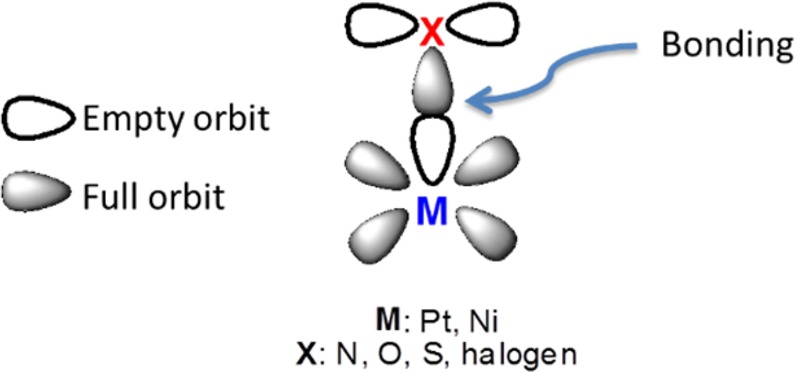
Schematic view of bonding between metal-ligand.

2-nitrobenzonitrile (27) and 4-nitrobenzonitrile (29) were optionally reduced to the corresponding primary amines (Fig. 6, entries 14, 15). Surprisingly, nitrile groups were not reduced and only nitro groups were reduced. This is very important for the selectivity of the catalyst. In the catalytic reactions, the binding event, ie the σ component, is often indispensable between the metal and the ligand (Fig. 8). As the binding event increases the time spent on the catalyst surface and around it of the nitroarene derivatives, the reaction efficiency is increased.

**Figure 9:**
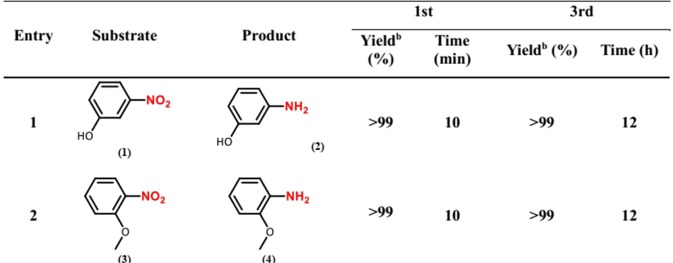
Reusability test of PdCu@MWCNT NPs. (**a**) Reaction Conditions: 0.25 mmol substrate, 0.75 mmol NaBH4, 2 mg PdCu@MWCNT catalyst (5% wt metal content), 1 mL of H_2_O/MeOH (v/v = 7/3), at room temperature. (**b**) GC yield.

Besides its high activity, the PdCu@MWCNT catalyst is also stable and reusable for the reduction reaction, providing ≤99% conversion after its 3^rd^ consecutive use in the reduction reaction of various compounds (Fig. 9). There is no noticeable loss of palladium and copper (0.7 ppm and 0.9 ppm leaching to a solution respectively) after five cycles reusability test confirmed by the ICP-OES analyses.

## Conclusions

The reduction of nitroarene compounds for organic synthesis and industrial applications has gained great importance when done in a low cost and easy way. In this study, we synthesized PdCu@MWCNT nanocatalyst which was synthesized for hydrogenation of nitroarenes and it was stated that it is a new method for hydrogenation of nitroarenes. The catalytic activity of PdCu@MWCNT NPs was investigated for selective hydrogenation of 3-nitrophenol to 3-aminophenol in the presence of NaBH4 as a hydrogen source at room temperature. Some nitroarenes have been found to have high-performance reduction properties only in water. In the series of nitroarene compounds tested, all were reduced to the corresponding primary amine derivatives in excellent yields within 10 minutes at room temperature. Furthermore, they were all successfully converted to the corresponding amine derivatives during this time, yielding greater than 99%. Surprisingly, the nanocatalyst did not play a role in the reduction of nitrile groups, which resulted in the removal of nitro groups. This is very important for the catalyst selectivity of the catalyst. In catalytic reactions, the binding event, ie the σ component, is generally indispensable between the metal and the ligand. It has been observed that as the bonding event increases the time spent on the surface of the catalyst and around the nitroarene derivatives, the reaction efficiency increases. In addition to its high activity, the PdCu@MWCNT catalyst was determined to be stable and reusable for the reaction, providing 99% conversion after the 3rd consecutive use of various compounds in the reduction reaction.

## Supplementary information

Supplementary Information.
