# Supplementary material for: A Novel Hydrogenation of Nitroarene Compounds with Multi Wall Carbon Nanotube Supported Palladium/Copper Nanoparticles (PdCu@MWCNT NPs) in Aqueous Medium
Source: Sci Rep. 2020 May 15;10:8043. doi: 10.1038/s41598-020-64988-0 (PMC7229225; doi:10.1038/s41598-020-64988-0)
Supplement: Supplementary file 1 — Supplementary Information. [file 41598_2020_64988_MOESM1_ESM.docx]

**SUPPORTING INFORMATION**

**A Novel Hydrogenation of Nitroarene Compounds with Multi Wall Carbon Nanotube Supported Palladium/Copper Nanoparticles (****PdCu@MWCNT NPs) in Aqueous Medium**

Haydar Göksu^a,^*, Nursefa Zengin^a^, Hakan Burhan^b^, Kemal Cellat^b^, Fatih Şen^b^*

^a^Kaynasli Vocational College, Düzce University, Düzce 81900, Turkey

^b^Sen Research Group, Department of Biochemistry, Dumlupınar

University, 43100 Kütahya, Turkey

^*^Corresponding authors: haydargoksu@duzce.edu.tr, fatihsen1980@gmail.com

**The materials and characterization of PdCu@MWCNT nanohybrids**

The synthesis was carried out using standard airless procedures and commercially available reagents. All the reagents were used as received. PdCl_2_, Cu_2_O and all nitro arene compounds tested in the reduction reactions were purchased from Sigma-Aldrich.

The characterization of monodisperse PdCu@MWCNT nanocatalyst was performed via TEM, XRD, XPS, Raman Spectroscopy. TEM analysis of PdCu@MWCNT nanocatalyst achieved by a JEOL 200 kV TEM instrument. Sample preparation was carried out through the suspension of about 0.5 mg catalyst in 3 ml of ethanol in an ultrasonic bath, and a drop of the resulting solution on a copper grid made of 400 mesh carbon. Approximately, 300 particles were investigated to the average particle size. XRD (X-ray diffraction) analysis was performed to investigate the crystal structure of the PdCu@MWCNT nanocatalyst. Panalytical Empyrean Diffractometer was employed for the XRD analysis, (λ = 1.54056Å, Cu K radiation) at 40 mA - 45 Kv. Oxidation levels of palladium and copper metals in the PdCu@MWCNT nanocatalyst and the surface composition of the PdCu@MWCNT nanohybrids were investigated by XPS analysis using X-ray photoelectron spectrometer having X-ray source at 1253.6 eV, 10 mA on K lines of Mg. Gaussian-Lorentzian function was used to fitting the XPS peaks, and the C 1s line at 284.6 eV was taken as a reference for all the lines. ^1^H and ^13^C NMR spectra were recorded on a Jeol ECS 400 MHz spectrometer.


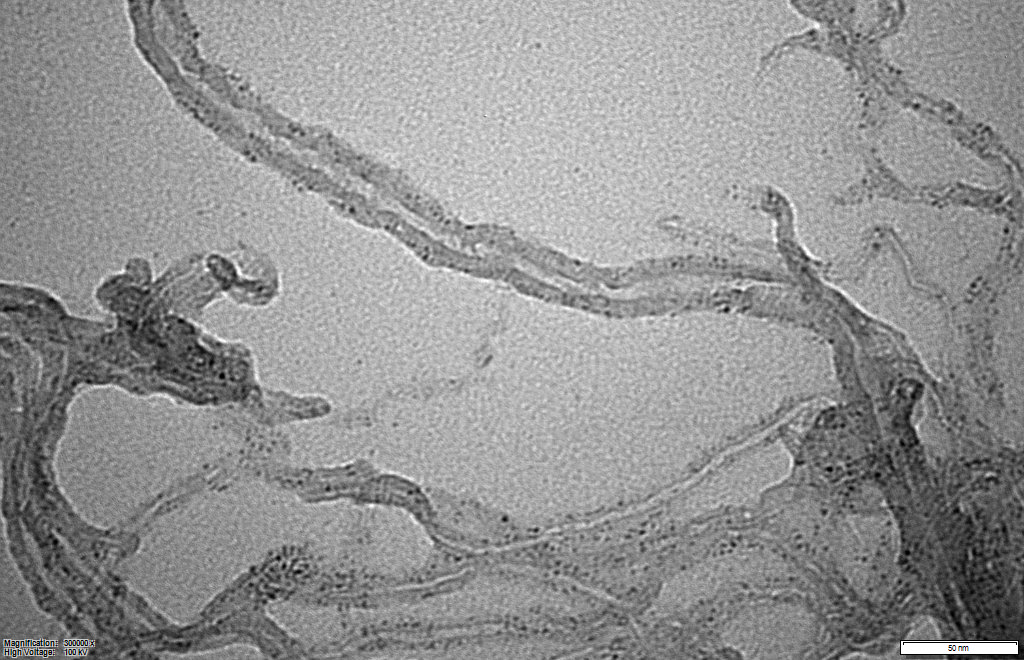


Fig. S1. PdCu@MWCNT Tem Image


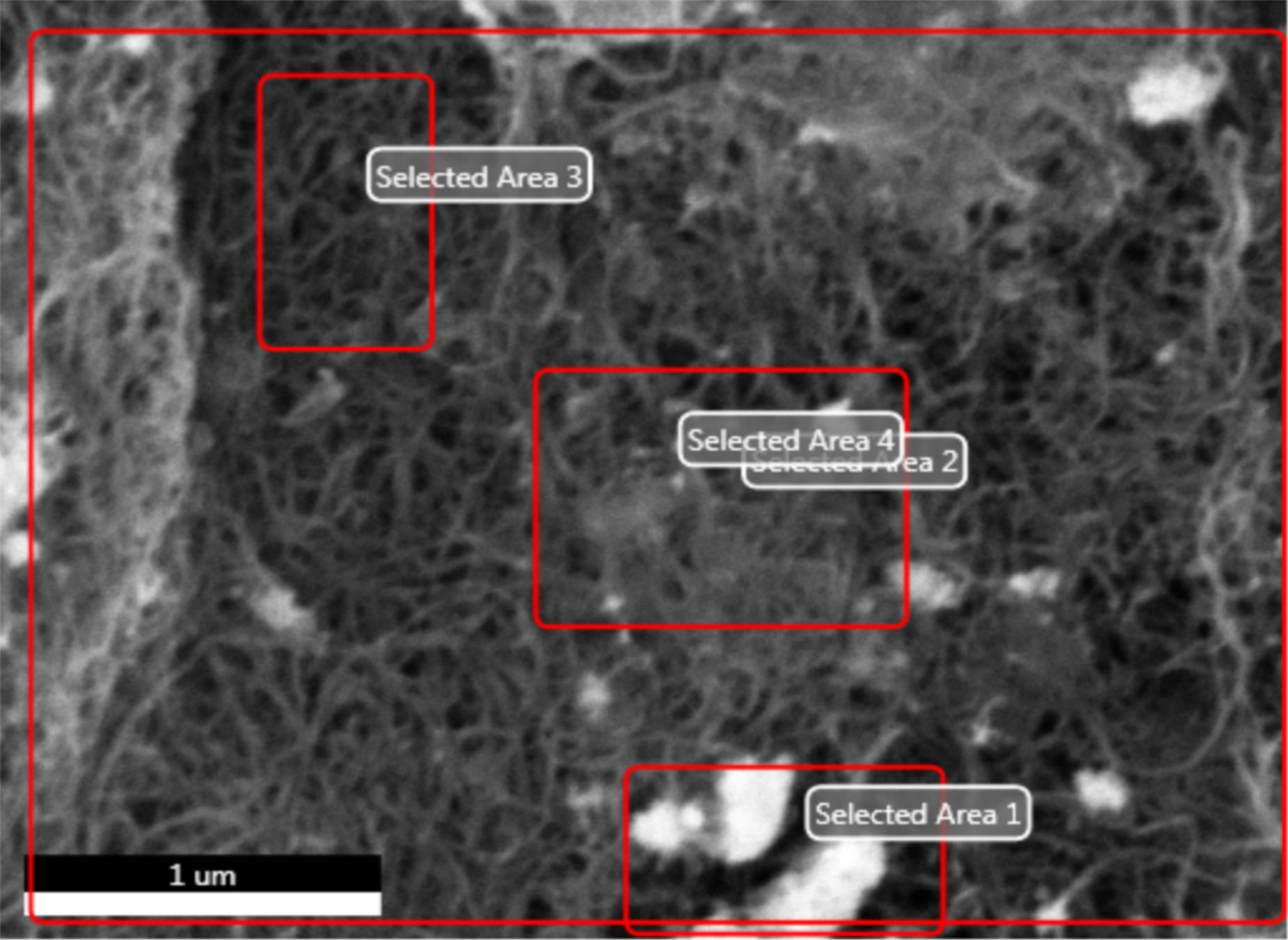


Fig. S2. Sem Edax

|  |  | **EDAX TEAM** | | | | | | | | | | | | | | | | | | | | | | | | | | |  | | | | | | | | | | | *Page* | *4* | | |  |  |  |  |
| --- | --- | --- | --- | --- | --- | --- | --- | --- | --- | --- | --- | --- | --- | --- | --- | --- | --- | --- | --- | --- | --- | --- | --- | --- | --- | --- | --- | --- | --- | --- | --- | --- | --- | --- | --- | --- | --- | --- | --- | --- | --- | --- | --- | --- | --- | --- | --- |
|  |  |  |  |  |  |  |  |  |  |  |  |  |  |  |  |  |  |  |  |  |  |  |  |  |  |  |  |  |  |  |  |  |  |  |  |  |  |  |  |  |  |  |  |  |  |  |  |
|  |  |  |  |  |  |  |  |  |  |  |  |  |  |  |  |  |  |  |  |  |  |  |  |  |  |  |  |  |  |  |  |  |  |  |  |  |  |  |  |  |  |  |  |  |  |  |  |
|  |  |  |  |  |  |  |  |  |  |  |  |  |  |  |  |  |  |  |  |  |  |  |  |  |  |  |  |  |  | | | | | | | | | | |  |  |  |  |  |  |  |  |
|  |  |  |  |  |  |  |  |  |  |  |  |  |  |  |  |  |  |  |  |  |  |  |  |  |  |  |  |  |  |  |  |  |  |  |  |  |  |  |  |  |  |  |  |  |  |  |  |
| 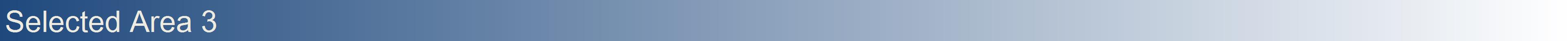 | | | | | | | | | | | | | | | | | | | | | | | | | | | | | | | | | | | | | | | | | | | | | | |  |
|  |  |  |  |  |  |  |  |  |  |  |  |  |  |  |  |  |  |  |  |  |  |  |  |  |  |  |  |  |  |  |  |  |  |  |  |  |  |  |  |  |  |  |  |  |  |  |  |
|  |  |  |  |  |  |  |  |  |  |  |  |  |  |  |  |  |  |  |  |  |  |  |  |  |  |  |  |  |  |  |  |  |  |  |  |  |  |  |  |  |  |  |  |  |  |  |  |
|  | kV: | |  |  | 20 |  |  |  |  | Mag: | | 100000 | |  | Takeoff: |  | 75.2 | |  |  |  | Live Time(s): | | |  | 29.5 |  |  |  | Amp Time(µs): | | | | | 7.68 | | |  | Resolution:(eV) | | | 126.2 | | | |  |  |
|  |  |  |  |  |  |  |  |  |  |  |  |  |  |  |  |  |  |  |  |  |  |  |  |  |  |  |  |  |  |  |  |  |  |  |  |  |  |  |  |  |  |  |  |  |  |  |  |
|  |  |  |  |  |  |  | **Selected Area 3 - EDS** | | | | | | | | | | | | | | | | | | | | | | | | | | | | | | | | | |  |  |  |  |  |  |  |
|  |  |  |  |  |  |  | 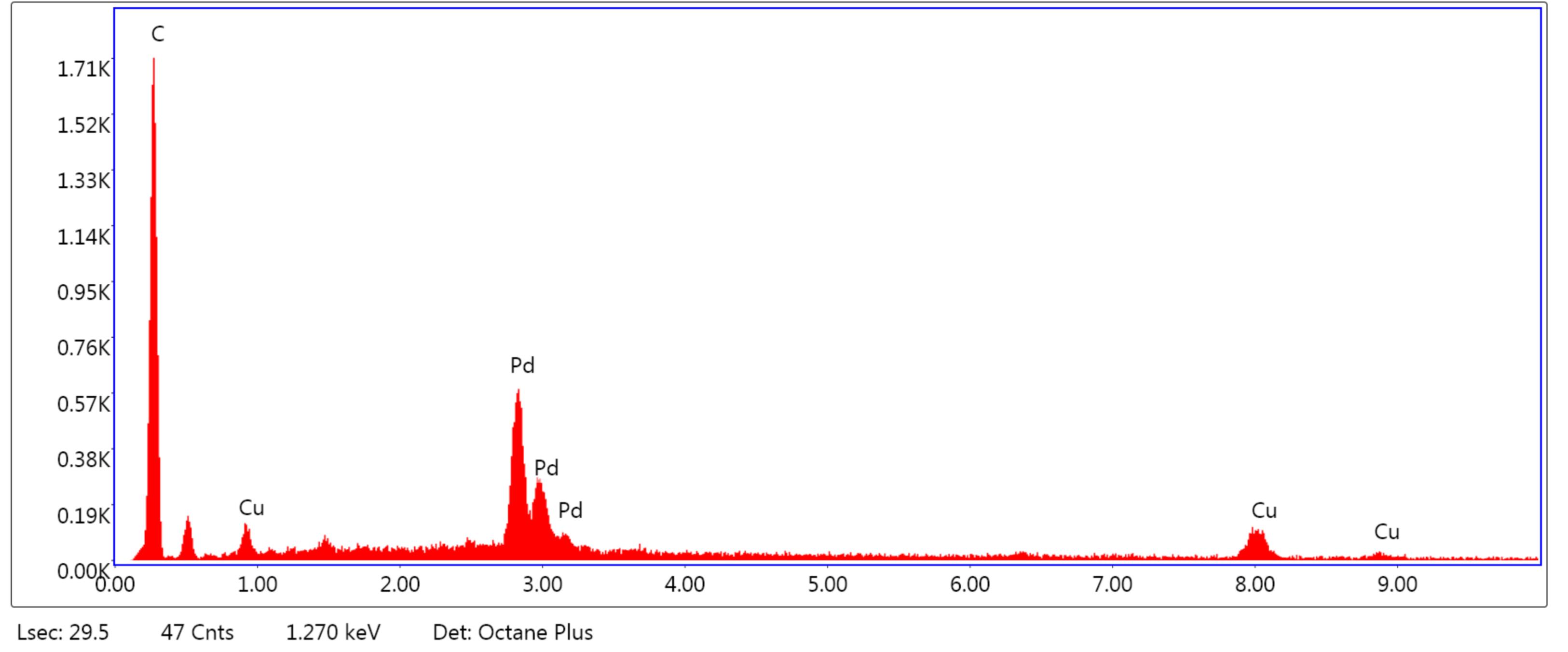 | | | | | | | | | | | | | | | | | | | | | | | | | | | | | | | | | |  |  |  |  |  |  |  |
|  |  |  |  |  |  |  |  |  |  |  |  |  |  |  |  |  |  |  |  |  |  |  |  |  |  |  |  |  |  |  |  |  |  |  |  |  |  |  |  |  |  |  |  |  |  |  |  |
|  |  |  |  |  |  |  |  |  |  |  |  |  |  |  |  |  |  |  |  |  |  |  |  |  |  |  |  |  |  |  |  |  |  |  |  |  |  |  |  |  |  |  |  |  |  |  |  |
|  |  |  |  |  |  |  |  |  |  |  |  |  |  |  |  |  |  |  |  |  |  |  |  |  |  |  |  |  |  |  |  |  |  |  |  |  |  |  |  |  |  |  |  |  |  |  |  |
|  |  |  |  | **eZAF Smart Quant Results** | | | | | | | | | | | | | | | | | | | | | | | | | | | | | | | | | | | | | | | |  |  |  |  |
|  |  |  |  |  |  |  |  |  |  |  |  |  |  |  |  |  |  |  |  |  |  |  |  |  |  |  |  |  |  |  |  |  |  |  |  |  |  |  |  |  |  |  |  |  |  |  |  |
|  |  |  |  |  |  |  |  |  |  |  |  |  |  |  |  |  |  | Element | | | Weight % | | | Atomic % | | | |  |  |  |  |  |  |  |  |  |  |  |  |  |  |  |  |  |  |  |  |
|  |  |  |  |  |  |  |  |  |  |  |  |  |  |  |  |  |  | C K | | | 42.67 | | | 84.10 | | | |  |  |  |  |  |  |  |  |  |  |  |  |  |  |  |  |  |  |  |  |
|  |  |  |  |  |  |  |  |  |  |  |  |  |  |  |  |  |  | PdL | | | 36.36 | | | 8.09 | | | |  |  |  |  |  |  |  |  |  |  |  |  |  |  |  |  |  |  |  |  |
|  |  |  |  |  |  |  |  |  |  |  |  |  |  |  |  |  |  | CuK | | | 20.96 | | | 7.81 | | | |  |  |  |  |  |  |  |  |  |  |  |  |  |  |  |  |  |  |  |  |

Fig. S3. Elemental Analysis

XPS Raw Data

Cu2p

| Binding Energy (E) |  |  |  | Backgnd. |
| --- | --- | --- | --- | --- |
| eV |  | Counts / s |  | Counts / s |
| 968,08 |  | 5355,94 |  | 0 |
| 967,98 |  | 5354,3 |  | 0 |
| 967,88 |  | 5352,31 |  | 5347,84 |
| 967,78 |  | 5350 |  | 5347,69 |
| 967,68 |  | 5347,35 |  | 5347,54 |
| 967,58 |  | 5344,35 |  | 5347,39 |
| 967,48 |  | 5341 |  | 5347,24 |
| 967,38 |  | 5337,49 |  | 5347,09 |
| 967,28 |  | 5333,92 |  | 5346,94 |
| 967,18 |  | 5330,4 |  | 5346,79 |
| 967,08 |  | 5327,06 |  | 5346,64 |
| 966,98 |  | 5324,05 |  | 5346,49 |
| 966,88 |  | 5321,48 |  | 5346,34 |
| 966,78 |  | 5319,37 |  | 5346,2 |
| 966,68 |  | 5317,76 |  | 5346,05 |
| 966,58 |  | 5316,78 |  | 5345,9 |
| 966,48 |  | 5316,56 |  | 5345,76 |
| 966,38 |  | 5317,2 |  | 5345,61 |
| 966,28 |  | 5318,77 |  | 5345,46 |
| 966,18 |  | 5321,24 |  | 5345,3 |
| 966,08 |  | 5324,62 |  | 5345,15 |
| 965,98 |  | 5328,86 |  | 5344,98 |
| 965,88 |  | 5333,9 |  | 5344,81 |
| 965,78 |  | 5339,74 |  | 5344,64 |
| 965,68 |  | 5346,44 |  | 5344,43 |
| 965,58 |  | 5354,01 |  | 5344,19 |
| 965,48 |  | 5362,38 |  | 5343,88 |
| 965,38 |  | 5371,52 |  | 5343,52 |
| 965,28 |  | 5381,41 |  | 5343,09 |
| 965,18 |  | 5392,13 |  | 5342,6 |
| 965,08 |  | 5403,72 |  | 5342,02 |
| 964,98 |  | 5416,19 |  | 5341,37 |
| 964,88 |  | 5429,54 |  | 5340,62 |
| 964,78 |  | 5443,77 |  | 5339,78 |
| 964,68 |  | 5458,85 |  | 5338,85 |
| 964,58 |  | 5474,64 |  | 5337,8 |
| 964,48 |  | 5491,01 |  | 5336,65 |
| 964,38 |  | 5507,85 |  | 5335,39 |
| 964,28 |  | 5524,97 |  | 5334,02 |
| 964,18 |  | 5542,09 |  | 5332,53 |
| 964,08 |  | 5558,86 |  | 5330,93 |
| 963,98 |  | 5574,99 |  | 5329,21 |
| 963,88 |  | 5590,22 |  | 5327,39 |
| 963,78 |  | 5604,28 |  | 5325,46 |
| 963,68 |  | 5616,86 |  | 5323,44 |
| 963,58 |  | 5627,65 |  | 5321,33 |
| 963,48 |  | 5636,47 |  | 5319,14 |
| 963,38 |  | 5643,16 |  | 5316,88 |
| 963,28 |  | 5647,53 |  | 5314,57 |
| 963,18 |  | 5649,51 |  | 5312,22 |
| 963,08 |  | 5649,09 |  | 5309,84 |
| 962,98 |  | 5646,36 |  | 5307,45 |
| 962,88 |  | 5641,35 |  | 5305,06 |
| 962,78 |  | 5634,14 |  | 5302,68 |
| 962,68 |  | 5624,82 |  | 5300,33 |
| 962,58 |  | 5613,61 |  | 5298,02 |
| 962,48 |  | 5600,81 |  | 5295,76 |
| 962,38 |  | 5586,6 |  | 5293,56 |
| 962,28 |  | 5571,18 |  | 5291,44 |
| 962,18 |  | 5554,77 |  | 5289,39 |
| 962,08 |  | 5537,65 |  | 5287,43 |
| 961,98 |  | 5520,05 |  | 5285,56 |
| 961,88 |  | 5502,15 |  | 5283,79 |
| 961,78 |  | 5484,17 |  | 5282,12 |
| 961,68 |  | 5466,34 |  | 5280,54 |
| 961,58 |  | 5448,92 |  | 5279,07 |
| 961,48 |  | 5432,01 |  | 5277,69 |
| 961,38 |  | 5415,73 |  | 5276,41 |
| 961,28 |  | 5400,21 |  | 5275,22 |
| 961,18 |  | 5385,61 |  | 5274,12 |
| 961,08 |  | 5372,02 |  | 5273,11 |
| 960,98 |  | 5359,47 |  | 5272,17 |
| 960,88 |  | 5347,98 |  | 5271,31 |
| 960,78 |  | 5337,6 |  | 5270,51 |
| 960,68 |  | 5328,32 |  | 5269,78 |
| 960,58 |  | 5320,09 |  | 5269,1 |
| 960,48 |  | 5312,81 |  | 5268,47 |
| 960,38 |  | 5306,43 |  | 5267,88 |
| 960,28 |  | 5300,83 |  | 5267,33 |
| 960,18 |  | 5295,89 |  | 5266,82 |
| 960,08 |  | 5291,45 |  | 5266,34 |
| 959,98 |  | 5287,4 |  | 5265,89 |
| 959,88 |  | 5283,66 |  | 5265,45 |
| 959,78 |  | 5280,14 |  | 5265,05 |
| 959,68 |  | 5276,79 |  | 5264,66 |
| 959,58 |  | 5273,57 |  | 5264,28 |
| 959,48 |  | 5270,45 |  | 5263,93 |
| 959,38 |  | 5267,43 |  | 5263,59 |
| 959,28 |  | 5264,54 |  | 5263,26 |
| 959,18 |  | 5261,86 |  | 5262,95 |
| 959,08 |  | 5259,46 |  | 5262,64 |
| 958,98 |  | 5257,47 |  | 5262,35 |
| 958,88 |  | 5255,97 |  | 5262,06 |
| 958,78 |  | 5255,07 |  | 5261,76 |
| 958,68 |  | 5254,84 |  | 5261,47 |
| 958,58 |  | 5255,29 |  | 5261,18 |
| 958,48 |  | 5256,43 |  | 5260,87 |
| 958,38 |  | 5258,28 |  | 5260,56 |
| 958,28 |  | 5260,84 |  | 5260,23 |
| 958,18 |  | 5264,09 |  | 5259,87 |
| 958,08 |  | 5268,02 |  | 5259,49 |
| 957,98 |  | 5272,64 |  | 5259,08 |
| 957,88 |  | 5277,93 |  | 5258,63 |
| 957,78 |  | 5283,85 |  | 5258,15 |
| 957,68 |  | 5290,38 |  | 5257,62 |
| 957,58 |  | 5297,52 |  | 5257,04 |
| 957,48 |  | 5305,38 |  | 5256,4 |
| 957,38 |  | 5314,07 |  | 5255,7 |
| 957,28 |  | 5323,72 |  | 5254,93 |
| 957,18 |  | 5334,51 |  | 5254,09 |
| 957,08 |  | 5346,58 |  | 5253,17 |
| 956,98 |  | 5360,05 |  | 5252,15 |
| 956,88 |  | 5374,97 |  | 5251,04 |
| 956,78 |  | 5391,37 |  | 5249,81 |
| 956,68 |  | 5409,28 |  | 5248,47 |
| 956,58 |  | 5428,72 |  | 5247,01 |
| 956,48 |  | 5449,58 |  | 5245,4 |
| 956,38 |  | 5471,66 |  | 5243,66 |
| 956,28 |  | 5494,69 |  | 5241,77 |
| 956,18 |  | 5518,36 |  | 5239,72 |
| 956,08 |  | 5542,22 |  | 5237,52 |
| 955,98 |  | 5565,78 |  | 5235,16 |
| 955,88 |  | 5588,58 |  | 5232,64 |
| 955,78 |  | 5610,16 |  | 5229,97 |
| 955,68 |  | 5630,05 |  | 5227,16 |
| 955,58 |  | 5647,77 |  | 5224,22 |
| 955,48 |  | 5662,88 |  | 5221,15 |
| 955,38 |  | 5675,03 |  | 5217,98 |
| 955,28 |  | 5684,01 |  | 5214,71 |
| 955,18 |  | 5689,64 |  | 5211,37 |
| 955,08 |  | 5691,81 |  | 5207,98 |
| 954,98 |  | 5690,6 |  | 5204,54 |
| 954,88 |  | 5686,16 |  | 5201,09 |
| 954,78 |  | 5678,71 |  | 5197,64 |
| 954,68 |  | 5668,56 |  | 5194,21 |
| 954,58 |  | 5656,17 |  | 5190,81 |
| 954,48 |  | 5642,01 |  | 5187,46 |
| 954,38 |  | 5626,58 |  | 5184,16 |
| 954,28 |  | 5610,39 |  | 5180,93 |
| 954,18 |  | 5593,87 |  | 5177,77 |
| 954,08 |  | 5577,48 |  | 5174,69 |
| 953,98 |  | 5561,69 |  | 5171,68 |
| 953,88 |  | 5546,79 |  | 5168,74 |
| 953,78 |  | 5533,08 |  | 5165,87 |
| 953,68 |  | 5520,8 |  | 5163,07 |
| 953,58 |  | 5510,03 |  | 5160,33 |
| 953,48 |  | 5500,72 |  | 5157,65 |
| 953,38 |  | 5492,76 |  | 5155,01 |
| 953,28 |  | 5485,96 |  | 5152,41 |
| 953,18 |  | 5480,08 |  | 5149,85 |
| 953,08 |  | 5474,85 |  | 5147,32 |
| 952,98 |  | 5469,91 |  | 5144,82 |
| 952,88 |  | 5464,85 |  | 5142,34 |
| 952,78 |  | 5459,27 |  | 5139,9 |
| 952,68 |  | 5452,74 |  | 5137,49 |
| 952,58 |  | 5444,82 |  | 5135,12 |
| 952,48 |  | 5435,19 |  | 5132,8 |
| 952,38 |  | 5423,6 |  | 5130,53 |
| 952,28 |  | 5409,86 |  | 5128,33 |
| 952,18 |  | 5393,85 |  | 5126,21 |
| 952,08 |  | 5375,53 |  | 5124,19 |
| 951,98 |  | 5354,92 |  | 5122,26 |
| 951,88 |  | 5332,19 |  | 5120,46 |
| 951,78 |  | 5307,61 |  | 5118,78 |
| 951,68 |  | 5281,51 |  | 5117,24 |
| 951,58 |  | 5254,28 |  | 5115,85 |
| 951,48 |  | 5226,38 |  | 5114,62 |
| 951,38 |  | 5198,23 |  | 5113,55 |
| 951,28 |  | 5170,23 |  | 5112,64 |
| 951,18 |  | 5142,77 |  | 5111,89 |
| 951,08 |  | 5116,21 |  | 5111,3 |
| 950,98 |  | 5090,85 |  | 5110,88 |
| 950,88 |  | 5066,92 |  | 5110,59 |
| 950,78 |  | 5044,55 |  | 5110,36 |
| 950,68 |  | 5023,79 |  | 5110,18 |
| 950,58 |  | 5004,67 |  | 5110,02 |
| 950,48 |  | 4987,18 |  | 5109,87 |
| 950,38 |  | 4971,26 |  | 5109,75 |
| 950,28 |  | 4956,83 |  | 5109,63 |
| 950,18 |  | 4943,82 |  | 5109,53 |
| 950,08 |  | 4932,11 |  | 5109,44 |
| 949,98 |  | 4921,57 |  | 5109,37 |
| 949,88 |  | 4912,16 |  | 5109,29 |
| 949,78 |  | 4903,8 |  | 5109,22 |
| 949,68 |  | 4896,5 |  | 5109,15 |
| 949,58 |  | 4890,24 |  | 5109,08 |
| 949,48 |  | 4884,96 |  | 5109,02 |
| 949,38 |  | 4880,62 |  | 5108,95 |
| 949,28 |  | 4877,14 |  | 5108,88 |
| 949,18 |  | 4874,42 |  | 5108,82 |
| 949,08 |  | 4872,38 |  | 5108,75 |
| 948,98 |  | 4871,02 |  | 5108,69 |
| 948,88 |  | 4870,28 |  | 5108,63 |
| 948,78 |  | 4870,09 |  | 5108,56 |
| 948,68 |  | 4870,4 |  | 9981 |
| 948,58 |  | 4871,12 |  | 9980,88 |
| 948,48 |  | 4872,17 |  | 9980,75 |
| 948,38 |  | 4873,51 |  | 9980,62 |
| 948,28 |  | 4875,1 |  | 9980,49 |
| 948,18 |  | 4876,95 |  | 9980,36 |
| 948,08 |  | 4879,12 |  | 9980,21 |
| 947,98 |  | 4881,65 |  | 9980,06 |
| 947,88 |  | 4884,47 |  | 9979,91 |
| 947,78 |  | 4887,56 |  | 9979,74 |
| 947,68 |  | 4890,93 |  | 9979,56 |
| 947,58 |  | 4894,6 |  | 9979,37 |
| 947,48 |  | 4898,54 |  | 9979,16 |
| 947,38 |  | 4902,81 |  | 9978,94 |
| 947,28 |  | 4907,46 |  | 9978,71 |
| 947,18 |  | 4912,57 |  | 9978,45 |
| 947,08 |  | 4918,23 |  | 9978,18 |
| 946,98 |  | 4924,48 |  | 9977,87 |
| 946,88 |  | 4931,43 |  | 9977,54 |
| 946,78 |  | 4939,28 |  | 9977,17 |
| 946,68 |  | 4948,24 |  | 9976,77 |
| 946,58 |  | 4958,48 |  | 9976,32 |
| 946,48 |  | 4970,19 |  | 9975,83 |
| 946,38 |  | 4983,6 |  | 9975,28 |
| 946,28 |  | 4998,84 |  | 9974,67 |
| 946,18 |  | 5016,04 |  | 9974 |
| 946,08 |  | 5035,24 |  | 9973,26 |
| 945,98 |  | 5056,38 |  | 9972,41 |
| 945,88 |  | 5079,43 |  | 9971,46 |
| 945,78 |  | 5104,26 |  | 9970,36 |
| 945,68 |  | 5130,61 |  | 9969,05 |
| 945,58 |  | 5158,09 |  | 9967,5 |
| 945,48 |  | 5186,34 |  | 9965,68 |
| 945,38 |  | 5214,93 |  | 9963,61 |
| 945,28 |  | 5243,33 |  | 9961,27 |
| 945,18 |  | 5271,08 |  | 9958,66 |
| 945,08 |  | 5297,67 |  | 9955,8 |
| 944,98 |  | 5322,62 |  | 9952,68 |
| 944,88 |  | 5345,55 |  | 9949,32 |
| 944,78 |  | 5366,11 |  | 9945,74 |
| 944,68 |  | 5383,97 |  | 9941,96 |
| 944,58 |  | 5398,99 |  | 9937,99 |
| 944,48 |  | 5411,17 |  | 9933,86 |
| 944,38 |  | 5420,51 |  | 9929,58 |
| 944,28 |  | 5427,12 |  | 9925,19 |
| 944,18 |  | 5431,19 |  | 9920,71 |
| 944,08 |  | 5432,98 |  | 9916,15 |
| 943,98 |  | 5432,81 |  | 9911,54 |
| 943,88 |  | 5431,03 |  | 9906,89 |
| 943,78 |  | 5428,01 |  | 9902,22 |
| 943,68 |  | 5424,11 |  | 9897,54 |
| 943,58 |  | 5419,65 |  | 9892,86 |
| 943,48 |  | 5414,88 |  | 9888,19 |
| 943,38 |  | 5409,94 |  | 9883,53 |
| 943,28 |  | 5405,01 |  | 9878,89 |
| 943,18 |  | 5400,18 |  | 9874,27 |
| 943,08 |  | 5395,51 |  | 9869,67 |
| 942,98 |  | 5390,99 |  | 9865,09 |
| 942,88 |  | 5386,55 |  | 9860,54 |
| 942,78 |  | 5382,1 |  | 9856,01 |
| 942,68 |  | 5377,49 |  | 9851,51 |
| 942,58 |  | 5372,56 |  | 9847,03 |
| 942,48 |  | 5367,21 |  | 9842,58 |
| 942,38 |  | 5361,36 |  | 9838,17 |
| 942,28 |  | 5354,95 |  | 9833,8 |
| 942,18 |  | 5347,96 |  | 9829,46 |
| 942,08 |  | 5340,31 |  | 9825,18 |
| 941,98 |  | 5331,96 |  | 9820,95 |
| 941,88 |  | 5322,91 |  | 9816,78 |
| 941,78 |  | 5313,14 |  | 9812,68 |
| 941,68 |  | 5302,69 |  | 9808,64 |
| 941,58 |  | 5291,6 |  | 9804,69 |
| 941,48 |  | 5279,95 |  | 9800,83 |
| 941,38 |  | 5267,78 |  | 9797,05 |
| 941,28 |  | 5255,13 |  | 9793,37 |
| 941,18 |  | 5242,01 |  | 9789,79 |
| 941,08 |  | 5228,44 |  | 9786,32 |
| 940,98 |  | 5214,48 |  | 9782,96 |
| 940,88 |  | 5200,24 |  | 9779,71 |
| 940,78 |  | 5185,86 |  | 9776,58 |
| 940,68 |  | 5171,46 |  | 9773,56 |
| 940,58 |  | 5157,12 |  | 9770,67 |
| 940,48 |  | 5143 |  | 9767,89 |
| 940,38 |  | 5129,2 |  | 9765,22 |
| 940,28 |  | 5115,88 |  | 9762,67 |
| 940,18 |  | 5103,15 |  | 9760,22 |
| 940,08 |  | 5091,22 |  | 9757,88 |
| 939,98 |  | 5080,24 |  | 9755,64 |
| 939,88 |  | 5070,33 |  | 9753,48 |
| 939,78 |  | 5061,57 |  | 9751,4 |
| 939,68 |  | 5053,98 |  | 9749,4 |
| 939,58 |  | 5047,59 |  | 9747,45 |
| 939,48 |  | 5042,48 |  | 9745,55 |
| 939,38 |  | 5038,63 |  | 9743,69 |
| 939,28 |  | 5036,04 |  | 9741,86 |
| 939,18 |  | 5034,69 |  | 9740,04 |
| 939,08 |  | 5034,53 |  | 9738,23 |
| 938,98 |  | 5035,43 |  | 9736,41 |
| 938,88 |  | 5037,35 |  | 9734,58 |
| 938,78 |  | 5040,23 |  | 9732,72 |
| 938,68 |  | 5044,03 |  | 9730,82 |
| 938,58 |  | 5048,78 |  | 9728,87 |
| 938,48 |  | 5054,47 |  | 9726,87 |
| 938,38 |  | 5061,1 |  | 9724,8 |
| 938,28 |  | 5068,74 |  | 9722,65 |
| 938,18 |  | 5077,42 |  | 9720,41 |
| 938,08 |  | 5087,28 |  | 9718,07 |
| 937,98 |  | 5098,47 |  | 9715,62 |
| 937,88 |  | 5111,19 |  | 9713,03 |
| 937,78 |  | 5125,62 |  | 9710,3 |
| 937,68 |  | 5141,97 |  | 9707,41 |
| 937,58 |  | 5160,45 |  | 9704,33 |
| 937,48 |  | 5181,3 |  | 9701,05 |
| 937,38 |  | 5204,76 |  | 9697,53 |
| 937,28 |  | 5231,12 |  | 9693,76 |
| 937,18 |  | 5260,57 |  | 9689,71 |
| 937,08 |  | 5293,31 |  | 9685,34 |
| 936,98 |  | 5329,49 |  | 9680,63 |
| 936,88 |  | 5369,21 |  | 9675,55 |
| 936,78 |  | 5412,47 |  | 9670,05 |
| 936,68 |  | 5459,19 |  | 9664,12 |
| 936,58 |  | 5509,22 |  | 9657,71 |
| 936,48 |  | 5562,26 |  | 9650,81 |
| 936,38 |  | 5617,86 |  | 9643,38 |
| 936,28 |  | 5675,4 |  | 9635,41 |
| 936,18 |  | 5734,1 |  | 9626,88 |
| 936,08 |  | 5793,13 |  | 9617,78 |
| 935,98 |  | 5851,52 |  | 9608,12 |
| 935,88 |  | 5908,24 |  | 9597,89 |
| 935,78 |  | 5962,17 |  | 9587,12 |
| 935,68 |  | 6012,22 |  | 9575,83 |
| 935,58 |  | 6057,37 |  | 9564,06 |
| 935,48 |  | 6096,62 |  | 9551,83 |
| 935,38 |  | 6129,09 |  | 9539,21 |
| 935,28 |  | 6154,15 |  | 9526,25 |
| 935,18 |  | 6171,36 |  | 9513 |
| 935,08 |  | 6180,56 |  | 9499,54 |
| 934,98 |  | 6181,78 |  | 9485,93 |
| 934,88 |  | 6175,27 |  | 9472,23 |
| 934,78 |  | 6161,58 |  | 9458,5 |
| 934,68 |  | 6141,45 |  | 9444,81 |
| 934,58 |  | 6115,79 |  | 9431,22 |
| 934,48 |  | 6085,66 |  | 9417,76 |
| 934,38 |  | 6052,29 |  | 9404,47 |
| 934,28 |  | 6016,99 |  | 9391,39 |
| 934,18 |  | 5981,05 |  | 9378,54 |
| 934,08 |  | 5945,67 |  | 9365,92 |
| 933,98 |  | 5911,93 |  | 9353,53 |
| 933,88 |  | 5880,74 |  | 9341,38 |
| 933,78 |  | 5852,85 |  | 9329,43 |
| 933,68 |  | 5828,72 |  | 9317,69 |
| 933,58 |  | 5808,58 |  | 9306,11 |
| 933,48 |  | 5792,35 |  | 9294,68 |
| 933,38 |  | 5779,66 |  | 9283,38 |
| 933,28 |  | 5769,75 |  | 9272,18 |
| 933,18 |  | 5761,54 |  | 9261,08 |
| 933,08 |  | 5753,77 |  | 9250,05 |
| 932,98 |  | 5745,11 |  | 9239,12 |
| 932,88 |  | 5734,22 |  | 9228,28 |
| 932,78 |  | 5719,79 |  | 9217,55 |
| 932,68 |  | 5700,62 |  | 9206,97 |
| 932,58 |  | 5675,67 |  | 9196,57 |
| 932,48 |  | 5644,15 |  | 9186,4 |
| 932,38 |  | 5605,6 |  | 9176,51 |
| 932,28 |  | 5559,96 |  | 9166,95 |
| 932,18 |  | 5507,62 |  | 9157,77 |
| 932,08 |  | 5449,39 |  | 9149,02 |
| 931,98 |  | 5386,38 |  | 9140,75 |
| 931,88 |  | 5319,87 |  | 9133 |
| 931,78 |  | 5251,26 |  | 9125,78 |
| 931,68 |  | 5182,02 |  | 9119,14 |
| 931,58 |  | 5113,59 |  | 9113,07 |
| 931,48 |  | 5047,38 |  | 9107,57 |
| 931,38 |  | 4984,66 |  | 9102,63 |
| 931,28 |  | 4926,42 |  | 9098,23 |
| 931,18 |  | 4873,41 |  | 9094,33 |
| 931,08 |  | 4826,04 |  | 9090,92 |
| 930,98 |  | 4784,42 |  | 9087,93 |
| 930,88 |  | 4748,43 |  | 9085,34 |
| 930,78 |  | 4717,79 |  | 9083,1 |
| 930,68 |  | 4692,05 |  | 9081,16 |
| 930,58 |  | 4670,68 |  | 9079,48 |
| 930,48 |  | 4653,07 |  | 9078,03 |
| 930,38 |  | 4638,58 |  | 9076,77 |
| 930,28 |  | 4626,56 |  | 9075,68 |
| 930,18 |  | 4616,49 |  | 9074,72 |
| 930,08 |  | 4607,87 |  | 9073,87 |
| 929,98 |  | 4600,3 |  | 9073,13 |
| 929,88 |  | 4593,5 |  | 9072,47 |
| 929,78 |  | 4587,26 |  | 9071,88 |
| 929,68 |  | 4581,41 |  | 9071,36 |
| 929,58 |  | 4575,83 |  | 9070,9 |
| 929,48 |  | 4570,49 |  | 9070,5 |
| 929,38 |  | 4565,39 |  | 9070,14 |
| 929,28 |  | 4560,56 |  | 9069,83 |
| 929,18 |  | 4556,05 |  | 9069,57 |
| 929,08 |  | 4551,87 |  | 9069,35 |
| 928,98 |  | 4548,07 |  | 9069,16 |
| 928,88 |  | 4544,67 |  | 9069,01 |
| 928,78 |  | 4541,7 |  | 9068,89 |
| 928,68 |  | 4539,18 |  | 9068,8 |
| 928,58 |  | 4537,12 |  | 9068,73 |
| 928,48 |  | 4535,57 |  | 9068,68 |
| 928,38 |  | 4534,5 |  | 9068,65 |
| 928,28 |  | 4533,89 |  | 9068,63 |
| 928,18 |  | 4533,69 |  | 9068,61 |
| 928,08 |  | 4533,87 |  | 9068,6 |
| 927,98 |  | 4534,37 |  | 9068,59 |
| 927,88 |  | 4535,13 |  | 9068,57 |
| 927,78 |  | 4536,07 |  | 9068,56 |
| 927,68 |  | 4537,14 |  | 9068,53 |
| 927,58 |  | 4538,21 |  | 9068,51 |
| 927,48 |  | 4539,2 |  | 9068,47 |
| 927,38 |  | 4539,98 |  | 9068,43 |
| 927,28 |  | 4540,53 |  | 9068,39 |
| 927,18 |  | 4540,77 |  | 9068,34 |
| 927,08 |  | 4540,67 |  | 9068,29 |
| 926,98 |  | 4540,24 |  | 9068,24 |
| 926,88 |  | 4539,55 |  | 9068,19 |
| 926,78 |  | 4538,61 |  | 9068,15 |
| 926,68 |  | 4537,48 |  | 9068,11 |
| 926,58 |  | 4536,22 |  | 9068,09 |
| 926,48 |  | 4534,94 |  | 9068,06 |
| 926,38 |  | 4533,78 |  | 9068,04 |
| 926,28 |  | 4532,88 |  | 9068,02 |
| 926,18 |  | 4532,3 |  | 9068,01 |
| 926,08 |  | 4532,13 |  | 9068,01 |
| 925,98 |  | 4532,39 |  | 0 |
| 925,88 |  | 4533,05 |  | 0 |
| 925,78 |  | 4534,05 |  | 0 |
| 925,68 |  | 4535,35 |  | 0 |
| 925,58 |  | 4536,95 |  | 0 |
| 925,48 |  | 4538,61 |  | 0 |
| 925,38 |  | 4540,28 |  | 0 |
| 925,28 |  | 4541,88 |  | 0 |
| 925,18 |  | 4543,34 |  | 0 |
| 925,08 |  | 4544,61 |  | 0 |

Pd3d

| Binding Energy (E) |  |  |  | Backgnd. |
| --- | --- | --- | --- | --- |
| eV |  | Counts / s |  | Counts / s |
| 348,08 |  | 2677,01 |  | 5362,69 |
| 347,98 |  | 2678,34 |  | 5362,58 |
| 347,88 |  | 2679,98 |  | 5362,44 |
| 347,78 |  | 2681,92 |  | 5362,28 |
| 347,68 |  | 2684,16 |  | 5362,11 |
| 347,58 |  | 2686,68 |  | 5361,91 |
| 347,48 |  | 2689,48 |  | 5361,69 |
| 347,38 |  | 2692,38 |  | 5361,44 |
| 347,28 |  | 2695,31 |  | 5361,15 |
| 347,18 |  | 2698,14 |  | 5360,82 |
| 347,08 |  | 2700,75 |  | 5360,46 |
| 346,98 |  | 2703,06 |  | 5360,07 |
| 346,88 |  | 2704,99 |  | 5359,64 |
| 346,78 |  | 2706,46 |  | 5359,19 |
| 346,68 |  | 2707,43 |  | 5358,72 |
| 346,58 |  | 2707,91 |  | 5358,23 |
| 346,48 |  | 2707,85 |  | 5357,73 |
| 346,38 |  | 2707,27 |  | 5357,23 |
| 346,28 |  | 2706,19 |  | 5356,72 |
| 346,18 |  | 2704,68 |  | 5356,22 |
| 346,08 |  | 2702,91 |  | 5355,73 |
| 345,98 |  | 2701,03 |  | 5355,24 |
| 345,88 |  | 2699,14 |  | 5354,77 |
| 345,78 |  | 2697,37 |  | 5354,31 |
| 345,68 |  | 2695,83 |  | 5353,86 |
| 345,58 |  | 2694,61 |  | 5353,42 |
| 345,48 |  | 2693,74 |  | 5352,99 |
| 345,38 |  | 2693,32 |  | 5352,56 |
| 345,28 |  | 2693,4 |  | 5352,13 |
| 345,18 |  | 2694 |  | 5351,69 |
| 345,08 |  | 2695,1 |  | 5351,25 |
| 344,98 |  | 2696,59 |  | 5350,78 |
| 344,88 |  | 2698,36 |  | 5350,3 |
| 344,78 |  | 2700,34 |  | 5349,79 |
| 344,68 |  | 2702,44 |  | 5349,26 |
| 344,58 |  | 2704,58 |  | 5348,69 |
| 344,48 |  | 2706,7 |  | 5348,1 |
| 344,38 |  | 2708,77 |  | 5347,48 |
| 344,28 |  | 2710,69 |  | 5346,83 |
| 344,18 |  | 2712,38 |  | 5346,15 |
| 344,08 |  | 2713,76 |  | 5345,44 |
| 343,98 |  | 2714,86 |  | 5344,71 |
| 343,88 |  | 2715,72 |  | 5343,96 |
| 343,78 |  | 2716,42 |  | 5343,18 |
| 343,68 |  | 2717,01 |  | 5342,38 |
| 343,58 |  | 2717,58 |  | 5341,57 |
| 343,48 |  | 2718,24 |  | 5340,73 |
| 343,38 |  | 2719,17 |  | 5339,86 |
| 343,28 |  | 2720,48 |  | 5338,96 |
| 343,18 |  | 2722,44 |  | 5338,02 |
| 343,08 |  | 2725,29 |  | 5337,03 |
| 342,98 |  | 2729,3 |  | 5335,98 |
| 342,88 |  | 2734,71 |  | 5334,86 |
| 342,78 |  | 2741,78 |  | 5333,63 |
| 342,68 |  | 2750,74 |  | 5332,29 |
| 342,58 |  | 2761,77 |  | 5330,81 |
| 342,48 |  | 2775,03 |  | 5329,16 |
| 342,38 |  | 2790,6 |  | 5327,32 |
| 342,28 |  | 2808,42 |  | 5325,27 |
| 342,18 |  | 2828,41 |  | 5322,97 |
| 342,08 |  | 2850,33 |  | 5320,4 |
| 341,98 |  | 2873,86 |  | 5317,55 |
| 341,88 |  | 2898,53 |  | 5314,4 |
| 341,78 |  | 2923,78 |  | 5310,94 |
| 341,68 |  | 2948,88 |  | 5307,16 |
| 341,58 |  | 2973,1 |  | 5303,08 |
| 341,48 |  | 2995,67 |  | 5298,69 |
| 341,38 |  | 3015,85 |  | 5294,02 |
| 341,28 |  | 3032,88 |  | 5289,11 |
| 341,18 |  | 3046,14 |  | 5283,97 |
| 341,08 |  | 3055,07 |  | 5278,66 |
| 340,98 |  | 3059,25 |  | 5273,23 |
| 340,88 |  | 3058,44 |  | 5267,72 |
| 340,78 |  | 3052,56 |  | 5262,19 |
| 340,68 |  | 3041,79 |  | 5256,69 |
| 340,58 |  | 3026,53 |  | 5251,29 |
| 340,48 |  | 3007,29 |  | 5246,02 |
| 340,38 |  | 2984,73 |  | 5240,93 |
| 340,28 |  | 2959,57 |  | 5236,07 |
| 340,18 |  | 2932,63 |  | 5231,46 |
| 340,08 |  | 2904,77 |  | 5227,12 |
| 339,98 |  | 2876,85 |  | 5223,08 |
| 339,88 |  | 2849,63 |  | 5219,33 |
| 339,78 |  | 2823,83 |  | 5215,88 |
| 339,68 |  | 2800,04 |  | 5212,71 |
| 339,58 |  | 2778,71 |  | 5209,81 |
| 339,48 |  | 2760,1 |  | 5207,16 |
| 339,38 |  | 2744,37 |  | 5204,72 |
| 339,28 |  | 2731,55 |  | 5202,47 |
| 339,18 |  | 2721,61 |  | 5200,38 |
| 339,08 |  | 2714,39 |  | 5198,42 |
| 338,98 |  | 2709,62 |  | 5196,56 |
| 338,88 |  | 2706,99 |  | 5194,76 |
| 338,78 |  | 2706,19 |  | 5193,01 |
| 338,68 |  | 2706,89 |  | 5191,28 |
| 338,58 |  | 2708,76 |  | 5189,54 |
| 338,48 |  | 2711,5 |  | 5187,79 |
| 338,38 |  | 2714,91 |  | 5186 |
| 338,28 |  | 2718,83 |  | 5184,16 |
| 338,18 |  | 2723,23 |  | 5182,27 |
| 338,08 |  | 2728,03 |  | 5180,3 |
| 337,98 |  | 2733,28 |  | 5178,26 |
| 337,88 |  | 2739,11 |  | 5176,12 |
| 337,78 |  | 2745,72 |  | 5173,88 |
| 337,68 |  | 2753,34 |  | 5171,53 |
| 337,58 |  | 2762,28 |  | 5169,04 |
| 337,48 |  | 2772,89 |  | 5166,39 |
| 337,38 |  | 2785,49 |  | 5163,57 |
| 337,28 |  | 2800,37 |  | 5160,55 |
| 337,18 |  | 2817,74 |  | 5157,3 |
| 337,08 |  | 2837,7 |  | 5153,79 |
| 336,98 |  | 2860,33 |  | 5149,99 |
| 336,88 |  | 2885,57 |  | 5145,88 |
| 336,78 |  | 2913,21 |  | 5141,42 |
| 336,68 |  | 2942,9 |  | 5136,59 |
| 336,58 |  | 2974,06 |  | 5131,38 |
| 336,48 |  | 3005,95 |  | 5125,76 |
| 336,38 |  | 3037,69 |  | 5119,75 |
| 336,28 |  | 3068,31 |  | 5113,34 |
| 336,18 |  | 3096,77 |  | 5106,55 |
| 336,08 |  | 3122,02 |  | 5099,41 |
| 335,98 |  | 3143,04 |  | 5091,96 |
| 335,88 |  | 3158,83 |  | 5084,24 |
| 335,78 |  | 3168,56 |  | 5076,32 |
| 335,68 |  | 3171,57 |  | 5068,26 |
| 335,58 |  | 3167,45 |  | 5060,14 |
| 335,48 |  | 3156,13 |  | 5052,04 |
| 335,38 |  | 3137,76 |  | 5044,03 |
| 335,28 |  | 3112,81 |  | 5036,18 |
| 335,18 |  | 3081,93 |  | 5028,59 |
| 335,08 |  | 3046,02 |  | 5021,3 |
| 334,98 |  | 3006,16 |  | 5014,38 |
| 334,88 |  | 2963,53 |  | 5007,88 |
| 334,78 |  | 2919,35 |  | 5001,83 |
| 334,68 |  | 2874,84 |  | 4996,26 |
| 334,58 |  | 2831,08 |  | 4991,18 |
| 334,48 |  | 2789,02 |  | 4986,6 |
| 334,38 |  | 2749,4 |  | 4982,49 |
| 334,28 |  | 2712,83 |  | 4978,86 |
| 334,18 |  | 2679,65 |  | 4975,66 |
| 334,08 |  | 2650,1 |  | 4972,87 |
| 333,98 |  | 2624,16 |  | 4970,45 |
| 333,88 |  | 2601,68 |  | 4968,37 |
| 333,78 |  | 2582,42 |  | 4966,59 |
| 333,68 |  | 2566,06 |  | 4965,07 |
| 333,58 |  | 2552,27 |  | 4963,78 |
| 333,48 |  | 2540,73 |  | 4962,68 |
| 333,38 |  | 2531,13 |  | 4961,76 |
| 333,28 |  | 2523,19 |  | 4960,97 |
| 333,18 |  | 2516,62 |  | 4960,3 |
| 333,08 |  | 2511,22 |  | 4959,73 |
| 332,98 |  | 2506,79 |  | 4959,24 |
| 332,88 |  | 2503,21 |  | 4958,81 |
| 332,78 |  | 2500,37 |  | 4958,44 |
| 332,68 |  | 2498,17 |  | 4958,11 |
| 332,58 |  | 2496,52 |  | 4957,82 |
| 332,48 |  | 2495,3 |  | 4957,55 |
| 332,38 |  | 2494,39 |  | 4957,31 |
| 332,28 |  | 2493,7 |  | 4957,08 |
| 332,18 |  | 2493,15 |  | 4956,86 |
| 332,08 |  | 2492,7 |  | 4956,65 |
| 331,98 |  | 2492,32 |  | 4956,45 |
| 331,88 |  | 2492,01 |  | 4956,26 |
| 331,78 |  | 2491,72 |  | 4956,07 |
| 331,68 |  | 2491,42 |  | 4955,89 |
| 331,58 |  | 2491,09 |  | 4955,71 |
| 331,48 |  | 2490,76 |  | 4955,53 |
| 331,38 |  | 2490,41 |  | 4955,35 |
| 331,28 |  | 2490,1 |  | 4955,18 |
| 331,18 |  | 2489,82 |  | 4955,01 |
| 331,08 |  | 2489,57 |  | 4954,85 |
| 330,98 |  | 2489,32 |  | 4954,68 |
| 330,88 |  | 2489,05 |  | 4954,52 |
| 330,78 |  | 2488,68 |  | 4954,37 |
| 330,68 |  | 2488,21 |  | 4954,22 |
| 330,58 |  | 2487,64 |  | 4954,08 |
| 330,48 |  | 2486,98 |  | 4953,94 |
| 330,38 |  | 2486,22 |  | 4953,82 |
| 330,28 |  | 2485,38 |  | 4953,7 |
| 330,18 |  | 2484,43 |  | 4953,59 |
| 330,08 |  | 2483,43 |  | 4953,49 |
| 329,98 |  | 2482,37 |  | 4953,41 |
| 329,88 |  | 2481,27 |  | 4953,33 |
| 329,78 |  | 2480,18 |  | 4953,27 |
| 329,68 |  | 2479,16 |  | 4953,22 |
| 329,58 |  | 2478,22 |  | 4953,18 |
| 329,48 |  | 2477,4 |  | 4953,15 |
| 329,38 |  | 2476,69 |  | 4953,12 |
| 329,28 |  | 2476,09 |  | 4953,1 |
| 329,18 |  | 2475,61 |  | 4953,09 |
| 329,08 |  | 2475,23 |  | 4953,08 |

XRD Raw Data

| 3.196289097 96075.0 |
| --- |
| 3.209419380 91448.0 |
| 3.222549663 87837.0 |
| 3.235679946 84677.0 |
| 3.248810228 82480.0 |
| 3.261940511 80335.0 |
| 3.275070794 77756.0 |
| 3.288201076 76102.0 |
| 3.301331359 73700.0 |
| 3.314461642 71383.0 |
| 3.327591924 69423.0 |
| 3.340722207 67823.0 |
| 3.353852490 65757.0 |
| 3.366982772 64017.0 |
| 3.380113055 62102.0 |
| 3.393243338 60288.0 |
| 3.406373620 58739.0 |
| 3.419503903 56784.0 |
| 3.432634186 55277.0 |
| 3.445764468 53537.0 |
| 3.458894751 51838.0 |
| 3.472025034 50444.0 |
| 3.485155316 48511.0 |
| 3.498285599 47427.0 |
| 3.511415882 45882.0 |
| 3.524546164 44092.0 |
| 3.537676447 42639.0 |
| 3.550806730 41127.0 |
| 3.563937012 40074.0 |
| 3.577067295 38689.0 |
| 3.590197578 37357.0 |
| 3.603327860 36008.0 |
| 3.616458143 34358.0 |
| 3.629588426 33327.0 |
| 3.642718708 32138.0 |
| 3.655848991 31045.0 |
| 3.668979274 29845.0 |
| 3.682109557 28345.0 |
| 3.695239839 27081.0 |
| 3.708370122 26308.0 |
| 3.721500405 25432.0 |
| 3.734630687 24136.0 |
| 3.747760970 23054.0 |
| 3.760891253 21830.0 |
| 3.774021535 21024.0 |
| 3.787151818 20325.0 |
| 3.800282101 19144.0 |
| 3.813412383 18181.0 |
| 3.826542666 17347.0 |
| 3.839672949 16459.0 |
| 3.852803231 15529.0 |
| 3.865933514 14595.0 |
| 3.879063797 13901.0 |
| 3.892194079 12982.0 |
| 3.905324362 12224.0 |
| 3.918454645 11602.0 |
| 3.931584927 10858.0 |
| 3.944715210 10357.0 |
| 3.957845493 9807.0 |
| 3.970975775 9602.0 |
| 3.984106058 9396.0 |
| 3.997236341 9102.0 |
| 4.010366623 8923.0 |
| 4.023496906 8848.0 |
| 4.036627189 8724.0 |
| 4.049757471 8731.0 |
| 4.062887754 8448.0 |
| 4.076018037 8414.0 |
| 4.089148319 8233.0 |
| 4.102278602 8218.0 |
| 4.115408885 8095.0 |
| 4.128539168 8161.0 |
| 4.141669450 7903.0 |
| 4.154799733 7975.0 |
| 4.167930016 7751.0 |
| 4.181060298 7683.0 |
| 4.194190581 7555.0 |
| 4.207320864 7560.0 |
| 4.220451146 7496.0 |
| 4.233581429 7442.0 |
| 4.246711712 7542.0 |
| 4.259841994 7228.0 |
| 4.272972277 7307.0 |
| 4.286102560 7170.0 |
| 4.299232842 7148.0 |
| 4.312363125 6902.0 |
| 4.325493408 6954.0 |
| 4.338623690 6981.0 |
| 4.351753973 6815.0 |
| 4.364884256 6684.0 |
| 4.378014538 6646.0 |
| 4.391144821 6779.0 |
| 4.404275104 6618.0 |
| 4.417405386 6482.0 |
| 4.430535669 6511.0 |
| 4.443665952 6426.0 |
| 4.456796234 6360.0 |
| 4.469926517 6444.0 |
| 4.483056800 6375.0 |
| 4.496187082 6266.0 |
| 4.509317365 6249.0 |
| 4.522447648 6371.0 |
| 4.535577930 6191.0 |
| 4.548708213 6073.0 |
| 4.561838496 6177.0 |
| 4.574968779 5995.0 |
| 4.588099061 6035.0 |
| 4.601229344 6050.0 |
| 4.614359627 6141.0 |
| 4.627489909 6039.0 |
| 4.640620192 5929.0 |
| 4.653750475 5971.0 |
| 4.666880757 5918.0 |
| 4.680011040 5957.0 |
| 4.693141323 5835.0 |
| 4.706271605 5909.0 |
| 4.719401888 5868.0 |
| 4.732532171 5806.0 |
| 4.745662453 5702.0 |
| 4.758792736 5827.0 |
| 4.771923019 5705.0 |
| 4.785053301 5725.0 |
| 4.798183584 5651.0 |
| 4.811313867 5616.0 |
| 4.824444149 5625.0 |
| 4.837574432 5492.0 |
| 4.850704715 5376.0 |
| 4.863834997 5623.0 |
| 4.876965280 5589.0 |
| 4.890095563 5456.0 |
| 4.903225845 5443.0 |
| 4.916356128 5455.0 |
| 4.929486411 5402.0 |
| 4.942616693 5585.0 |
| 4.955746976 5297.0 |
| 4.968877259 5342.0 |
| 4.982007541 5258.0 |
| 4.995137824 5339.0 |
| 5.008268107 5359.0 |
| 5.021398390 5227.0 |
| 5.034528672 5367.0 |
| 5.047658955 5181.0 |
| 5.060789238 5190.0 |
| 5.073919520 5062.0 |
| 5.087049803 5027.0 |
| 5.100180086 5130.0 |
| 5.113310368 5127.0 |
| 5.126440651 5219.0 |
| 5.139570934 5052.0 |
| 5.152701216 5019.0 |
| 5.165831499 4995.0 |
| 5.178961782 5055.0 |
| 5.192092064 4987.0 |
| 5.205222347 5078.0 |
| 5.218352630 5067.0 |
| 5.231482912 4934.0 |
| 5.244613195 4860.0 |
| 5.257743478 4989.0 |
| 5.270873760 4968.0 |
| 5.284004043 4949.0 |
| 5.297134326 5048.0 |
| 5.310264608 4877.0 |
| 5.323394891 4845.0 |
| 5.336525174 4817.0 |
| 5.349655456 4795.0 |
| 5.362785739 4807.0 |
| 5.375916022 4798.0 |
| 5.389046304 4769.0 |
| 5.402176587 4884.0 |
| 5.415306870 4723.0 |
| 5.428437153 4736.0 |
| 5.441567435 4701.0 |
| 5.454697718 4730.0 |
| 5.467828001 4654.0 |
| 5.480958283 4694.0 |
| 5.494088566 4684.0 |
| 5.507218849 4580.0 |
| 5.520349131 4637.0 |
| 5.533479414 4786.0 |
| 5.546609697 4633.0 |
| 5.559739979 4579.0 |
| 5.572870262 4614.0 |
| 5.586000545 4551.0 |
| 5.599130827 4564.0 |
| 5.612261110 4566.0 |
| 5.625391393 4625.0 |
| 5.638521675 4613.0 |
| 5.651651958 4584.0 |
| 5.664782241 4578.0 |
| 5.677912523 4505.0 |
| 5.691042806 4524.0 |
| 5.704173089 4420.0 |
| 5.717303371 4593.0 |
| 5.730433654 4471.0 |
| 5.743563937 4429.0 |
| 5.756694219 4456.0 |
| 5.769824502 4370.0 |
| 5.782954785 4527.0 |
| 5.796085067 4439.0 |
| 5.809215350 4490.0 |
| 5.822345633 4374.0 |
| 5.835475915 4300.0 |
| 5.848606198 4286.0 |
| 5.861736481 4227.0 |
| 5.874866764 4268.0 |
| 5.887997046 4324.0 |
| 5.901127329 4304.0 |
| 5.914257612 4337.0 |
| 5.927387894 4232.0 |
| 5.940518177 4407.0 |
| 5.953648460 4197.0 |
| 5.966778742 4099.0 |
| 5.979909025 4219.0 |
| 5.993039308 4288.0 |
| 6.006169590 4310.0 |
| 6.019299873 4255.0 |
| 6.032430156 4243.0 |
| 6.045560438 4206.0 |
| 6.058690721 4056.0 |
| 6.071821004 4230.0 |
| 6.084951286 4128.0 |
| 6.098081569 4264.0 |
| 6.111211852 4268.0 |
| 6.124342134 4163.0 |
| 6.137472417 4194.0 |
| 6.150602700 4146.0 |
| 6.163732982 4217.0 |
| 6.176863265 4144.0 |
| 6.189993548 4210.0 |
| 6.203123830 4228.0 |
| 6.216254113 4231.0 |
| 6.229384396 4119.0 |
| 6.242514678 4269.0 |
| 6.255644961 4159.0 |
| 6.268775244 4034.0 |
| 6.281905526 4141.0 |
| 6.295035809 4077.0 |
| 6.308166092 4088.0 |
| 6.321296375 4044.0 |
| 6.334426657 4000.0 |
| 6.347556940 4000.0 |
| 6.360687223 4017.0 |
| 6.373817505 3997.0 |
| 6.386947788 4003.0 |
| 6.400078071 4008.0 |
| 6.413208353 4077.0 |
| 6.426338636 3995.0 |
| 6.439468919 4010.0 |
| 6.452599201 4052.0 |
| 6.465729484 4037.0 |
| 6.478859767 4017.0 |
| 6.491990049 3945.0 |
| 6.505120332 3967.0 |
| 6.518250615 4084.0 |
| 6.531380897 4092.0 |
| 6.544511180 3979.0 |
| 6.557641463 3945.0 |
| 6.570771745 3950.0 |
| 6.583902028 3944.0 |
| 6.597032311 3935.0 |
| 6.610162593 3932.0 |
| 6.623292876 3963.0 |
| 6.636423159 4052.0 |
| 6.649553441 3907.0 |
| 6.662683724 4045.0 |
| 6.675814007 3991.0 |
| 6.688944289 3987.0 |
| 6.702074572 3856.0 |
| 6.715204855 3921.0 |
| 6.728335137 3833.0 |
| 6.741465420 3927.0 |
| 6.754595703 3821.0 |
| 6.767725986 4048.0 |
| 6.780856268 3918.0 |
| 6.793986551 3936.0 |
| 6.807116834 3847.0 |
| 6.820247116 3883.0 |
| 6.833377399 3969.0 |
| 6.846507682 3874.0 |
| 6.859637964 3964.0 |
| 6.872768247 3875.0 |
| 6.885898530 3872.0 |
| 6.899028812 3918.0 |
| 6.912159095 3975.0 |
| 6.925289378 3921.0 |
| 6.938419660 3872.0 |
| 6.951549943 3852.0 |
| 6.964680226 3782.0 |
| 6.977810508 3863.0 |
| 6.990940791 3805.0 |
| 7.004071074 3786.0 |
| 7.017201356 3873.0 |
| 7.030331639 3696.0 |
| 7.043461922 3804.0 |
| 7.056592204 3820.0 |
| 7.069722487 3833.0 |
| 7.082852770 3796.0 |
| 7.095983052 3754.0 |
| 7.109113335 3881.0 |
| 7.122243618 3728.0 |
| 7.135373900 3651.0 |
| 7.148504183 3723.0 |
| 7.161634466 3705.0 |
| 7.174764748 3689.0 |
| 7.187895031 3751.0 |
| 7.201025314 3786.0 |
| 7.214155597 3820.0 |
| 7.227285879 3816.0 |
| 7.240416162 3856.0 |
| 7.253546445 3729.0 |
| 7.266676727 3667.0 |
| 7.279807010 3745.0 |
| 7.292937293 3793.0 |
| 7.306067575 3722.0 |
| 7.319197858 3711.0 |
| 7.332328141 3735.0 |
| 7.345458423 3841.0 |
| 7.358588706 3818.0 |
| 7.371718989 3787.0 |
| 7.384849271 3855.0 |
| 7.397979554 3658.0 |
| 7.411109837 3784.0 |
| 7.424240119 3686.0 |
| 7.437370402 3851.0 |
| 7.450500685 3595.0 |
| 7.463630967 3703.0 |
| 7.476761250 3701.0 |
| 7.489891533 3725.0 |
| 7.503021815 3696.0 |
| 7.516152098 3803.0 |
| 7.529282381 3745.0 |
| 7.542412663 3737.0 |
| 7.555542946 3696.0 |
| 7.568673229 3712.0 |
| 7.581803511 3710.0 |
| 7.594933794 3583.0 |
| 7.608064077 3615.0 |
| 7.621194359 3745.0 |
| 7.634324642 3642.0 |
| 7.647454925 3623.0 |
| 7.660585208 3758.0 |
| 7.673715490 3706.0 |
| 7.686845773 3756.0 |
| 7.699976056 3638.0 |
| 7.713106338 3670.0 |
| 7.726236621 3606.0 |
| 7.739366904 3669.0 |
| 7.752497186 3666.0 |
| 7.765627469 3599.0 |
| 7.778757752 3609.0 |
| 7.791888034 3665.0 |
| 7.805018317 3637.0 |
| 7.818148600 3615.0 |
| 7.831278882 3729.0 |
| 7.844409165 3622.0 |
| 7.857539448 3630.0 |
| 7.870669730 3639.0 |
| 7.883800013 3659.0 |
| 7.896930296 3639.0 |
| 7.910060578 3637.0 |
| 7.923190861 3549.0 |
| 7.936321144 3576.0 |
| 7.949451426 3637.0 |
| 7.962581709 3564.0 |
| 7.975711992 3666.0 |
| 7.988842274 3700.0 |
| 8.001972557 3745.0 |
| 8.015102840 3548.0 |
| 8.028233122 3582.0 |
| 8.041363405 3584.0 |
| 8.054493688 3585.0 |
| 8.067623971 3601.0 |
| 8.080754253 3615.0 |
| 8.093884536 3602.0 |
| 8.107014819 3610.0 |
| 8.120145101 3582.0 |
| 8.133275384 3518.0 |
| 8.146405667 3628.0 |
| 8.159535949 3572.0 |
| 8.172666232 3567.0 |
| 8.185796515 3646.0 |
| 8.198926797 3496.0 |
| 8.212057080 3594.0 |
| 8.225187363 3635.0 |
| 8.238317645 3482.0 |
| 8.251447928 3556.0 |
| 8.264578211 3545.0 |
| 8.277708493 3626.0 |
| 8.290838776 3540.0 |
| 8.303969059 3538.0 |
| 8.317099341 3522.0 |
| 8.330229624 3554.0 |
| 8.343359907 3482.0 |
| 8.356490189 3620.0 |
| 8.369620472 3703.0 |
| 8.382750755 3614.0 |
| 8.395881037 3614.0 |
| 8.409011320 3533.0 |
| 8.422141603 3537.0 |
| 8.435271885 3502.0 |
| 8.448402168 3515.0 |
| 8.461532451 3578.0 |
| 8.474662733 3562.0 |
| 8.487793016 3585.0 |
| 8.500923299 3557.0 |
| 8.514053582 3500.0 |
| 8.527183864 3586.0 |
| 8.540314147 3559.0 |
| 8.553444430 3545.0 |
| 8.566574712 3558.0 |
| 8.579704995 3445.0 |
| 8.592835278 3506.0 |
| 8.605965560 3506.0 |
| 8.619095843 3467.0 |
| 8.632226126 3465.0 |
| 8.645356408 3506.0 |
| 8.658486691 3575.0 |
| 8.671616974 3419.0 |
| 8.684747256 3482.0 |
| 8.697877539 3595.0 |
| 8.711007822 3570.0 |
| 8.724138104 3577.0 |
| 8.737268387 3625.0 |
| 8.750398670 3441.0 |
| 8.763528952 3425.0 |
| 8.776659235 3517.0 |
| 8.789789518 3508.0 |
| 8.802919800 3542.0 |
| 8.816050083 3461.0 |
| 8.829180366 3451.0 |
| 8.842310648 3579.0 |
| 8.855440931 3582.0 |
| 8.868571214 3412.0 |
| 8.881701496 3423.0 |
| 8.894831779 3437.0 |
| 8.907962062 3465.0 |
| 8.921092344 3519.0 |
| 8.934222627 3523.0 |
| 8.947352910 3452.0 |
| 8.960483193 3459.0 |
| 8.973613475 3371.0 |
| 8.986743758 3421.0 |
| 8.999874041 3488.0 |
| 9.013004323 3505.0 |
| 9.026134606 3531.0 |
| 9.039264889 3381.0 |
| 9.052395171 3551.0 |
| 9.065525454 3568.0 |
| 9.078655737 3452.0 |
| 9.091786019 3598.0 |
| 9.104916302 3464.0 |
| 9.118046585 3536.0 |
| 9.131176867 3509.0 |
| 9.144307150 3412.0 |
| 9.157437433 3415.0 |
| 9.170567715 3517.0 |
| 9.183697998 3409.0 |
| 9.196828281 3494.0 |
| 9.209958563 3604.0 |
| 9.223088846 3598.0 |
| 9.236219129 3424.0 |
| 9.249349411 3544.0 |
| 9.262479694 3552.0 |
| 9.275609977 3545.0 |
| 9.288740259 3490.0 |
| 9.301870542 3477.0 |
| 9.315000825 3359.0 |
| 9.328131107 3495.0 |
| 9.341261390 3466.0 |
| 9.354391673 3384.0 |
| 9.367521955 3538.0 |
| 9.380652238 3464.0 |
| 9.393782521 3335.0 |
| 9.406912804 3520.0 |
| 9.420043086 3407.0 |
| 9.433173369 3468.0 |
| 9.446303652 3478.0 |
| 9.459433934 3569.0 |
| 9.472564217 3352.0 |
| 9.485694500 3315.0 |
| 9.498824782 3363.0 |
| 9.511955065 3443.0 |
| 9.525085348 3477.0 |
| 9.538215630 3473.0 |
| 9.551345913 3418.0 |
| 9.564476196 3391.0 |
| 9.577606478 3415.0 |
| 9.590736761 3428.0 |
| 9.603867044 3406.0 |
| 9.616997326 3469.0 |
| 9.630127609 3470.0 |
| 9.643257892 3369.0 |
| 9.656388174 3409.0 |
| 9.669518457 3408.0 |
| 9.682648740 3361.0 |
| 9.695779022 3437.0 |
| 9.708909305 3350.0 |
| 9.722039588 3381.0 |
| 9.735169870 3427.0 |
| 9.748300153 3373.0 |
| 9.761430436 3424.0 |
| 9.774560718 3319.0 |
| 9.787691001 3486.0 |
| 9.800821284 3381.0 |
| 9.813951566 3358.0 |
| 9.827081849 3390.0 |
| 9.840212132 3376.0 |
| 9.853342415 3441.0 |
| 9.866472697 3422.0 |
| 9.879602980 3381.0 |
| 9.892733263 3335.0 |
| 9.905863545 3438.0 |
| 9.918993828 3393.0 |
| 9.932124111 3345.0 |
| 9.945254393 3302.0 |
| 9.958384676 3380.0 |
| 9.971514959 3358.0 |
| 9.984645241 3391.0 |
| 9.997775524 3359.0 |
| 10.010905807 3336.0 |
| 10.024036089 3351.0 |
| 10.037166372 3528.0 |
| 10.050296655 3497.0 |
| 10.063426937 3360.0 |
| 10.076557220 3258.0 |
| 10.089687503 3381.0 |
| 10.102817785 3356.0 |
| 10.115948068 3278.0 |
| 10.129078351 3291.0 |
| 10.142208633 3425.0 |
| 10.155338916 3404.0 |
| 10.168469199 3432.0 |
| 10.181599481 3284.0 |
| 10.194729764 3439.0 |
| 10.207860047 3361.0 |
| 10.220990329 3340.0 |
| 10.234120612 3429.0 |
| 10.247250895 3438.0 |
| 10.260381178 3349.0 |
| 10.273511460 3390.0 |
| 10.286641743 3262.0 |
| 10.299772026 3341.0 |
| 10.312902308 3391.0 |
| 10.326032591 3475.0 |
| 10.339162874 3485.0 |
| 10.352293156 3455.0 |
| 10.365423439 3386.0 |
| 10.378553722 3277.0 |
| 10.391684004 3431.0 |
| 10.404814287 3394.0 |
| 10.417944570 3346.0 |
| 10.431074852 3440.0 |
| 10.444205135 3425.0 |
| 10.457335418 3333.0 |
| 10.470465700 3340.0 |
| 10.483595983 3358.0 |
| 10.496726266 3330.0 |
| 10.509856548 3340.0 |
| 10.522986831 3343.0 |
| 10.536117114 3417.0 |
| 10.549247396 3350.0 |
| 10.562377679 3366.0 |
| 10.575507962 3259.0 |
| 10.588638244 3255.0 |
| 10.601768527 3313.0 |
| 10.614898810 3418.0 |
| 10.628029092 3288.0 |
| 10.641159375 3311.0 |
| 10.654289658 3362.0 |
| 10.667419940 3353.0 |
| 10.680550223 3309.0 |
| 10.693680506 3358.0 |
| 10.706810789 3320.0 |
| 10.719941071 3242.0 |
| 10.733071354 3323.0 |
| 10.746201637 3507.0 |
| 10.759331919 3321.0 |
| 10.772462202 3220.0 |
| 10.785592485 3341.0 |
| 10.798722767 3231.0 |
| 10.811853050 3202.0 |
| 10.824983333 3381.0 |
| 10.838113615 3273.0 |
| 10.851243898 3368.0 |
| 10.864374181 3306.0 |
| 10.877504463 3265.0 |
| 10.890634746 3282.0 |
| 10.903765029 3242.0 |
| 10.916895311 3258.0 |
| 10.930025594 3314.0 |
| 10.943155877 3183.0 |
| 10.956286159 3283.0 |
| 10.969416442 3300.0 |
| 10.982546725 3320.0 |
| 10.995677007 3159.0 |
| 11.008807290 3144.0 |
| 11.021937573 3171.0 |
| 11.035067855 3186.0 |
| 11.048198138 3383.0 |
| 11.061328421 3167.0 |
| 11.074458703 3303.0 |
| 11.087588986 3233.0 |
| 11.100719269 3093.0 |
| 11.113849551 3243.0 |
| 11.126979834 3307.0 |
| 11.140110117 3299.0 |
| 11.153240400 3217.0 |
| 11.166370682 3291.0 |
| 11.179500965 3181.0 |
| 11.192631248 3208.0 |
| 11.205761530 3258.0 |
| 11.218891813 3245.0 |
| 11.232022096 3223.0 |
| 11.245152378 3209.0 |
| 11.258282661 3244.0 |
| 11.271412944 3180.0 |
| 11.284543226 3220.0 |
| 11.297673509 3151.0 |
| 11.310803792 3130.0 |
| 11.323934074 3199.0 |
| 11.337064357 3257.0 |
| 11.350194640 3155.0 |
| 11.363324922 3204.0 |
| 11.376455205 3187.0 |
| 11.389585488 3276.0 |
| 11.402715770 3201.0 |
| 11.415846053 3140.0 |
| 11.428976336 3183.0 |
| 11.442106618 3148.0 |
| 11.455236901 3192.0 |
| 11.468367184 3263.0 |
| 11.481497466 3230.0 |
| 11.494627749 3135.0 |
| 11.507758032 3247.0 |
| 11.520888314 3237.0 |
| 11.534018597 3306.0 |
| 11.547148880 3209.0 |
| 11.560279162 3291.0 |
| 11.573409445 3246.0 |
| 11.586539728 3170.0 |
| 11.599670011 3128.0 |
| 11.612800293 3090.0 |
| 11.625930576 3215.0 |
| 11.639060859 3082.0 |
| 11.652191141 3214.0 |
| 11.665321424 3155.0 |
| 11.678451707 3229.0 |
| 11.691581989 3274.0 |
| 11.704712272 3205.0 |
| 11.717842555 3256.0 |
| 11.730972837 3228.0 |
| 11.744103120 3216.0 |
| 11.757233403 3130.0 |
| 11.770363685 3213.0 |
| 11.783493968 3099.0 |
| 11.796624251 3160.0 |
| 11.809754533 3208.0 |
| 11.822884816 3233.0 |
| 11.836015099 3216.0 |
| 11.849145381 3160.0 |
| 11.862275664 3250.0 |
| 11.875405947 3187.0 |
| 11.888536229 3228.0 |
| 11.901666512 3245.0 |
| 11.914796795 3223.0 |
| 11.927927077 3201.0 |
| 11.941057360 3171.0 |
| 11.954187643 3228.0 |
| 11.967317925 3106.0 |
| 11.980448208 3168.0 |
| 11.993578491 3181.0 |
| 12.006708773 3229.0 |
| 12.019839056 3148.0 |
| 12.032969339 3055.0 |
| 12.046099622 3159.0 |
| 12.059229904 3227.0 |
| 12.072360187 3180.0 |
| 12.085490470 3152.0 |
| 12.098620752 3081.0 |
| 12.111751035 3247.0 |
| 12.124881318 3125.0 |
| 12.138011600 3200.0 |
| 12.151141883 3079.0 |
| 12.164272166 3147.0 |
| 12.177402448 3189.0 |
| 12.190532731 3179.0 |
| 12.203663014 3097.0 |
| 12.216793296 3168.0 |
| 12.229923579 3067.0 |
| 12.243053862 3118.0 |
| 12.256184144 3020.0 |
| 12.269314427 3096.0 |
| 12.282444710 3101.0 |
| 12.295574992 3114.0 |
| 12.308705275 3192.0 |
| 12.321835558 3182.0 |
| 12.334965840 3164.0 |
| 12.348096123 3103.0 |
| 12.361226406 3024.0 |
| 12.374356688 3185.0 |
| 12.387486971 3015.0 |
| 12.400617254 3150.0 |
| 12.413747536 3012.0 |
| 12.426877819 3121.0 |
| 12.440008102 3036.0 |
| 12.453138384 3145.0 |
| 12.466268667 3186.0 |
| 12.479398950 3146.0 |
| 12.492529233 3123.0 |
| 12.505659515 3216.0 |
| 12.518789798 3079.0 |
| 12.531920081 3091.0 |
| 12.545050363 3069.0 |
| 12.558180646 3073.0 |
| 12.571310929 3072.0 |
| 12.584441211 3086.0 |
| 12.597571494 3127.0 |
| 12.610701777 3074.0 |
| 12.623832059 3069.0 |
| 12.636962342 3140.0 |
| 12.650092625 3051.0 |
| 12.663222907 3113.0 |
| 12.676353190 3162.0 |
| 12.689483473 3255.0 |
| 12.702613755 3016.0 |
| 12.715744038 3059.0 |
| 12.728874321 3104.0 |
| 12.742004603 3003.0 |
| 12.755134886 3082.0 |
| 12.768265169 3027.0 |
| 12.781395451 3120.0 |
| 12.794525734 3134.0 |
| 12.807656017 3068.0 |
| 12.820786299 3121.0 |
| 12.833916582 3045.0 |
| 12.847046865 3149.0 |
| 12.860177147 3153.0 |
| 12.873307430 3058.0 |
| 12.886437713 3085.0 |
| 12.899567996 2997.0 |
| 12.912698278 3166.0 |
| 12.925828561 3121.0 |
| 12.938958844 2990.0 |
| 12.952089126 3122.0 |
| 12.965219409 3066.0 |
| 12.978349692 3073.0 |
| 12.991479974 3014.0 |
| 13.004610257 3082.0 |
| 13.017740540 3117.0 |
| 13.030870822 3087.0 |
| 13.044001105 3090.0 |
| 13.057131388 3034.0 |
| 13.070261670 3116.0 |
| 13.083391953 2994.0 |
| 13.096522236 3021.0 |
| 13.109652518 3074.0 |
| 13.122782801 3082.0 |
| 13.135913084 3115.0 |
| 13.149043366 3150.0 |
| 13.162173649 3240.0 |
| 13.175303932 3107.0 |
| 13.188434214 2982.0 |
| 13.201564497 3104.0 |
| 13.214694780 3052.0 |
| 13.227825062 3082.0 |
| 13.240955345 3005.0 |
| 13.254085628 3044.0 |
| 13.267215910 3054.0 |
| 13.280346193 3091.0 |
| 13.293476476 3088.0 |
| 13.306606758 3072.0 |
| 13.319737041 3037.0 |
| 13.332867324 2979.0 |
| 13.345997607 2972.0 |
| 13.359127889 3055.0 |
| 13.372258172 3055.0 |
| 13.385388455 3062.0 |
| 13.398518737 3126.0 |
| 13.411649020 3024.0 |
| 13.424779303 3078.0 |
| 13.437909585 3062.0 |
| 13.451039868 3083.0 |
| 13.464170151 2968.0 |
| 13.477300433 2995.0 |
| 13.490430716 3071.0 |
| 13.503560999 2989.0 |
| 13.516691281 2935.0 |
| 13.529821564 3178.0 |
| 13.542951847 3057.0 |
| 13.556082129 3034.0 |
| 13.569212412 3095.0 |
| 13.582342695 3083.0 |
| 13.595472977 3088.0 |
| 13.608603260 3012.0 |
| 13.621733543 3068.0 |
| 13.634863825 3060.0 |
| 13.647994108 3021.0 |
| 13.661124391 3069.0 |
| 13.674254673 3121.0 |
| 13.687384956 3101.0 |
| 13.700515239 3124.0 |
| 13.713645521 3016.0 |
| 13.726775804 3063.0 |
| 13.739906087 2997.0 |
| 13.753036369 3042.0 |
| 13.766166652 2959.0 |
| 13.779296935 3019.0 |
| 13.792427218 3018.0 |
| 13.805557500 2991.0 |
| 13.818687783 3046.0 |
| 13.831818066 3032.0 |
| 13.844948348 3117.0 |
| 13.858078631 3054.0 |
| 13.871208914 2892.0 |
| 13.884339196 3163.0 |
| 13.897469479 3024.0 |
| 13.910599762 3060.0 |
| 13.923730044 3104.0 |
| 13.936860327 3115.0 |
| 13.949990610 3001.0 |
| 13.963120892 2998.0 |
| 13.976251175 3036.0 |
| 13.989381458 3108.0 |
| 14.002511740 3176.0 |
| 14.015642023 3097.0 |
| 14.028772306 3074.0 |
| 14.041902588 3139.0 |
| 14.055032871 3133.0 |
| 14.068163154 3136.0 |
| 14.081293436 3180.0 |
| 14.094423719 3019.0 |
| 14.107554002 3129.0 |
| 14.120684284 3125.0 |
| 14.133814567 3272.0 |
| 14.146944850 3183.0 |
| 14.160075132 3060.0 |
| 14.173205415 3076.0 |
| 14.186335698 3297.0 |
| 14.199465980 3248.0 |
| 14.212596263 3280.0 |
| 14.225726546 3222.0 |
| 14.238856829 3240.0 |
| 14.251987111 3261.0 |
| 14.265117394 3267.0 |
| 14.278247677 3422.0 |
| 14.291377959 3426.0 |
| 14.304508242 3424.0 |
| 14.317638525 3482.0 |
| 14.330768807 3391.0 |
| 14.343899090 3488.0 |
| 14.357029373 3646.0 |
| 14.370159655 3696.0 |
| 14.383289938 3645.0 |
| 14.396420221 3717.0 |
| 14.409550503 3789.0 |
| 14.422680786 3733.0 |
| 14.435811069 3793.0 |
| 14.448941351 3853.0 |
| 14.462071634 3995.0 |
| 14.475201917 4070.0 |
| 14.488332199 3952.0 |
| 14.501462482 3972.0 |
| 14.514592765 4039.0 |
| 14.527723047 4031.0 |
| 14.540853330 4086.0 |
| 14.553983613 4157.0 |
| 14.567113895 4081.0 |
| 14.580244178 4045.0 |
| 14.593374461 4022.0 |
| 14.606504743 3982.0 |
| 14.619635026 4099.0 |
| 14.632765309 4163.0 |
| 14.645895591 4098.0 |
| 14.659025874 3975.0 |
| 14.672156157 4001.0 |
| 14.685286440 3998.0 |
| 14.698416722 4039.0 |
| 14.711547005 4056.0 |
| 14.724677288 3958.0 |
| 14.737807570 3906.0 |
| 14.750937853 3898.0 |
| 14.764068136 3827.0 |
| 14.777198418 3916.0 |
| 14.790328701 3854.0 |
| 14.803458984 3745.0 |
| 14.816589266 3682.0 |
| 14.829719549 3726.0 |
| 14.842849832 3498.0 |
| 14.855980114 3656.0 |
| 14.869110397 3708.0 |
| 14.882240680 3511.0 |
| 14.895370962 3628.0 |
| 14.908501245 3660.0 |
| 14.921631528 3427.0 |
| 14.934761810 3423.0 |
| 14.947892093 3499.0 |
| 14.961022376 3441.0 |
| 14.974152658 3386.0 |
| 14.987282941 3362.0 |
| 15.000413224 3367.0 |
| 15.013543506 3452.0 |
| 15.026673789 3487.0 |
| 15.039804072 3410.0 |
| 15.052934354 3517.0 |
| 15.066064637 3331.0 |
| 15.079194920 3322.0 |
| 15.092325203 3331.0 |
| 15.105455485 3352.0 |
| 15.118585768 3451.0 |
| 15.131716051 3346.0 |
| 15.144846333 3378.0 |
| 15.157976616 3435.0 |
| 15.171106899 3384.0 |
| 15.184237181 3484.0 |
| 15.197367464 3423.0 |
| 15.210497747 3411.0 |
| 15.223628029 3326.0 |
| 15.236758312 3360.0 |
| 15.249888595 3419.0 |
| 15.263018877 3314.0 |
| 15.276149160 3405.0 |
| 15.289279443 3366.0 |
| 15.302409725 3187.0 |
| 15.315540008 3331.0 |
| 15.328670291 3310.0 |
| 15.341800573 3374.0 |
| 15.354930856 3302.0 |
| 15.368061139 3250.0 |
| 15.381191421 3196.0 |
| 15.394321704 3272.0 |
| 15.407451987 3247.0 |
| 15.420582269 3253.0 |
| 15.433712552 3262.0 |
| 15.446842835 3288.0 |
| 15.459973117 3270.0 |
| 15.473103400 3346.0 |
| 15.486233683 3220.0 |
| 15.499363965 3168.0 |
| 15.512494248 3197.0 |
| 15.525624531 3186.0 |
| 15.538754814 3079.0 |
| 15.551885096 3159.0 |
| 15.565015379 3079.0 |
| 15.578145662 3080.0 |
| 15.591275944 2975.0 |
| 15.604406227 3035.0 |
| 15.617536510 3047.0 |
| 15.630666792 2961.0 |
| 15.643797075 3125.0 |
| 15.656927358 3056.0 |
| 15.670057640 3054.0 |
| 15.683187923 2908.0 |
| 15.696318206 2929.0 |
| 15.709448488 2975.0 |
| 15.722578771 3073.0 |
| 15.735709054 2978.0 |
| 15.748839336 2968.0 |
| 15.761969619 2979.0 |
| 15.775099902 2996.0 |
| 15.788230184 2968.0 |
| 15.801360467 2962.0 |
| 15.814490750 3006.0 |
| 15.827621032 2944.0 |
| 15.840751315 2991.0 |
| 15.853881598 2981.0 |
| 15.867011880 3067.0 |
| 15.880142163 3016.0 |
| 15.893272446 3029.0 |
| 15.906402728 3006.0 |
| 15.919533011 2974.0 |
| 15.932663294 2945.0 |
| 15.945793576 3068.0 |
| 15.958923859 2946.0 |
| 15.972054142 3038.0 |
| 15.985184425 3054.0 |
| 15.998314707 3064.0 |
| 16.011444990 3018.0 |
| 16.024575273 3080.0 |
| 16.037705555 3082.0 |
| 16.050835838 3048.0 |
| 16.063966121 3023.0 |
| 16.077096403 3085.0 |
| 16.090226686 2910.0 |
| 16.103356969 3102.0 |
| 16.116487251 3117.0 |
| 16.129617534 3140.0 |
| 16.142747817 2998.0 |
| 16.155878099 3066.0 |
| 16.169008382 3063.0 |
| 16.182138665 3190.0 |
| 16.195268947 3142.0 |
| 16.208399230 3057.0 |
| 16.221529513 3138.0 |
| 16.234659795 3083.0 |
| 16.247790078 3116.0 |
| 16.260920361 3084.0 |
| 16.274050643 3129.0 |
| 16.287180926 3026.0 |
| 16.300311209 3044.0 |
| 16.313441491 3005.0 |
| 16.326571774 3047.0 |
| 16.339702057 3085.0 |
| 16.352832339 3115.0 |
| 16.365962622 3096.0 |
| 16.379092905 3022.0 |
| 16.392223187 3013.0 |
| 16.405353470 2956.0 |
| 16.418483753 3022.0 |
| 16.431614036 2999.0 |
| 16.444744318 3064.0 |
| 16.457874601 3053.0 |
| 16.471004884 2998.0 |
| 16.484135166 2949.0 |
| 16.497265449 2952.0 |
| 16.510395732 2944.0 |
| 16.523526014 2980.0 |
| 16.536656297 3000.0 |
| 16.549786580 3004.0 |
| 16.562916862 3027.0 |
| 16.576047145 2999.0 |
| 16.589177428 2842.0 |
| 16.602307710 2909.0 |
| 16.615437993 2837.0 |
| 16.628568276 2943.0 |
| 16.641698558 2888.0 |
| 16.654828841 2886.0 |
| 16.667959124 2882.0 |
| 16.681089406 2750.0 |
| 16.694219689 2835.0 |
| 16.707349972 2831.0 |
| 16.720480254 2929.0 |
| 16.733610537 2847.0 |
| 16.746740820 2821.0 |
| 16.759871102 2837.0 |
| 16.773001385 2874.0 |
| 16.786131668 2808.0 |
| 16.799261950 2787.0 |
| 16.812392233 2789.0 |
| 16.825522516 2936.0 |
| 16.838652798 2810.0 |
| 16.851783081 2831.0 |
| 16.864913364 2820.0 |
| 16.878043647 2871.0 |
| 16.891173929 2794.0 |
| 16.904304212 2755.0 |
| 16.917434495 2869.0 |
| 16.930564777 2722.0 |
| 16.943695060 2791.0 |
| 16.956825343 2763.0 |
| 16.969955625 2798.0 |
| 16.983085908 2711.0 |
| 16.996216191 2754.0 |
| 17.009346473 2877.0 |
| 17.022476756 2853.0 |
| 17.035607039 2846.0 |
| 17.048737321 2802.0 |
| 17.061867604 2778.0 |
| 17.074997887 2783.0 |
| 17.088128169 2906.0 |
| 17.101258452 2838.0 |
| 17.114388735 2847.0 |
| 17.127519017 2848.0 |
| 17.140649300 2712.0 |
| 17.153779583 2800.0 |
| 17.166909865 2851.0 |
| 17.180040148 2843.0 |
| 17.193170431 2759.0 |
| 17.206300713 2897.0 |
| 17.219430996 2839.0 |
| 17.232561279 2917.0 |
| 17.245691561 2734.0 |
| 17.258821844 2798.0 |
| 17.271952127 2795.0 |
| 17.285082409 2803.0 |
| 17.298212692 2744.0 |
| 17.311342975 2734.0 |
| 17.324473258 2829.0 |
| 17.337603540 2922.0 |
| 17.350733823 2899.0 |
| 17.363864106 2762.0 |
| 17.376994388 2738.0 |
| 17.390124671 2753.0 |
| 17.403254954 2778.0 |
| 17.416385236 2721.0 |
| 17.429515519 2772.0 |
| 17.442645802 2818.0 |
| 17.455776084 2886.0 |
| 17.468906367 2783.0 |
| 17.482036650 2737.0 |
| 17.495166932 2927.0 |
| 17.508297215 2879.0 |
| 17.521427498 2831.0 |
| 17.534557780 2816.0 |
| 17.547688063 2892.0 |
| 17.560818346 2916.0 |
| 17.573948628 2847.0 |
| 17.587078911 2770.0 |
| 17.600209194 2899.0 |
| 17.613339476 2966.0 |
| 17.626469759 2877.0 |
| 17.639600042 2854.0 |
| 17.652730324 2867.0 |
| 17.665860607 2842.0 |
| 17.678990890 2887.0 |
| 17.692121172 2878.0 |
| 17.705251455 2853.0 |
| 17.718381738 2958.0 |
| 17.731512021 2994.0 |
| 17.744642303 2945.0 |
| 17.757772586 3013.0 |
| 17.770902869 2955.0 |
| 17.784033151 2899.0 |
| 17.797163434 2900.0 |
| 17.810293717 3019.0 |
| 17.823423999 2906.0 |
| 17.836554282 3040.0 |
| 17.849684565 3018.0 |
| 17.862814847 3046.0 |
| 17.875945130 3036.0 |
| 17.889075413 2978.0 |
| 17.902205695 3101.0 |
| 17.915335978 3107.0 |
| 17.928466261 2949.0 |
| 17.941596543 3059.0 |
| 17.954726826 3106.0 |
| 17.967857109 3063.0 |
| 17.980987391 3029.0 |
| 17.994117674 3095.0 |
| 18.007247957 3067.0 |
| 18.020378239 2996.0 |
| 18.033508522 3048.0 |
| 18.046638805 3134.0 |
| 18.059769087 3071.0 |
| 18.072899370 3072.0 |
| 18.086029653 2920.0 |
| 18.099159935 2923.0 |
| 18.112290218 2964.0 |
| 18.125420501 2986.0 |
| 18.138550783 2942.0 |
| 18.151681066 3047.0 |
| 18.164811349 2979.0 |
| 18.177941632 2949.0 |
| 18.191071914 2924.0 |
| 18.204202197 2843.0 |
| 18.217332480 2898.0 |
| 18.230462762 2820.0 |
| 18.243593045 2794.0 |
| 18.256723328 2873.0 |
| 18.269853610 2907.0 |
| 18.282983893 2866.0 |
| 18.296114176 2740.0 |
| 18.309244458 2830.0 |
| 18.322374741 2810.0 |
| 18.335505024 2865.0 |
| 18.348635306 2895.0 |
| 18.361765589 2745.0 |
| 18.374895872 2722.0 |
| 18.388026154 2798.0 |
| 18.401156437 2748.0 |
| 18.414286720 2917.0 |
| 18.427417002 2736.0 |
| 18.440547285 2689.0 |
| 18.453677568 2670.0 |
| 18.466807850 2782.0 |
| 18.479938133 2703.0 |
| 18.493068416 2630.0 |
| 18.506198698 2796.0 |
| 18.519328981 2679.0 |
| 18.532459264 2655.0 |
| 18.545589546 2796.0 |
| 18.558719829 2684.0 |
| 18.571850112 2647.0 |
| 18.584980394 2675.0 |
| 18.598110677 2638.0 |
| 18.611240960 2659.0 |
| 18.624371243 2696.0 |
| 18.637501525 2805.0 |
| 18.650631808 2691.0 |
| 18.663762091 2734.0 |
| 18.676892373 2657.0 |
| 18.690022656 2575.0 |
| 18.703152939 2613.0 |
| 18.716283221 2713.0 |
| 18.729413504 2671.0 |
| 18.742543787 2726.0 |
| 18.755674069 2605.0 |
| 18.768804352 2680.0 |
| 18.781934635 2695.0 |
| 18.795064917 2684.0 |
| 18.808195200 2686.0 |
| 18.821325483 2690.0 |
| 18.834455765 2658.0 |
| 18.847586048 2699.0 |
| 18.860716331 2612.0 |
| 18.873846613 2670.0 |
| 18.886976896 2666.0 |
| 18.900107179 2685.0 |
| 18.913237461 2698.0 |
| 18.926367744 2613.0 |
| 18.939498027 2704.0 |
| 18.952628309 2697.0 |
| 18.965758592 2644.0 |
| 18.978888875 2673.0 |
| 18.992019157 2531.0 |
| 19.005149440 2665.0 |
| 19.018279723 2682.0 |
| 19.031410005 2673.0 |
| 19.044540288 2668.0 |
| 19.057670571 2647.0 |
| 19.070800854 2647.0 |
| 19.083931136 2626.0 |
| 19.097061419 2635.0 |
| 19.110191702 2654.0 |
| 19.123321984 2612.0 |
| 19.136452267 2726.0 |
| 19.149582550 2723.0 |
| 19.162712832 2583.0 |
| 19.175843115 2610.0 |
| 19.188973398 2704.0 |
| 19.202103680 2674.0 |
| 19.215233963 2516.0 |
| 19.228364246 2569.0 |
| 19.241494528 2634.0 |
| 19.254624811 2576.0 |
| 19.267755094 2593.0 |
| 19.280885376 2597.0 |
| 19.294015659 2582.0 |
| 19.307145942 2565.0 |
| 19.320276224 2742.0 |
| 19.333406507 2778.0 |
| 19.346536790 2625.0 |
| 19.359667072 2629.0 |
| 19.372797355 2671.0 |
| 19.385927638 2690.0 |
| 19.399057920 2538.0 |
| 19.412188203 2603.0 |
| 19.425318486 2569.0 |
| 19.438448768 2661.0 |
| 19.451579051 2656.0 |
| 19.464709334 2578.0 |
| 19.477839616 2648.0 |
| 19.490969899 2632.0 |
| 19.504100182 2612.0 |
| 19.517230465 2627.0 |
| 19.530360747 2638.0 |
| 19.543491030 2533.0 |
| 19.556621313 2558.0 |
| 19.569751595 2600.0 |
| 19.582881878 2680.0 |
| 19.596012161 2658.0 |
| 19.609142443 2660.0 |
| 19.622272726 2597.0 |
| 19.635403009 2651.0 |
| 19.648533291 2641.0 |
| 19.661663574 2598.0 |
| 19.674793857 2611.0 |
| 19.687924139 2557.0 |
| 19.701054422 2710.0 |
| 19.714184705 2720.0 |
| 19.727314987 2647.0 |
| 19.740445270 2627.0 |
| 19.753575553 2566.0 |
| 19.766705835 2509.0 |
| 19.779836118 2607.0 |
| 19.792966401 2583.0 |
| 19.806096683 2640.0 |
| 19.819226966 2660.0 |
| 19.832357249 2641.0 |
| 19.845487531 2593.0 |
| 19.858617814 2624.0 |
| 19.871748097 2647.0 |
| 19.884878379 2499.0 |
| 19.898008662 2656.0 |
| 19.911138945 2563.0 |
| 19.924269228 2619.0 |
| 19.937399510 2627.0 |
| 19.950529793 2573.0 |
| 19.963660076 2622.0 |
| 19.976790358 2566.0 |
| 19.989920641 2618.0 |
| 20.003050924 2645.0 |
| 20.016181206 2541.0 |
| 20.029311489 2575.0 |
| 20.042441772 2567.0 |
| 20.055572054 2577.0 |
| 20.068702337 2551.0 |
| 20.081832620 2583.0 |
| 20.094962902 2562.0 |
| 20.108093185 2637.0 |
| 20.121223468 2640.0 |
| 20.134353750 2579.0 |
| 20.147484033 2633.0 |
| 20.160614316 2540.0 |
| 20.173744598 2562.0 |
| 20.186874881 2543.0 |
| 20.200005164 2573.0 |
| 20.213135446 2638.0 |
| 20.226265729 2601.0 |
| 20.239396012 2611.0 |
| 20.252526294 2587.0 |
| 20.265656577 2558.0 |
| 20.278786860 2667.0 |
| 20.291917142 2595.0 |
| 20.305047425 2603.0 |
| 20.318177708 2576.0 |
| 20.331307990 2602.0 |
| 20.344438273 2526.0 |
| 20.357568556 2494.0 |
| 20.370698839 2618.0 |
| 20.383829121 2589.0 |
| 20.396959404 2600.0 |
| 20.410089687 2654.0 |
| 20.423219969 2529.0 |
| 20.436350252 2566.0 |
| 20.449480535 2623.0 |
| 20.462610817 2687.0 |
| 20.475741100 2614.0 |
| 20.488871383 2627.0 |
| 20.502001665 2488.0 |
| 20.515131948 2582.0 |
| 20.528262231 2628.0 |
| 20.541392513 2662.0 |
| 20.554522796 2607.0 |
| 20.567653079 2593.0 |
| 20.580783361 2573.0 |
| 20.593913644 2646.0 |
| 20.607043927 2581.0 |
| 20.620174209 2640.0 |
| 20.633304492 2504.0 |
| 20.646434775 2499.0 |
| 20.659565057 2577.0 |
| 20.672695340 2610.0 |
| 20.685825623 2658.0 |
| 20.698955905 2682.0 |
| 20.712086188 2581.0 |
| 20.725216471 2604.0 |
| 20.738346753 2638.0 |
| 20.751477036 2695.0 |
| 20.764607319 2635.0 |
| 20.777737601 2587.0 |
| 20.790867884 2518.0 |
| 20.803998167 2658.0 |
| 20.817128450 2682.0 |
| 20.830258732 2661.0 |
| 20.843389015 2607.0 |
| 20.856519298 2592.0 |
| 20.869649580 2538.0 |
| 20.882779863 2589.0 |
| 20.895910146 2612.0 |
| 20.909040428 2685.0 |
| 20.922170711 2686.0 |
| 20.935300994 2612.0 |
| 20.948431276 2522.0 |
| 20.961561559 2642.0 |
| 20.974691842 2763.0 |
| 20.987822124 2555.0 |
| 21.000952407 2782.0 |
| 21.014082690 2690.0 |
| 21.027212972 2599.0 |
| 21.040343255 2661.0 |
| 21.053473538 2617.0 |
| 21.066603820 2714.0 |
| 21.079734103 2632.0 |
| 21.092864386 2729.0 |
| 21.105994668 2709.0 |
| 21.119124951 2733.0 |
| 21.132255234 2720.0 |
| 21.145385516 2801.0 |
| 21.158515799 2742.0 |
| 21.171646082 2654.0 |
| 21.184776364 2623.0 |
| 21.197906647 2732.0 |
| 21.211036930 2827.0 |
| 21.224167212 2802.0 |
| 21.237297495 2647.0 |
| 21.250427778 2746.0 |
| 21.263558061 2703.0 |
| 21.276688343 2655.0 |
| 21.289818626 2688.0 |
| 21.302948909 2666.0 |
| 21.316079191 2703.0 |
| 21.329209474 2667.0 |
| 21.342339757 2692.0 |
| 21.355470039 2745.0 |
| 21.368600322 2593.0 |
| 21.381730605 2577.0 |
| 21.394860887 2696.0 |
| 21.407991170 2611.0 |
| 21.421121453 2640.0 |
| 21.434251735 2671.0 |
| 21.447382018 2690.0 |
| 21.460512301 2667.0 |
| 21.473642583 2640.0 |
| 21.486772866 2674.0 |
| 21.499903149 2611.0 |
| 21.513033431 2593.0 |
| 21.526163714 2627.0 |
| 21.539293997 2578.0 |
| 21.552424279 2607.0 |
| 21.565554562 2591.0 |
| 21.578684845 2684.0 |
| 21.591815127 2640.0 |
| 21.604945410 2690.0 |
| 21.618075693 2689.0 |
| 21.631205975 2604.0 |
| 21.644336258 2536.0 |
| 21.657466541 2547.0 |
| 21.670596823 2565.0 |
| 21.683727106 2740.0 |
| 21.696857389 2725.0 |
| 21.709987672 2638.0 |
| 21.723117954 2627.0 |
| 21.736248237 2642.0 |
| 21.749378520 2624.0 |
| 21.762508802 2648.0 |
| 21.775639085 2586.0 |
| 21.788769368 2675.0 |
| 21.801899650 2538.0 |
| 21.815029933 2639.0 |
| 21.828160216 2615.0 |
| 21.841290498 2589.0 |
| 21.854420781 2683.0 |
| 21.867551064 2682.0 |
| 21.880681346 2654.0 |
| 21.893811629 2609.0 |
| 21.906941912 2665.0 |
| 21.920072194 2744.0 |
| 21.933202477 2660.0 |
| 21.946332760 2590.0 |
| 21.959463042 2605.0 |
| 21.972593325 2627.0 |
| 21.985723608 2685.0 |
| 21.998853890 2565.0 |
| 22.011984173 2535.0 |
| 22.025114456 2569.0 |
| 22.038244738 2632.0 |
| 22.051375021 2580.0 |
| 22.064505304 2606.0 |
| 22.077635586 2594.0 |
| 22.090765869 2536.0 |
| 22.103896152 2656.0 |
| 22.117026434 2670.0 |
| 22.130156717 2581.0 |
| 22.143287000 2527.0 |
| 22.156417283 2710.0 |
| 22.169547565 2578.0 |
| 22.182677848 2605.0 |
| 22.195808131 2598.0 |
| 22.208938413 2714.0 |
| 22.222068696 2604.0 |
| 22.235198979 2560.0 |
| 22.248329261 2590.0 |
| 22.261459544 2651.0 |
| 22.274589827 2764.0 |
| 22.287720109 2604.0 |
| 22.300850392 2628.0 |
| 22.313980675 2619.0 |
| 22.327110957 2630.0 |
| 22.340241240 2541.0 |
| 22.353371523 2629.0 |
| 22.366501805 2727.0 |
| 22.379632088 2586.0 |
| 22.392762371 2708.0 |
| 22.405892653 2718.0 |
| 22.419022936 2654.0 |
| 22.432153219 2748.0 |
| 22.445283501 2770.0 |
| 22.458413784 2691.0 |
| 22.471544067 2667.0 |
| 22.484674349 2674.0 |
| 22.497804632 2622.0 |
| 22.510934915 2680.0 |
| 22.524065197 2672.0 |
| 22.537195480 2706.0 |
| 22.550325763 2622.0 |
| 22.563456046 2635.0 |
| 22.576586328 2646.0 |
| 22.589716611 2655.0 |
| 22.602846894 2811.0 |
| 22.615977176 2719.0 |
| 22.629107459 2683.0 |
| 22.642237742 2748.0 |
| 22.655368024 2627.0 |
| 22.668498307 2662.0 |
| 22.681628590 2555.0 |
| 22.694758872 2847.0 |
| 22.707889155 2757.0 |
| 22.721019438 2691.0 |
| 22.734149720 2721.0 |
| 22.747280003 2811.0 |
| 22.760410286 2736.0 |
| 22.773540568 2739.0 |
| 22.786670851 2716.0 |
| 22.799801134 2684.0 |
| 22.812931416 2776.0 |
| 22.826061699 2752.0 |
| 22.839191982 2730.0 |
| 22.852322264 2670.0 |
| 22.865452547 2738.0 |
| 22.878582830 2674.0 |
| 22.891713112 2699.0 |
| 22.904843395 2732.0 |
| 22.917973678 2747.0 |
| 22.931103960 2718.0 |
| 22.944234243 2729.0 |
| 22.957364526 2721.0 |
| 22.970494808 2777.0 |
| 22.983625091 2709.0 |
| 22.996755374 2705.0 |
| 23.009885657 2796.0 |
| 23.023015939 2711.0 |
| 23.036146222 2730.0 |
| 23.049276505 2743.0 |
| 23.062406787 2864.0 |
| 23.075537070 2780.0 |
| 23.088667353 2731.0 |
| 23.101797635 2810.0 |
| 23.114927918 2778.0 |
| 23.128058201 2685.0 |
| 23.141188483 2766.0 |
| 23.154318766 2692.0 |
| 23.167449049 2784.0 |
| 23.180579331 2882.0 |
| 23.193709614 2898.0 |
| 23.206839897 2687.0 |
| 23.219970179 2812.0 |
| 23.233100462 2620.0 |
| 23.246230745 2721.0 |
| 23.259361027 2841.0 |
| 23.272491310 2782.0 |
| 23.285621593 2776.0 |
| 23.298751875 2797.0 |
| 23.311882158 2786.0 |
| 23.325012441 2798.0 |
| 23.338142723 2690.0 |
| 23.351273006 2803.0 |
| 23.364403289 2842.0 |
| 23.377533571 2932.0 |
| 23.390663854 2867.0 |
| 23.403794137 2938.0 |
| 23.416924419 2864.0 |
| 23.430054702 2800.0 |
| 23.443184985 2841.0 |
| 23.456315268 2803.0 |
| 23.469445550 2907.0 |
| 23.482575833 2858.0 |
| 23.495706116 2804.0 |
| 23.508836398 2889.0 |
| 23.521966681 2992.0 |
| 23.535096964 2910.0 |
| 23.548227246 2797.0 |
| 23.561357529 2844.0 |
| 23.574487812 2840.0 |
| 23.587618094 2862.0 |
| 23.600748377 2777.0 |
| 23.613878660 2924.0 |
| 23.627008942 2875.0 |
| 23.640139225 2875.0 |
| 23.653269508 2813.0 |
| 23.666399790 2976.0 |
| 23.679530073 2903.0 |
| 23.692660356 2864.0 |
| 23.705790638 2876.0 |
| 23.718920921 2827.0 |
| 23.732051204 2852.0 |
| 23.745181486 2899.0 |
| 23.758311769 2920.0 |
| 23.771442052 2909.0 |
| 23.784572334 2969.0 |
| 23.797702617 2883.0 |
| 23.810832900 2908.0 |
| 23.823963182 2926.0 |
| 23.837093465 2952.0 |
| 23.850223748 3033.0 |
| 23.863354030 3032.0 |
| 23.876484313 2963.0 |
| 23.889614596 2945.0 |
| 23.902744879 3023.0 |
| 23.915875161 3023.0 |
| 23.929005444 3031.0 |
| 23.942135727 2916.0 |
| 23.955266009 2936.0 |
| 23.968396292 3038.0 |
| 23.981526575 3041.0 |
| 23.994656857 3082.0 |
| 24.007787140 3063.0 |
| 24.020917423 3019.0 |
| 24.034047705 3046.0 |
| 24.047177988 3126.0 |
| 24.060308271 3095.0 |
| 24.073438553 3059.0 |
| 24.086568836 3153.0 |
| 24.099699119 3105.0 |
| 24.112829401 3146.0 |
| 24.125959684 3077.0 |
| 24.139089967 2932.0 |
| 24.152220249 3090.0 |
| 24.165350532 3232.0 |
| 24.178480815 3155.0 |
| 24.191611097 3092.0 |
| 24.204741380 3152.0 |
| 24.217871663 3184.0 |
| 24.231001945 3030.0 |
| 24.244132228 3133.0 |
| 24.257262511 3112.0 |
| 24.270392793 3092.0 |
| 24.283523076 3092.0 |
| 24.296653359 3157.0 |
| 24.309783641 3073.0 |
| 24.322913924 3063.0 |
| 24.336044207 3045.0 |
| 24.349174490 3127.0 |
| 24.362304772 3144.0 |
| 24.375435055 3122.0 |
| 24.388565338 3054.0 |
| 24.401695620 3159.0 |
| 24.414825903 3184.0 |
| 24.427956186 3208.0 |
| 24.441086468 3213.0 |
| 24.454216751 3142.0 |
| 24.467347034 3144.0 |
| 24.480477316 3122.0 |
| 24.493607599 3295.0 |
| 24.506737882 3296.0 |
| 24.519868164 3204.0 |
| 24.532998447 3279.0 |
| 24.546128730 3201.0 |
| 24.559259012 3272.0 |
| 24.572389295 3216.0 |
| 24.585519578 3228.0 |
| 24.598649860 3178.0 |
| 24.611780143 3204.0 |
| 24.624910426 3284.0 |
| 24.638040708 3260.0 |
| 24.651170991 3294.0 |
| 24.664301274 3268.0 |
| 24.677431556 3245.0 |
| 24.690561839 3295.0 |
| 24.703692122 3278.0 |
| 24.716822404 3175.0 |
| 24.729952687 3321.0 |
| 24.743082970 3271.0 |
| 24.756213253 3239.0 |
| 24.769343535 3329.0 |
| 24.782473818 3286.0 |
| 24.795604101 3333.0 |
| 24.808734383 3307.0 |
| 24.821864666 3284.0 |
| 24.834994949 3334.0 |
| 24.848125231 3405.0 |
| 24.861255514 3236.0 |
| 24.874385797 3350.0 |
| 24.887516079 3312.0 |
| 24.900646362 3339.0 |
| 24.913776645 3385.0 |
| 24.926906927 3320.0 |
| 24.940037210 3416.0 |
| 24.953167493 3355.0 |
| 24.966297775 3314.0 |
| 24.979428058 3299.0 |
| 24.992558341 3520.0 |
| 25.005688623 3386.0 |
| 25.018818906 3349.0 |
| 25.031949189 3441.0 |
| 25.045079471 3526.0 |
| 25.058209754 3441.0 |
| 25.071340037 3343.0 |
| 25.084470319 3483.0 |
| 25.097600602 3365.0 |
| 25.110730885 3441.0 |
| 25.123861167 3378.0 |
| 25.136991450 3445.0 |
| 25.150121733 3416.0 |
| 25.163252015 3457.0 |
| 25.176382298 3546.0 |
| 25.189512581 3430.0 |
| 25.202642864 3366.0 |
| 25.215773146 3485.0 |
| 25.228903429 3434.0 |
| 25.242033712 3482.0 |
| 25.255163994 3416.0 |
| 25.268294277 3359.0 |
| 25.281424560 3351.0 |
| 25.294554842 3468.0 |
| 25.307685125 3411.0 |
| 25.320815408 3465.0 |
| 25.333945690 3508.0 |
| 25.347075973 3368.0 |
| 25.360206256 3408.0 |
| 25.373336538 3308.0 |
| 25.386466821 3474.0 |
| 25.399597104 3487.0 |
| 25.412727386 3356.0 |
| 25.425857669 3478.0 |
| 25.438987952 3460.0 |
| 25.452118234 3441.0 |
| 25.465248517 3436.0 |
| 25.478378800 3452.0 |
| 25.491509082 3457.0 |
| 25.504639365 3517.0 |
| 25.517769648 3503.0 |
| 25.530899930 3412.0 |
| 25.544030213 3450.0 |
| 25.557160496 3439.0 |
| 25.570290778 3459.0 |
| 25.583421061 3483.0 |
| 25.596551344 3416.0 |
| 25.609681626 3384.0 |
| 25.622811909 3453.0 |
| 25.635942192 3337.0 |
| 25.649072475 3398.0 |
| 25.662202757 3411.0 |
| 25.675333040 3415.0 |
| 25.688463323 3381.0 |
| 25.701593605 3544.0 |
| 25.714723888 3454.0 |
| 25.727854171 3388.0 |
| 25.740984453 3399.0 |
| 25.754114736 3346.0 |
| 25.767245019 3424.0 |
| 25.780375301 3431.0 |
| 25.793505584 3374.0 |
| 25.806635867 3342.0 |
| 25.819766149 3304.0 |
| 25.832896432 3385.0 |
| 25.846026715 3341.0 |
| 25.859156997 3311.0 |
| 25.872287280 3259.0 |
| 25.885417563 3348.0 |
| 25.898547845 3255.0 |
| 25.911678128 3371.0 |
| 25.924808411 3320.0 |
| 25.937938693 3381.0 |
| 25.951068976 3268.0 |
| 25.964199259 3296.0 |
| 25.977329541 3345.0 |
| 25.990459824 3318.0 |
| 26.003590107 3286.0 |
| 26.016720389 3303.0 |
| 26.029850672 3355.0 |
| 26.042980955 3249.0 |
| 26.056111237 3334.0 |
| 26.069241520 3227.0 |
| 26.082371803 3243.0 |
| 26.095502086 3244.0 |
| 26.108632368 3238.0 |
| 26.121762651 3298.0 |
| 26.134892934 3287.0 |
| 26.148023216 3190.0 |
| 26.161153499 3168.0 |
| 26.174283782 3141.0 |
| 26.187414064 3162.0 |
| 26.200544347 3140.0 |
| 26.213674630 3245.0 |
| 26.226804912 3158.0 |
| 26.239935195 3165.0 |
| 26.253065478 3159.0 |
| 26.266195760 3129.0 |
| 26.279326043 3178.0 |
| 26.292456326 3282.0 |
| 26.305586608 3115.0 |
| 26.318716891 3137.0 |
| 26.331847174 3067.0 |
| 26.344977456 3090.0 |
| 26.358107739 3061.0 |
| 26.371238022 3093.0 |
| 26.384368304 3072.0 |
| 26.397498587 3082.0 |
| 26.410628870 2988.0 |
| 26.423759152 3001.0 |
| 26.436889435 3033.0 |
| 26.450019718 3035.0 |
| 26.463150000 3041.0 |
| 26.476280283 2990.0 |
| 26.489410566 2954.0 |
| 26.502540848 2950.0 |
| 26.515671131 3053.0 |
| 26.528801414 3012.0 |
| 26.541931697 3014.0 |
| 26.555061979 2954.0 |
| 26.568192262 2878.0 |
| 26.581322545 3003.0 |
| 26.594452827 2940.0 |
| 26.607583110 3002.0 |
| 26.620713393 2924.0 |
| 26.633843675 2878.0 |
| 26.646973958 2820.0 |
| 26.660104241 2946.0 |
| 26.673234523 2907.0 |
| 26.686364806 2913.0 |
| 26.699495089 2857.0 |
| 26.712625371 2949.0 |
| 26.725755654 2880.0 |
| 26.738885937 2870.0 |
| 26.752016219 2846.0 |
| 26.765146502 2738.0 |
| 26.778276785 2938.0 |
| 26.791407067 2844.0 |
| 26.804537350 2734.0 |
| 26.817667633 2809.0 |
| 26.830797915 2907.0 |
| 26.843928198 2923.0 |
| 26.857058481 2752.0 |
| 26.870188763 2844.0 |
| 26.883319046 2760.0 |
| 26.896449329 2875.0 |
| 26.909579611 2852.0 |
| 26.922709894 2777.0 |
| 26.935840177 2680.0 |
| 26.948970459 2793.0 |
| 26.962100742 2815.0 |
| 26.975231025 2701.0 |
| 26.988361308 2799.0 |
| 27.001491590 2777.0 |
| 27.014621873 2811.0 |
| 27.027752156 2782.0 |
| 27.040882438 2727.0 |
| 27.054012721 2830.0 |
| 27.067143004 2707.0 |
| 27.080273286 2659.0 |
| 27.093403569 2714.0 |
| 27.106533852 2658.0 |
| 27.119664134 2665.0 |
| 27.132794417 2761.0 |
| 27.145924700 2736.0 |
| 27.159054982 2710.0 |
| 27.172185265 2788.0 |
| 27.185315548 2602.0 |
| 27.198445830 2779.0 |
| 27.211576113 2759.0 |
| 27.224706396 2607.0 |
| 27.237836678 2720.0 |
| 27.250966961 2609.0 |
| 27.264097244 2589.0 |
| 27.277227526 2683.0 |
| 27.290357809 2686.0 |
| 27.303488092 2613.0 |
| 27.316618374 2634.0 |
| 27.329748657 2697.0 |
| 27.342878940 2674.0 |
| 27.356009222 2654.0 |
| 27.369139505 2712.0 |
| 27.382269788 2736.0 |
| 27.395400071 2702.0 |
| 27.408530353 2739.0 |
| 27.421660636 2697.0 |
| 27.434790919 2705.0 |
| 27.447921201 2708.0 |
| 27.461051484 2694.0 |
| 27.474181767 2793.0 |
| 27.487312049 2756.0 |
| 27.500442332 2860.0 |
| 27.513572615 2889.0 |
| 27.526702897 2883.0 |
| 27.539833180 2907.0 |
| 27.552963463 3019.0 |
| 27.566093745 3078.0 |
| 27.579224028 3051.0 |
| 27.592354311 3083.0 |
| 27.605484593 3157.0 |
| 27.618614876 3291.0 |
| 27.631745159 3481.0 |
| 27.644875441 3579.0 |
| 27.658005724 3729.0 |
| 27.671136007 3786.0 |
| 27.684266289 3911.0 |
| 27.697396572 4133.0 |
| 27.710526855 4418.0 |
| 27.723657137 4482.0 |
| 27.736787420 4530.0 |
| 27.749917703 4882.0 |
| 27.763047985 5073.0 |
| 27.776178268 5209.0 |
| 27.789308551 5315.0 |
| 27.802438833 5381.0 |
| 27.815569116 5531.0 |
| 27.828699399 5555.0 |
| 27.841829682 5548.0 |
| 27.854959964 5574.0 |
| 27.868090247 5397.0 |
| 27.881220530 5420.0 |
| 27.894350812 5209.0 |
| 27.907481095 5335.0 |
| 27.920611378 5229.0 |
| 27.933741660 5267.0 |
| 27.946871943 5157.0 |
| 27.960002226 4974.0 |
| 27.973132508 4920.0 |
| 27.986262791 4988.0 |
| 27.999393074 4916.0 |
| 28.012523356 4849.0 |
| 28.025653639 4566.0 |
| 28.038783922 4471.0 |
| 28.051914204 4378.0 |
| 28.065044487 4213.0 |
| 28.078174770 4058.0 |
| 28.091305052 3883.0 |
| 28.104435335 3759.0 |
| 28.117565618 3760.0 |
| 28.130695900 3638.0 |
| 28.143826183 3436.0 |
| 28.156956466 3330.0 |
| 28.170086748 3270.0 |
| 28.183217031 3082.0 |
| 28.196347314 3148.0 |
| 28.209477596 3009.0 |
| 28.222607879 2884.0 |
| 28.235738162 2750.0 |
| 28.248868444 2653.0 |
| 28.261998727 2651.0 |
| 28.275129010 2658.0 |
| 28.288259293 2631.0 |
| 28.301389575 2547.0 |
| 28.314519858 2468.0 |
| 28.327650141 2387.0 |
| 28.340780423 2373.0 |
| 28.353910706 2345.0 |
| 28.367040989 2315.0 |
| 28.380171271 2347.0 |
| 28.393301554 2378.0 |
| 28.406431837 2297.0 |
| 28.419562119 2304.0 |
| 28.432692402 2222.0 |
| 28.445822685 2265.0 |
| 28.458952967 2303.0 |
| 28.472083250 2241.0 |
| 28.485213533 2160.0 |
| 28.498343815 2185.0 |
| 28.511474098 2220.0 |
| 28.524604381 2237.0 |
| 28.537734663 2251.0 |
| 28.550864946 2215.0 |
| 28.563995229 2183.0 |
| 28.577125511 2132.0 |
| 28.590255794 2198.0 |
| 28.603386077 2203.0 |
| 28.616516359 2193.0 |
| 28.629646642 2126.0 |
| 28.642776925 2185.0 |
| 28.655907207 2141.0 |
| 28.669037490 2166.0 |
| 28.682167773 2094.0 |
| 28.695298055 2163.0 |
| 28.708428338 2150.0 |
| 28.721558621 2156.0 |
| 28.734688904 2054.0 |
| 28.747819186 2087.0 |
| 28.760949469 2075.0 |
| 28.774079752 2125.0 |
| 28.787210034 2078.0 |
| 28.800340317 2120.0 |
| 28.813470600 2171.0 |
| 28.826600882 2090.0 |
| 28.839731165 2151.0 |
| 28.852861448 2081.0 |
| 28.865991730 2087.0 |
| 28.879122013 2254.0 |
| 28.892252296 2078.0 |
| 28.905382578 2149.0 |
| 28.918512861 2102.0 |
| 28.931643144 2002.0 |
| 28.944773426 2095.0 |
| 28.957903709 2078.0 |
| 28.971033992 2044.0 |
| 28.984164274 2083.0 |
| 28.997294557 2144.0 |
| 29.010424840 2162.0 |
| 29.023555122 2161.0 |
| 29.036685405 2153.0 |
| 29.049815688 2123.0 |
| 29.062945970 2079.0 |
| 29.076076253 2065.0 |
| 29.089206536 2114.0 |
| 29.102336818 2034.0 |
| 29.115467101 2073.0 |
| 29.128597384 1974.0 |
| 29.141727666 2070.0 |
| 29.154857949 2013.0 |
| 29.167988232 2173.0 |
| 29.181118515 2185.0 |
| 29.194248797 2107.0 |
| 29.207379080 2118.0 |
| 29.220509363 2054.0 |
| 29.233639645 2030.0 |
| 29.246769928 2121.0 |
| 29.259900211 2012.0 |
| 29.273030493 1941.0 |
| 29.286160776 2100.0 |
| 29.299291059 2075.0 |
| 29.312421341 2074.0 |
| 29.325551624 2131.0 |
| 29.338681907 2003.0 |
| 29.351812189 2171.0 |
| 29.364942472 2109.0 |
| 29.378072755 2148.0 |
| 29.391203037 2039.0 |
| 29.404333320 2154.0 |
| 29.417463603 2141.0 |
| 29.430593885 2146.0 |
| 29.443724168 2022.0 |
| 29.456854451 2194.0 |
| 29.469984733 2093.0 |
| 29.483115016 2114.0 |
| 29.496245299 2098.0 |
| 29.509375581 2081.0 |
| 29.522505864 2062.0 |
| 29.535636147 2142.0 |
| 29.548766429 2200.0 |
| 29.561896712 2163.0 |
| 29.575026995 2048.0 |
| 29.588157278 2068.0 |
| 29.601287560 2049.0 |
| 29.614417843 2060.0 |
| 29.627548126 2059.0 |
| 29.640678408 2078.0 |
| 29.653808691 2123.0 |
| 29.666938974 2066.0 |
| 29.680069256 2131.0 |
| 29.693199539 2071.0 |
| 29.706329822 2029.0 |
| 29.719460104 2043.0 |
| 29.732590387 2092.0 |
| 29.745720670 1997.0 |
| 29.758850952 1914.0 |
| 29.771981235 2103.0 |
| 29.785111518 1991.0 |
| 29.798241800 2019.0 |
| 29.811372083 1985.0 |
| 29.824502366 2030.0 |
| 29.837632648 2004.0 |
| 29.850762931 1990.0 |
| 29.863893214 2134.0 |
| 29.877023496 2104.0 |
| 29.890153779 2083.0 |
| 29.903284062 2051.0 |
| 29.916414344 2031.0 |
| 29.929544627 2024.0 |
| 29.942674910 1994.0 |
| 29.955805192 2045.0 |
| 29.968935475 1996.0 |
| 29.982065758 2042.0 |
| 29.995196040 1999.0 |
| 30.008326323 1956.0 |
| 30.021456606 2054.0 |
| 30.034586889 1945.0 |
| 30.047717171 1983.0 |
| 30.060847454 2022.0 |
| 30.073977737 2041.0 |
| 30.087108019 2011.0 |
| 30.100238302 2008.0 |
| 30.113368585 2058.0 |
| 30.126498867 2007.0 |
| 30.139629150 2000.0 |
| 30.152759433 1948.0 |
| 30.165889715 1969.0 |
| 30.179019998 1954.0 |
| 30.192150281 2071.0 |
| 30.205280563 2106.0 |
| 30.218410846 2104.0 |
| 30.231541129 1988.0 |
| 30.244671411 2012.0 |
| 30.257801694 2003.0 |
| 30.270931977 1964.0 |
| 30.284062259 2035.0 |
| 30.297192542 1991.0 |
| 30.310322825 2065.0 |
| 30.323453107 2030.0 |
| 30.336583390 1954.0 |
| 30.349713673 2128.0 |
| 30.362843955 1941.0 |
| 30.375974238 2018.0 |
| 30.389104521 2074.0 |
| 30.402234803 2040.0 |
| 30.415365086 2055.0 |
| 30.428495369 2004.0 |
| 30.441625651 2031.0 |
| 30.454755934 2081.0 |
| 30.467886217 1971.0 |
| 30.481016500 1984.0 |
| 30.494146782 2004.0 |
| 30.507277065 1980.0 |
| 30.520407348 2031.0 |
| 30.533537630 2094.0 |
| 30.546667913 2010.0 |
| 30.559798196 2004.0 |
| 30.572928478 2142.0 |
| 30.586058761 1994.0 |
| 30.599189044 2043.0 |
| 30.612319326 2059.0 |
| 30.625449609 2132.0 |
| 30.638579892 2102.0 |
| 30.651710174 2091.0 |
| 30.664840457 2018.0 |
| 30.677970740 2076.0 |
| 30.691101022 2087.0 |
| 30.704231305 2072.0 |
| 30.717361588 2066.0 |
| 30.730491870 2154.0 |
| 30.743622153 2011.0 |
| 30.756752436 2086.0 |
| 30.769882718 2026.0 |
| 30.783013001 2059.0 |
| 30.796143284 2107.0 |
| 30.809273566 2034.0 |
| 30.822403849 2097.0 |
| 30.835534132 2096.0 |
| 30.848664414 2062.0 |
| 30.861794697 2233.0 |
| 30.874924980 2140.0 |
| 30.888055262 2121.0 |
| 30.901185545 2116.0 |
| 30.914315828 2079.0 |
| 30.927446111 2048.0 |
| 30.940576393 1997.0 |
| 30.953706676 2108.0 |
| 30.966836959 2088.0 |
| 30.979967241 2088.0 |
| 30.993097524 1991.0 |
| 31.006227807 2019.0 |
| 31.019358089 2026.0 |
| 31.032488372 2059.0 |
| 31.045618655 1978.0 |
| 31.058748937 2121.0 |
| 31.071879220 1962.0 |
| 31.085009503 1981.0 |
| 31.098139785 1930.0 |
| 31.111270068 1931.0 |
| 31.124400351 1967.0 |
| 31.137530633 2009.0 |
| 31.150660916 1951.0 |
| 31.163791199 1909.0 |
| 31.176921481 1924.0 |
| 31.190051764 1998.0 |
| 31.203182047 1912.0 |
| 31.216312329 1910.0 |
| 31.229442612 1969.0 |
| 31.242572895 1918.0 |
| 31.255703177 1998.0 |
| 31.268833460 1965.0 |
| 31.281963743 1903.0 |
| 31.295094025 1856.0 |
| 31.308224308 1888.0 |
| 31.321354591 1873.0 |
| 31.334484873 1964.0 |
| 31.347615156 1900.0 |
| 31.360745439 1962.0 |
| 31.373875722 1907.0 |
| 31.387006004 1883.0 |
| 31.400136287 1901.0 |
| 31.413266570 1888.0 |
| 31.426396852 1930.0 |
| 31.439527135 1787.0 |
| 31.452657418 1856.0 |
| 31.465787700 1837.0 |
| 31.478917983 1835.0 |
| 31.492048266 1864.0 |
| 31.505178548 1869.0 |
| 31.518308831 1894.0 |
| 31.531439114 1898.0 |
| 31.544569396 1831.0 |
| 31.557699679 1847.0 |
| 31.570829962 1861.0 |
| 31.583960244 1832.0 |
| 31.597090527 1881.0 |
| 31.610220810 1863.0 |
| 31.623351092 1793.0 |
| 31.636481375 1804.0 |
| 31.649611658 1952.0 |
| 31.662741940 1877.0 |
| 31.675872223 1895.0 |
| 31.689002506 1855.0 |
| 31.702132788 1825.0 |
| 31.715263071 1853.0 |
| 31.728393354 1848.0 |
| 31.741523636 1891.0 |
| 31.754653919 1860.0 |
| 31.767784202 1825.0 |
| 31.780914484 1922.0 |
| 31.794044767 1893.0 |
| 31.807175050 1835.0 |
| 31.820305333 1842.0 |
| 31.833435615 1800.0 |
| 31.846565898 1893.0 |
| 31.859696181 1833.0 |
| 31.872826463 1789.0 |
| 31.885956746 1850.0 |
| 31.899087029 1836.0 |
| 31.912217311 1729.0 |
| 31.925347594 1860.0 |
| 31.938477877 1867.0 |
| 31.951608159 1896.0 |
| 31.964738442 1900.0 |
| 31.977868725 1870.0 |
| 31.990999007 1835.0 |
| 32.004129290 1755.0 |
| 32.017259573 1868.0 |
| 32.030389855 1870.0 |
| 32.043520138 1786.0 |
| 32.056650421 1895.0 |
| 32.069780703 1798.0 |
| 32.082910986 1744.0 |
| 32.096041269 1763.0 |
| 32.109171551 1753.0 |
| 32.122301834 1722.0 |
| 32.135432117 1746.0 |
| 32.148562399 1790.0 |
| 32.161692682 1814.0 |
| 32.174822965 1804.0 |
| 32.187953247 1762.0 |
| 32.201083530 1829.0 |
| 32.214213813 1733.0 |
| 32.227344096 1739.0 |
| 32.240474378 1708.0 |
| 32.253604661 1782.0 |
| 32.266734944 1813.0 |
| 32.279865226 1860.0 |
| 32.292995509 1746.0 |
| 32.306125792 1750.0 |
| 32.319256074 1800.0 |
| 32.332386357 1759.0 |
| 32.345516640 1722.0 |
| 32.358646922 1730.0 |
| 32.371777205 1832.0 |
| 32.384907488 1815.0 |
| 32.398037770 1853.0 |
| 32.411168053 1778.0 |
| 32.424298336 1756.0 |
| 32.437428618 1848.0 |
| 32.450558901 1753.0 |
| 32.463689184 1796.0 |
| 32.476819466 1813.0 |
| 32.489949749 1685.0 |
| 32.503080032 1654.0 |
| 32.516210314 1728.0 |
| 32.529340597 1776.0 |
| 32.542470880 1764.0 |
| 32.555601162 1709.0 |
| 32.568731445 1771.0 |
| 32.581861728 1724.0 |
| 32.594992010 1733.0 |
| 32.608122293 1730.0 |
| 32.621252576 1715.0 |
| 32.634382858 1758.0 |
| 32.647513141 1799.0 |
| 32.660643424 1778.0 |
| 32.673773707 1721.0 |
| 32.686903989 1741.0 |
| 32.700034272 1719.0 |
| 32.713164555 1753.0 |
| 32.726294837 1811.0 |
| 32.739425120 1781.0 |
| 32.752555403 1752.0 |
| 32.765685685 1721.0 |
| 32.778815968 1887.0 |
| 32.791946251 1816.0 |
| 32.805076533 1769.0 |
| 32.818206816 1833.0 |
| 32.831337099 1841.0 |
| 32.844467381 1746.0 |
| 32.857597664 1791.0 |
| 32.870727947 1873.0 |
| 32.883858229 1859.0 |
| 32.896988512 1697.0 |
| 32.910118795 1786.0 |
| 32.923249077 1717.0 |
| 32.936379360 1782.0 |
| 32.949509643 1747.0 |
| 32.962639925 1834.0 |
| 32.975770208 1794.0 |
| 32.988900491 1794.0 |
| 33.002030773 1684.0 |
| 33.015161056 1818.0 |
| 33.028291339 1810.0 |
| 33.041421621 1891.0 |
| 33.054551904 1766.0 |
| 33.067682187 1715.0 |
| 33.080812469 1672.0 |
| 33.093942752 1761.0 |
| 33.107073035 1757.0 |
| 33.120203318 1730.0 |
| 33.133333600 1622.0 |
| 33.146463883 1739.0 |
| 33.159594166 1782.0 |
| 33.172724448 1750.0 |
| 33.185854731 1723.0 |
| 33.198985014 1788.0 |
| 33.212115296 1746.0 |
| 33.225245579 1690.0 |
| 33.238375862 1785.0 |
| 33.251506144 1792.0 |
| 33.264636427 1717.0 |
| 33.277766710 1770.0 |
| 33.290896992 1694.0 |
| 33.304027275 1756.0 |
| 33.317157558 1796.0 |
| 33.330287840 1780.0 |
| 33.343418123 1679.0 |
| 33.356548406 1749.0 |
| 33.369678688 1702.0 |
| 33.382808971 1713.0 |
| 33.395939254 1698.0 |
| 33.409069536 1696.0 |
| 33.422199819 1743.0 |
| 33.435330102 1677.0 |
| 33.448460384 1638.0 |
| 33.461590667 1659.0 |
| 33.474720950 1710.0 |
| 33.487851232 1694.0 |
| 33.500981515 1760.0 |
| 33.514111798 1784.0 |
| 33.527242080 1735.0 |
| 33.540372363 1729.0 |
| 33.553502646 1767.0 |
| 33.566632929 1688.0 |
| 33.579763211 1710.0 |
| 33.592893494 1719.0 |
| 33.606023777 1671.0 |
| 33.619154059 1733.0 |
| 33.632284342 1773.0 |
| 33.645414625 1725.0 |
| 33.658544907 1732.0 |
| 33.671675190 1734.0 |
| 33.684805473 1790.0 |
| 33.697935755 1718.0 |
| 33.711066038 1725.0 |
| 33.724196321 1807.0 |
| 33.737326603 1724.0 |
| 33.750456886 1761.0 |
| 33.763587169 1751.0 |
| 33.776717451 1713.0 |
| 33.789847734 1770.0 |
| 33.802978017 1689.0 |
| 33.816108299 1754.0 |
| 33.829238582 1691.0 |
| 33.842368865 1749.0 |
| 33.855499147 1842.0 |
| 33.868629430 1666.0 |
| 33.881759713 1717.0 |
| 33.894889995 1728.0 |
| 33.908020278 1717.0 |
| 33.921150561 1740.0 |
| 33.934280843 1743.0 |
| 33.947411126 1756.0 |
| 33.960541409 1730.0 |
| 33.973671691 1728.0 |
| 33.986801974 1724.0 |
| 33.999932257 1717.0 |
| 34.013062540 1705.0 |
| 34.026192822 1769.0 |
| 34.039323105 1665.0 |
| 34.052453388 1711.0 |
| 34.065583670 1712.0 |
| 34.078713953 1740.0 |
| 34.091844236 1833.0 |
| 34.104974518 1764.0 |
| 34.118104801 1724.0 |
| 34.131235084 1725.0 |
| 34.144365366 1735.0 |
| 34.157495649 1787.0 |
| 34.170625932 1766.0 |
| 34.183756214 1760.0 |
| 34.196886497 1777.0 |
| 34.210016780 1784.0 |
| 34.223147062 1696.0 |
| 34.236277345 1687.0 |
| 34.249407628 1698.0 |
| 34.262537910 1788.0 |
| 34.275668193 1736.0 |
| 34.288798476 1811.0 |
| 34.301928758 1722.0 |
| 34.315059041 1807.0 |
| 34.328189324 1668.0 |
| 34.341319606 1763.0 |
| 34.354449889 1746.0 |
| 34.367580172 1808.0 |
| 34.380710454 1709.0 |
| 34.393840737 1815.0 |
| 34.406971020 1732.0 |
| 34.420101303 1720.0 |
| 34.433231585 1790.0 |
| 34.446361868 1773.0 |
| 34.459492151 1780.0 |
| 34.472622433 1706.0 |
| 34.485752716 1761.0 |
| 34.498882999 1788.0 |
| 34.512013281 1797.0 |
| 34.525143564 1748.0 |
| 34.538273847 1730.0 |
| 34.551404129 1768.0 |
| 34.564534412 1716.0 |
| 34.577664695 1705.0 |
| 34.590794977 1784.0 |
| 34.603925260 1710.0 |
| 34.617055543 1729.0 |
| 34.630185825 1794.0 |
| 34.643316108 1794.0 |
| 34.656446391 1805.0 |
| 34.669576673 1811.0 |
| 34.682706956 1779.0 |
| 34.695837239 1787.0 |
| 34.708967521 1749.0 |
| 34.722097804 1794.0 |
| 34.735228087 1807.0 |
| 34.748358369 1739.0 |
| 34.761488652 1758.0 |
| 34.774618935 1841.0 |
| 34.787749217 1867.0 |
| 34.800879500 1703.0 |
| 34.814009783 1807.0 |
| 34.827140065 1839.0 |
| 34.840270348 1721.0 |
| 34.853400631 1809.0 |
| 34.866530914 1754.0 |
| 34.879661196 1687.0 |
| 34.892791479 1714.0 |
| 34.905921762 1788.0 |
| 34.919052044 1744.0 |
| 34.932182327 1708.0 |
| 34.945312610 1779.0 |
| 34.958442892 1782.0 |
| 34.971573175 1765.0 |
| 34.984703458 1782.0 |
| 34.997833740 1780.0 |
| 35.010964023 1775.0 |
| 35.024094306 1753.0 |
| 35.037224588 1605.0 |
| 35.050354871 1729.0 |
| 35.063485154 1704.0 |
| 35.076615436 1717.0 |
| 35.089745719 1717.0 |
| 35.102876002 1721.0 |
| 35.116006284 1649.0 |
| 35.129136567 1695.0 |
| 35.142266850 1682.0 |
| 35.155397132 1708.0 |
| 35.168527415 1780.0 |
| 35.181657698 1685.0 |
| 35.194787980 1736.0 |
| 35.207918263 1705.0 |
| 35.221048546 1727.0 |
| 35.234178828 1711.0 |
| 35.247309111 1807.0 |
| 35.260439394 1773.0 |
| 35.273569676 1757.0 |
| 35.286699959 1698.0 |
| 35.299830242 1688.0 |
| 35.312960525 1718.0 |
| 35.326090807 1716.0 |
| 35.339221090 1629.0 |
| 35.352351373 1757.0 |
| 35.365481655 1684.0 |
| 35.378611938 1732.0 |
| 35.391742221 1641.0 |
| 35.404872503 1657.0 |
| 35.418002786 1625.0 |
| 35.431133069 1713.0 |
| 35.444263351 1656.0 |
| 35.457393634 1587.0 |
| 35.470523917 1717.0 |
| 35.483654199 1690.0 |
| 35.496784482 1626.0 |
| 35.509914765 1686.0 |
| 35.523045047 1637.0 |
| 35.536175330 1730.0 |
| 35.549305613 1728.0 |
| 35.562435895 1688.0 |
| 35.575566178 1706.0 |
| 35.588696461 1678.0 |
| 35.601826743 1691.0 |
| 35.614957026 1651.0 |
| 35.628087309 1756.0 |
| 35.641217591 1583.0 |
| 35.654347874 1690.0 |
| 35.667478157 1755.0 |
| 35.680608439 1696.0 |
| 35.693738722 1721.0 |
| 35.706869005 1666.0 |
| 35.719999287 1655.0 |
| 35.733129570 1704.0 |
| 35.746259853 1723.0 |
| 35.759390136 1718.0 |
| 35.772520418 1730.0 |
| 35.785650701 1752.0 |
| 35.798780984 1770.0 |
| 35.811911266 1690.0 |
| 35.825041549 1622.0 |
| 35.838171832 1770.0 |
| 35.851302114 1689.0 |
| 35.864432397 1653.0 |
| 35.877562680 1736.0 |
| 35.890692962 1728.0 |
| 35.903823245 1695.0 |
| 35.916953528 1699.0 |
| 35.930083810 1642.0 |
| 35.943214093 1691.0 |
| 35.956344376 1781.0 |
| 35.969474658 1728.0 |
| 35.982604941 1721.0 |
| 35.995735224 1722.0 |
| 36.008865506 1613.0 |
| 36.021995789 1651.0 |
| 36.035126072 1757.0 |
| 36.048256354 1613.0 |
| 36.061386637 1686.0 |
| 36.074516920 1718.0 |
| 36.087647202 1672.0 |
| 36.100777485 1649.0 |
| 36.113907768 1750.0 |
| 36.127038050 1663.0 |
| 36.140168333 1754.0 |
| 36.153298616 1765.0 |
| 36.166428898 1768.0 |
| 36.179559181 1667.0 |
| 36.192689464 1668.0 |
| 36.205819747 1740.0 |
| 36.218950029 1753.0 |
| 36.232080312 1632.0 |
| 36.245210595 1743.0 |
| 36.258340877 1745.0 |
| 36.271471160 1716.0 |
| 36.284601443 1700.0 |
| 36.297731725 1716.0 |
| 36.310862008 1692.0 |
| 36.323992291 1709.0 |
| 36.337122573 1680.0 |
| 36.350252856 1721.0 |
| 36.363383139 1715.0 |
| 36.376513421 1726.0 |
| 36.389643704 1747.0 |
| 36.402773987 1746.0 |
| 36.415904269 1755.0 |
| 36.429034552 1657.0 |
| 36.442164835 1704.0 |
| 36.455295117 1695.0 |
| 36.468425400 1671.0 |
| 36.481555683 1705.0 |
| 36.494685965 1729.0 |
| 36.507816248 1777.0 |
| 36.520946531 1672.0 |
| 36.534076813 1706.0 |
| 36.547207096 1715.0 |
| 36.560337379 1684.0 |
| 36.573467661 1683.0 |
| 36.586597944 1699.0 |
| 36.599728227 1695.0 |
| 36.612858509 1730.0 |
| 36.625988792 1666.0 |
| 36.639119075 1708.0 |
| 36.652249358 1712.0 |
| 36.665379640 1813.0 |
| 36.678509923 1759.0 |
| 36.691640206 1704.0 |
| 36.704770488 1694.0 |
| 36.717900771 1723.0 |
| 36.731031054 1721.0 |
| 36.744161336 1704.0 |
| 36.757291619 1686.0 |
| 36.770421902 1671.0 |
| 36.783552184 1713.0 |
| 36.796682467 1714.0 |
| 36.809812750 1708.0 |
| 36.822943032 1718.0 |
| 36.836073315 1766.0 |
| 36.849203598 1630.0 |
| 36.862333880 1819.0 |
| 36.875464163 1785.0 |
| 36.888594446 1766.0 |
| 36.901724728 1693.0 |
| 36.914855011 1686.0 |
| 36.927985294 1663.0 |
| 36.941115576 1709.0 |
| 36.954245859 1777.0 |
| 36.967376142 1643.0 |
| 36.980506424 1687.0 |
| 36.993636707 1752.0 |
| 37.006766990 1769.0 |
| 37.019897272 1695.0 |
| 37.033027555 1752.0 |
| 37.046157838 1689.0 |
| 37.059288121 1756.0 |
| 37.072418403 1747.0 |
| 37.085548686 1673.0 |
| 37.098678969 1744.0 |
| 37.111809251 1782.0 |
| 37.124939534 1675.0 |
| 37.138069817 1717.0 |
| 37.151200099 1758.0 |
| 37.164330382 1806.0 |
| 37.177460665 1655.0 |
| 37.190590947 1737.0 |
| 37.203721230 1790.0 |
| 37.216851513 1799.0 |
| 37.229981795 1757.0 |
| 37.243112078 1795.0 |
| 37.256242361 1680.0 |
| 37.269372643 1788.0 |
| 37.282502926 1705.0 |
| 37.295633209 1722.0 |
| 37.308763491 1736.0 |
| 37.321893774 1712.0 |
| 37.335024057 1721.0 |
| 37.348154339 1791.0 |
| 37.361284622 1712.0 |
| 37.374414905 1707.0 |
| 37.387545187 1703.0 |
| 37.400675470 1750.0 |
| 37.413805753 1734.0 |
| 37.426936035 1726.0 |
| 37.440066318 1743.0 |
| 37.453196601 1844.0 |
| 37.466326883 1682.0 |
| 37.479457166 1670.0 |
| 37.492587449 1668.0 |
| 37.505717732 1776.0 |
| 37.518848014 1768.0 |
| 37.531978297 1788.0 |
| 37.545108580 1722.0 |
| 37.558238862 1681.0 |
| 37.571369145 1754.0 |
| 37.584499428 1713.0 |
| 37.597629710 1795.0 |
| 37.610759993 1819.0 |
| 37.623890276 1787.0 |
| 37.637020558 1831.0 |
| 37.650150841 1722.0 |
| 37.663281124 1799.0 |
| 37.676411406 1759.0 |
| 37.689541689 1677.0 |
| 37.702671972 1778.0 |
| 37.715802254 1759.0 |
| 37.728932537 1763.0 |
| 37.742062820 1726.0 |
| 37.755193102 1743.0 |
| 37.768323385 1797.0 |
| 37.781453668 1738.0 |
| 37.794583950 1736.0 |
| 37.807714233 1723.0 |
| 37.820844516 1792.0 |
| 37.833974798 1788.0 |
| 37.847105081 1706.0 |
| 37.860235364 1739.0 |
| 37.873365646 1757.0 |
| 37.886495929 1805.0 |
| 37.899626212 1791.0 |
| 37.912756494 1811.0 |
| 37.925886777 1743.0 |
| 37.939017060 1799.0 |
| 37.952147343 1830.0 |
| 37.965277625 1669.0 |
| 37.978407908 1858.0 |
| 37.991538191 1877.0 |
| 38.004668473 1788.0 |
| 38.017798756 1791.0 |
| 38.030929039 1741.0 |
| 38.044059321 1788.0 |
| 38.057189604 1856.0 |
| 38.070319887 1782.0 |
| 38.083450169 1895.0 |
| 38.096580452 1895.0 |
| 38.109710735 1809.0 |
| 38.122841017 1729.0 |
| 38.135971300 1811.0 |
| 38.149101583 1832.0 |
| 38.162231865 1845.0 |
| 38.175362148 1831.0 |
| 38.188492431 1863.0 |
| 38.201622713 1843.0 |
| 38.214752996 1857.0 |
| 38.227883279 1868.0 |
| 38.241013561 1859.0 |
| 38.254143844 1912.0 |
| 38.267274127 1847.0 |
| 38.280404409 1899.0 |
| 38.293534692 1854.0 |
| 38.306664975 1852.0 |
| 38.319795257 1905.0 |
| 38.332925540 1812.0 |
| 38.346055823 1848.0 |
| 38.359186105 1877.0 |
| 38.372316388 1915.0 |
| 38.385446671 1829.0 |
| 38.398576954 1881.0 |
| 38.411707236 1905.0 |
| 38.424837519 1885.0 |
| 38.437967802 1814.0 |
| 38.451098084 1852.0 |
| 38.464228367 1905.0 |
| 38.477358650 1835.0 |
| 38.490488932 1852.0 |
| 38.503619215 1928.0 |
| 38.516749498 1945.0 |
| 38.529879780 1890.0 |
| 38.543010063 1904.0 |
| 38.556140346 1919.0 |
| 38.569270628 1849.0 |
| 38.582400911 1892.0 |
| 38.595531194 1919.0 |
| 38.608661476 1959.0 |
| 38.621791759 2031.0 |
| 38.634922042 1937.0 |
| 38.648052324 1972.0 |
| 38.661182607 1941.0 |
| 38.674312890 1876.0 |
| 38.687443172 1964.0 |
| 38.700573455 1902.0 |
| 38.713703738 1999.0 |
| 38.726834020 1975.0 |
| 38.739964303 1945.0 |
| 38.753094586 1932.0 |
| 38.766224868 1972.0 |
| 38.779355151 1967.0 |
| 38.792485434 2007.0 |
| 38.805615716 2010.0 |
| 38.818745999 1971.0 |
| 38.831876282 2043.0 |
| 38.845006565 2043.0 |
| 38.858136847 2000.0 |
| 38.871267130 2021.0 |
| 38.884397413 2077.0 |
| 38.897527695 2094.0 |
| 38.910657978 2056.0 |
| 38.923788261 2109.0 |
| 38.936918543 2086.0 |
| 38.950048826 2016.0 |
| 38.963179109 2114.0 |
| 38.976309391 2066.0 |
| 38.989439674 2105.0 |
| 39.002569957 2134.0 |
| 39.015700239 2052.0 |
| 39.028830522 2139.0 |
| 39.041960805 2182.0 |
| 39.055091087 2091.0 |
| 39.068221370 2018.0 |
| 39.081351653 2093.0 |
| 39.094481935 2212.0 |
| 39.107612218 2124.0 |
| 39.120742501 2172.0 |
| 39.133872783 2148.0 |
| 39.147003066 2181.0 |
| 39.160133349 2184.0 |
| 39.173263631 2147.0 |
| 39.186393914 2068.0 |
| 39.199524197 2231.0 |
| 39.212654479 2133.0 |
| 39.225784762 2143.0 |
| 39.238915045 2125.0 |
| 39.252045327 2203.0 |
| 39.265175610 2119.0 |
| 39.278305893 2181.0 |
| 39.291436176 2098.0 |
| 39.304566458 2163.0 |
| 39.317696741 2257.0 |
| 39.330827024 2197.0 |
| 39.343957306 2198.0 |
| 39.357087589 2261.0 |
| 39.370217872 2214.0 |
| 39.383348154 2335.0 |
| 39.396478437 2303.0 |
| 39.409608720 2292.0 |
| 39.422739002 2202.0 |
| 39.435869285 2329.0 |
| 39.448999568 2228.0 |
| 39.462129850 2312.0 |
| 39.475260133 2202.0 |
| 39.488390416 2357.0 |
| 39.501520698 2216.0 |
| 39.514650981 2277.0 |
| 39.527781264 2314.0 |
| 39.540911546 2339.0 |
| 39.554041829 2235.0 |
| 39.567172112 2373.0 |
| 39.580302394 2364.0 |
| 39.593432677 2335.0 |
| 39.606562960 2460.0 |
| 39.619693242 2321.0 |
| 39.632823525 2436.0 |
| 39.645953808 2406.0 |
| 39.659084090 2528.0 |
| 39.672214373 2356.0 |
| 39.685344656 2508.0 |
| 39.698474939 2497.0 |
| 39.711605221 2499.0 |
| 39.724735504 2492.0 |
| 39.737865787 2368.0 |
| 39.750996069 2546.0 |
| 39.764126352 2513.0 |
| 39.777256635 2582.0 |
| 39.790386917 2552.0 |
| 39.803517200 2639.0 |
| 39.816647483 2647.0 |
| 39.829777765 2627.0 |
| 39.842908048 2657.0 |
| 39.856038331 2662.0 |
| 39.869168613 2650.0 |
| 39.882298896 2539.0 |
| 39.895429179 2638.0 |
| 39.908559461 2697.0 |
| 39.921689744 2602.0 |
| 39.934820027 2658.0 |
| 39.947950309 2727.0 |
| 39.961080592 2615.0 |
| 39.974210875 2655.0 |
| 39.987341157 2659.0 |
| 40.000471440 2621.0 |
| 40.013601723 2708.0 |
| 40.026732005 2719.0 |
| 40.039862288 2690.0 |
| 40.052992571 2724.0 |
| 40.066122853 2753.0 |
| 40.079253136 2743.0 |
| 40.092383419 2744.0 |
| 40.105513701 2733.0 |
| 40.118643984 2644.0 |
| 40.131774267 2695.0 |
| 40.144904550 2740.0 |
| 40.158034832 2606.0 |
| 40.171165115 2704.0 |
| 40.184295398 2843.0 |
| 40.197425680 2793.0 |
| 40.210555963 2809.0 |
| 40.223686246 2820.0 |
| 40.236816528 2784.0 |
| 40.249946811 2762.0 |
| 40.263077094 2710.0 |
| 40.276207376 2806.0 |
| 40.289337659 2758.0 |
| 40.302467942 2796.0 |
| 40.315598224 2781.0 |
| 40.328728507 2820.0 |
| 40.341858790 2878.0 |
| 40.354989072 2905.0 |
| 40.368119355 2725.0 |
| 40.381249638 2838.0 |
| 40.394379920 2850.0 |
| 40.407510203 2799.0 |
| 40.420640486 2803.0 |
| 40.433770768 2805.0 |
| 40.446901051 2801.0 |
| 40.460031334 2814.0 |
| 40.473161616 2814.0 |
| 40.486291899 2856.0 |
| 40.499422182 2894.0 |
| 40.512552464 2875.0 |
| 40.525682747 2936.0 |
| 40.538813030 2888.0 |
| 40.551943312 2831.0 |
| 40.565073595 2856.0 |
| 40.578203878 2878.0 |
| 40.591334161 2803.0 |
| 40.604464443 2832.0 |
| 40.617594726 2847.0 |
| 40.630725009 2949.0 |
| 40.643855291 2869.0 |
| 40.656985574 2914.0 |
| 40.670115857 2942.0 |
| 40.683246139 2916.0 |
| 40.696376422 3008.0 |
| 40.709506705 2882.0 |
| 40.722636987 3013.0 |
| 40.735767270 3011.0 |
| 40.748897553 2964.0 |
| 40.762027835 2992.0 |
| 40.775158118 3041.0 |
| 40.788288401 3002.0 |
| 40.801418683 3068.0 |
| 40.814548966 3065.0 |
| 40.827679249 3064.0 |
| 40.840809531 3020.0 |
| 40.853939814 3017.0 |
| 40.867070097 3023.0 |
| 40.880200379 3085.0 |
| 40.893330662 3050.0 |
| 40.906460945 3024.0 |
| 40.919591227 3008.0 |
| 40.932721510 3127.0 |
| 40.945851793 3157.0 |
| 40.958982075 2996.0 |
| 40.972112358 3026.0 |
| 40.985242641 2990.0 |
| 40.998372923 3073.0 |
| 41.011503206 3033.0 |
| 41.024633489 3109.0 |
| 41.037763772 3082.0 |
| 41.050894054 3009.0 |
| 41.064024337 3070.0 |
| 41.077154620 3056.0 |
| 41.090284902 2991.0 |
| 41.103415185 3056.0 |
| 41.116545468 3083.0 |
| 41.129675750 3156.0 |
| 41.142806033 2979.0 |
| 41.155936316 3003.0 |
| 41.169066598 3097.0 |
| 41.182196881 3049.0 |
| 41.195327164 3108.0 |
| 41.208457446 3102.0 |
| 41.221587729 3056.0 |
| 41.234718012 3159.0 |
| 41.247848294 3068.0 |
| 41.260978577 3123.0 |
| 41.274108860 3081.0 |
| 41.287239142 3219.0 |
| 41.300369425 3038.0 |
| 41.313499708 3051.0 |
| 41.326629990 3138.0 |
| 41.339760273 3079.0 |
| 41.352890556 3051.0 |
| 41.366020838 3055.0 |
| 41.379151121 3101.0 |
| 41.392281404 2994.0 |
| 41.405411686 3034.0 |
| 41.418541969 2953.0 |
| 41.431672252 3037.0 |
| 41.444802534 3123.0 |
| 41.457932817 3170.0 |
| 41.471063100 3160.0 |
| 41.484193383 3003.0 |
| 41.497323665 2975.0 |
| 41.510453948 2994.0 |
| 41.523584231 3037.0 |
| 41.536714513 2918.0 |
| 41.549844796 3017.0 |
| 41.562975079 3026.0 |
| 41.576105361 3045.0 |
| 41.589235644 3060.0 |
| 41.602365927 2948.0 |
| 41.615496209 2885.0 |
| 41.628626492 2963.0 |
| 41.641756775 3008.0 |
| 41.654887057 3029.0 |
| 41.668017340 3050.0 |
| 41.681147623 2932.0 |
| 41.694277905 2886.0 |
| 41.707408188 2928.0 |
| 41.720538471 2870.0 |
| 41.733668753 2984.0 |
| 41.746799036 2943.0 |
| 41.759929319 2937.0 |
| 41.773059601 2870.0 |
| 41.786189884 2868.0 |
| 41.799320167 2885.0 |
| 41.812450449 2911.0 |
| 41.825580732 2808.0 |
| 41.838711015 2831.0 |
| 41.851841297 2805.0 |
| 41.864971580 2799.0 |
| 41.878101863 2827.0 |
| 41.891232146 2837.0 |
| 41.904362428 2839.0 |
| 41.917492711 2801.0 |
| 41.930622994 2805.0 |
| 41.943753276 2749.0 |
| 41.956883559 2833.0 |
| 41.970013842 2818.0 |
| 41.983144124 2677.0 |
| 41.996274407 2814.0 |
| 42.009404690 2778.0 |
| 42.022534972 2698.0 |
| 42.035665255 2706.0 |
| 42.048795538 2786.0 |
| 42.061925820 2703.0 |
| 42.075056103 2638.0 |
| 42.088186386 2803.0 |
| 42.101316668 2720.0 |
| 42.114446951 2775.0 |
| 42.127577234 2576.0 |
| 42.140707516 2573.0 |
| 42.153837799 2673.0 |
| 42.166968082 2702.0 |
| 42.180098364 2608.0 |
| 42.193228647 2559.0 |
| 42.206358930 2570.0 |
| 42.219489212 2565.0 |
| 42.232619495 2516.0 |
| 42.245749778 2577.0 |
| 42.258880060 2481.0 |
| 42.272010343 2492.0 |
| 42.285140626 2596.0 |
| 42.298270908 2580.0 |
| 42.311401191 2549.0 |
| 42.324531474 2587.0 |
| 42.337661757 2463.0 |
| 42.350792039 2520.0 |
| 42.363922322 2534.0 |
| 42.377052605 2380.0 |
| 42.390182887 2507.0 |
| 42.403313170 2372.0 |
| 42.416443453 2458.0 |
| 42.429573735 2492.0 |
| 42.442704018 2506.0 |
| 42.455834301 2492.0 |
| 42.468964583 2394.0 |
| 42.482094866 2438.0 |
| 42.495225149 2440.0 |
| 42.508355431 2437.0 |
| 42.521485714 2411.0 |
| 42.534615997 2437.0 |
| 42.547746279 2333.0 |
| 42.560876562 2360.0 |
| 42.574006845 2389.0 |
| 42.587137127 2333.0 |
| 42.600267410 2388.0 |
| 42.613397693 2425.0 |
| 42.626527975 2371.0 |
| 42.639658258 2318.0 |
| 42.652788541 2396.0 |
| 42.665918823 2406.0 |
| 42.679049106 2345.0 |
| 42.692179389 2302.0 |
| 42.705309671 2353.0 |
| 42.718439954 2404.0 |
| 42.731570237 2186.0 |
| 42.744700519 2355.0 |
| 42.757830802 2328.0 |
| 42.770961085 2309.0 |
| 42.784091368 2321.0 |
| 42.797221650 2316.0 |
| 42.810351933 2326.0 |
| 42.823482216 2285.0 |
| 42.836612498 2242.0 |
| 42.849742781 2256.0 |
| 42.862873064 2220.0 |
| 42.876003346 2200.0 |
| 42.889133629 2308.0 |
| 42.902263912 2262.0 |
| 42.915394194 2159.0 |
| 42.928524477 2167.0 |
| 42.941654760 2299.0 |
| 42.954785042 2267.0 |
| 42.967915325 2279.0 |
| 42.981045608 2267.0 |
| 42.994175890 2252.0 |
| 43.007306173 2231.0 |
| 43.020436456 2203.0 |
| 43.033566738 2181.0 |
| 43.046697021 2249.0 |
| 43.059827304 2193.0 |
| 43.072957586 2147.0 |
| 43.086087869 2229.0 |
| 43.099218152 2206.0 |
| 43.112348434 2179.0 |
| 43.125478717 2165.0 |
| 43.138609000 2124.0 |
| 43.151739282 2036.0 |
| 43.164869565 2119.0 |
| 43.177999848 2154.0 |
| 43.191130130 2232.0 |
| 43.204260413 2112.0 |
| 43.217390696 2131.0 |
| 43.230520979 2106.0 |
| 43.243651261 2071.0 |
| 43.256781544 2033.0 |
| 43.269911827 2151.0 |
| 43.283042109 2123.0 |
| 43.296172392 2044.0 |
| 43.309302675 2083.0 |
| 43.322432957 2038.0 |
| 43.335563240 2024.0 |
| 43.348693523 2072.0 |
| 43.361823805 2027.0 |
| 43.374954088 2066.0 |
| 43.388084371 1988.0 |
| 43.401214653 1981.0 |
| 43.414344936 2074.0 |
| 43.427475219 2014.0 |
| 43.440605501 2099.0 |
| 43.453735784 1965.0 |
| 43.466866067 2011.0 |
| 43.479996349 2014.0 |
| 43.493126632 2025.0 |
| 43.506256915 2004.0 |
| 43.519387197 2042.0 |
| 43.532517480 1946.0 |
| 43.545647763 2071.0 |
| 43.558778045 2030.0 |
| 43.571908328 2017.0 |
| 43.585038611 1922.0 |
| 43.598168893 1920.0 |
| 43.611299176 1975.0 |
| 43.624429459 1973.0 |
| 43.637559741 1990.0 |
| 43.650690024 1943.0 |
| 43.663820307 2026.0 |
| 43.676950590 1988.0 |
| 43.690080872 1997.0 |
| 43.703211155 1957.0 |
| 43.716341438 1940.0 |
| 43.729471720 1955.0 |
| 43.742602003 1910.0 |
| 43.755732286 1933.0 |
| 43.768862568 1953.0 |
| 43.781992851 1963.0 |
| 43.795123134 1974.0 |
| 43.808253416 1893.0 |
| 43.821383699 1980.0 |
| 43.834513982 1889.0 |
| 43.847644264 1919.0 |
| 43.860774547 1905.0 |
| 43.873904830 1986.0 |
| 43.887035112 1856.0 |
| 43.900165395 1945.0 |
| 43.913295678 1989.0 |
| 43.926425960 1858.0 |
| 43.939556243 1894.0 |
| 43.952686526 1884.0 |
| 43.965816808 1841.0 |
| 43.978947091 1866.0 |
| 43.992077374 1886.0 |
| 44.005207656 1918.0 |
| 44.018337939 1905.0 |
| 44.031468222 1911.0 |
| 44.044598504 1917.0 |
| 44.057728787 1997.0 |
| 44.070859070 1936.0 |
| 44.083989353 1899.0 |
| 44.097119635 1952.0 |
| 44.110249918 1949.0 |
| 44.123380201 1893.0 |
| 44.136510483 1915.0 |
| 44.149640766 1903.0 |
| 44.162771049 1882.0 |
| 44.175901331 1911.0 |
| 44.189031614 1901.0 |
| 44.202161897 1903.0 |
| 44.215292179 1857.0 |
| 44.228422462 1906.0 |
| 44.241552745 1930.0 |
| 44.254683027 1857.0 |
| 44.267813310 1846.0 |
| 44.280943593 1847.0 |
| 44.294073875 1865.0 |
| 44.307204158 1908.0 |
| 44.320334441 1868.0 |
| 44.333464723 1899.0 |
| 44.346595006 2006.0 |
| 44.359725289 1832.0 |
| 44.372855571 1870.0 |
| 44.385985854 1868.0 |
| 44.399116137 1860.0 |
| 44.412246419 1886.0 |
| 44.425376702 1871.0 |
| 44.438506985 1871.0 |
| 44.451637267 1797.0 |
| 44.464767550 1812.0 |
| 44.477897833 1889.0 |
| 44.491028115 1845.0 |
| 44.504158398 1853.0 |
| 44.517288681 1837.0 |
| 44.530418964 1840.0 |
| 44.543549246 1789.0 |
| 44.556679529 1875.0 |
| 44.569809812 1746.0 |
| 44.582940094 1841.0 |
| 44.596070377 1783.0 |
| 44.609200660 1836.0 |
| 44.622330942 1830.0 |
| 44.635461225 1857.0 |
| 44.648591508 1848.0 |
| 44.661721790 1723.0 |
| 44.674852073 1759.0 |
| 44.687982356 1858.0 |
| 44.701112638 1852.0 |
| 44.714242921 1813.0 |
| 44.727373204 1833.0 |
| 44.740503486 1844.0 |
| 44.753633769 1757.0 |
| 44.766764052 1823.0 |
| 44.779894334 1801.0 |
| 44.793024617 1813.0 |
| 44.806154900 1811.0 |
| 44.819285182 1830.0 |
| 44.832415465 1778.0 |
| 44.845545748 1858.0 |
| 44.858676030 1766.0 |
| 44.871806313 1812.0 |
| 44.884936596 1783.0 |
| 44.898066878 1804.0 |
| 44.911197161 1739.0 |
| 44.924327444 1752.0 |
| 44.937457726 1754.0 |
| 44.950588009 1742.0 |
| 44.963718292 1830.0 |
| 44.976848575 1774.0 |
| 44.989978857 1775.0 |
| 45.003109140 1719.0 |
| 45.016239423 1661.0 |
| 45.029369705 1850.0 |
| 45.042499988 1716.0 |
| 45.055630271 1787.0 |
| 45.068760553 1766.0 |
| 45.081890836 1779.0 |
| 45.095021119 1819.0 |
| 45.108151401 1758.0 |
| 45.121281684 1765.0 |
| 45.134411967 1711.0 |
| 45.147542249 1774.0 |
| 45.160672532 1821.0 |
| 45.173802815 1692.0 |
| 45.186933097 1766.0 |
| 45.200063380 1766.0 |
| 45.213193663 1666.0 |
| 45.226323945 1685.0 |
| 45.239454228 1774.0 |
| 45.252584511 1687.0 |
| 45.265714793 1708.0 |
| 45.278845076 1732.0 |
| 45.291975359 1792.0 |
| 45.305105641 1708.0 |
| 45.318235924 1717.0 |
| 45.331366207 1727.0 |
| 45.344496489 1763.0 |
| 45.357626772 1727.0 |
| 45.370757055 1702.0 |
| 45.383887337 1743.0 |
| 45.397017620 1765.0 |
| 45.410147903 1734.0 |
| 45.423278186 1709.0 |
| 45.436408468 1755.0 |
| 45.449538751 1723.0 |
| 45.462669034 1733.0 |
| 45.475799316 1680.0 |
| 45.488929599 1793.0 |
| 45.502059882 1665.0 |
| 45.515190164 1805.0 |
| 45.528320447 1791.0 |
| 45.541450730 1742.0 |
| 45.554581012 1727.0 |
| 45.567711295 1665.0 |
| 45.580841578 1767.0 |
| 45.593971860 1769.0 |
| 45.607102143 1668.0 |
| 45.620232426 1700.0 |
| 45.633362708 1686.0 |
| 45.646492991 1721.0 |
| 45.659623274 1681.0 |
| 45.672753556 1703.0 |
| 45.685883839 1716.0 |
| 45.699014122 1732.0 |
| 45.712144404 1638.0 |
| 45.725274687 1711.0 |
| 45.738404970 1718.0 |
| 45.751535252 1702.0 |
| 45.764665535 1709.0 |
| 45.777795818 1622.0 |
| 45.790926100 1717.0 |
| 45.804056383 1667.0 |
| 45.817186666 1621.0 |
| 45.830316948 1651.0 |
| 45.843447231 1671.0 |
| 45.856577514 1659.0 |
| 45.869707797 1648.0 |
| 45.882838079 1628.0 |
| 45.895968362 1621.0 |
| 45.909098645 1694.0 |
| 45.922228927 1692.0 |
| 45.935359210 1753.0 |
| 45.948489493 1625.0 |
| 45.961619775 1568.0 |
| 45.974750058 1600.0 |
| 45.987880341 1664.0 |
| 46.001010623 1704.0 |
| 46.014140906 1691.0 |
| 46.027271189 1703.0 |
| 46.040401471 1705.0 |
| 46.053531754 1636.0 |
| 46.066662037 1656.0 |
| 46.079792319 1621.0 |
| 46.092922602 1642.0 |
| 46.106052885 1707.0 |
| 46.119183167 1613.0 |
| 46.132313450 1603.0 |
| 46.145443733 1731.0 |
| 46.158574015 1717.0 |
| 46.171704298 1652.0 |
| 46.184834581 1750.0 |
| 46.197964863 1693.0 |
| 46.211095146 1631.0 |
| 46.224225429 1728.0 |
| 46.237355711 1777.0 |
| 46.250485994 1692.0 |
| 46.263616277 1637.0 |
| 46.276746559 1691.0 |
| 46.289876842 1628.0 |
| 46.303007125 1679.0 |
| 46.316137408 1645.0 |
| 46.329267690 1612.0 |
| 46.342397973 1641.0 |
| 46.355528256 1646.0 |
| 46.368658538 1650.0 |
| 46.381788821 1775.0 |
| 46.394919104 1672.0 |
| 46.408049386 1753.0 |
| 46.421179669 1654.0 |
| 46.434309952 1646.0 |
| 46.447440234 1665.0 |
| 46.460570517 1620.0 |
| 46.473700800 1704.0 |
| 46.486831082 1676.0 |
| 46.499961365 1724.0 |
| 46.513091648 1713.0 |
| 46.526221930 1664.0 |
| 46.539352213 1687.0 |
| 46.552482496 1663.0 |
| 46.565612778 1640.0 |
| 46.578743061 1721.0 |
| 46.591873344 1705.0 |
| 46.605003626 1751.0 |
| 46.618133909 1656.0 |
| 46.631264192 1655.0 |
| 46.644394474 1708.0 |
| 46.657524757 1597.0 |
| 46.670655040 1752.0 |
| 46.683785322 1679.0 |
| 46.696915605 1691.0 |
| 46.710045888 1689.0 |
| 46.723176171 1676.0 |
| 46.736306453 1671.0 |
| 46.749436736 1646.0 |
| 46.762567019 1665.0 |
| 46.775697301 1694.0 |
| 46.788827584 1704.0 |
| 46.801957867 1667.0 |
| 46.815088149 1595.0 |
| 46.828218432 1657.0 |
| 46.841348715 1669.0 |
| 46.854478997 1603.0 |
| 46.867609280 1683.0 |
| 46.880739563 1693.0 |
| 46.893869845 1684.0 |
| 46.907000128 1637.0 |
| 46.920130411 1605.0 |
| 46.933260693 1657.0 |
| 46.946390976 1633.0 |
| 46.959521259 1700.0 |
| 46.972651541 1724.0 |
| 46.985781824 1707.0 |
| 46.998912107 1674.0 |
| 47.012042389 1716.0 |
| 47.025172672 1708.0 |
| 47.038302955 1660.0 |
| 47.051433237 1682.0 |
| 47.064563520 1630.0 |
| 47.077693803 1734.0 |
| 47.090824085 1746.0 |
| 47.103954368 1636.0 |
| 47.117084651 1612.0 |
| 47.130214933 1674.0 |
| 47.143345216 1664.0 |
| 47.156475499 1591.0 |
| 47.169605782 1667.0 |
| 47.182736064 1666.0 |
| 47.195866347 1676.0 |
| 47.208996630 1601.0 |
| 47.222126912 1708.0 |
| 47.235257195 1660.0 |
| 47.248387478 1639.0 |
| 47.261517760 1697.0 |
| 47.274648043 1660.0 |
| 47.287778326 1631.0 |
| 47.300908608 1691.0 |
| 47.314038891 1692.0 |
| 47.327169174 1749.0 |
| 47.340299456 1651.0 |
| 47.353429739 1582.0 |
| 47.366560022 1682.0 |
| 47.379690304 1658.0 |
| 47.392820587 1706.0 |
| 47.405950870 1651.0 |
| 47.419081152 1654.0 |
| 47.432211435 1553.0 |
| 47.445341718 1549.0 |
| 47.458472000 1572.0 |
| 47.471602283 1653.0 |
| 47.484732566 1650.0 |
| 47.497862848 1682.0 |
| 47.510993131 1573.0 |
| 47.524123414 1641.0 |
| 47.537253696 1591.0 |
| 47.550383979 1612.0 |
| 47.563514262 1615.0 |
| 47.576644544 1558.0 |
| 47.589774827 1629.0 |
| 47.602905110 1609.0 |
| 47.616035393 1620.0 |
| 47.629165675 1573.0 |
| 47.642295958 1569.0 |
| 47.655426241 1581.0 |
| 47.668556523 1662.0 |
| 47.681686806 1608.0 |
| 47.694817089 1639.0 |
| 47.707947371 1664.0 |
| 47.721077654 1566.0 |
| 47.734207937 1586.0 |
| 47.747338219 1641.0 |
| 47.760468502 1627.0 |
| 47.773598785 1574.0 |
| 47.786729067 1551.0 |
| 47.799859350 1566.0 |
| 47.812989633 1607.0 |
| 47.826119915 1575.0 |
| 47.839250198 1575.0 |
| 47.852380481 1559.0 |
| 47.865510763 1599.0 |
| 47.878641046 1665.0 |
| 47.891771329 1555.0 |
| 47.904901611 1606.0 |
| 47.918031894 1657.0 |
| 47.931162177 1661.0 |
| 47.944292459 1584.0 |
| 47.957422742 1587.0 |
| 47.970553025 1607.0 |
| 47.983683307 1579.0 |
| 47.996813590 1568.0 |
| 48.009943873 1644.0 |
| 48.023074155 1569.0 |
| 48.036204438 1564.0 |
| 48.049334721 1547.0 |
| 48.062465004 1516.0 |
| 48.075595286 1570.0 |
| 48.088725569 1491.0 |
| 48.101855852 1516.0 |
| 48.114986134 1527.0 |
| 48.128116417 1629.0 |
| 48.141246700 1576.0 |
| 48.154376982 1560.0 |
| 48.167507265 1563.0 |
| 48.180637548 1583.0 |
| 48.193767830 1511.0 |
| 48.206898113 1600.0 |
| 48.220028396 1566.0 |
| 48.233158678 1545.0 |
| 48.246288961 1610.0 |
| 48.259419244 1549.0 |
| 48.272549526 1568.0 |
| 48.285679809 1611.0 |
| 48.298810092 1527.0 |
| 48.311940374 1567.0 |
| 48.325070657 1560.0 |
| 48.338200940 1559.0 |
| 48.351331222 1544.0 |
| 48.364461505 1522.0 |
| 48.377591788 1490.0 |
| 48.390722070 1554.0 |
| 48.403852353 1505.0 |
| 48.416982636 1491.0 |
| 48.430112918 1465.0 |
| 48.443243201 1531.0 |
| 48.456373484 1494.0 |
| 48.469503766 1472.0 |
| 48.482634049 1530.0 |
| 48.495764332 1491.0 |
| 48.508894615 1477.0 |
| 48.522024897 1513.0 |
| 48.535155180 1573.0 |
| 48.548285463 1515.0 |
| 48.561415745 1544.0 |
| 48.574546028 1502.0 |
| 48.587676311 1447.0 |
| 48.600806593 1505.0 |
| 48.613936876 1473.0 |
| 48.627067159 1523.0 |
| 48.640197441 1489.0 |
| 48.653327724 1465.0 |
| 48.666458007 1477.0 |
| 48.679588289 1493.0 |
| 48.692718572 1434.0 |
| 48.705848855 1492.0 |
| 48.718979137 1404.0 |
| 48.732109420 1504.0 |
| 48.745239703 1491.0 |
| 48.758369985 1458.0 |
| 48.771500268 1520.0 |
| 48.784630551 1463.0 |
| 48.797760833 1515.0 |
| 48.810891116 1518.0 |
| 48.824021399 1441.0 |
| 48.837151681 1467.0 |
| 48.850281964 1405.0 |
| 48.863412247 1367.0 |
| 48.876542529 1372.0 |
| 48.889672812 1419.0 |
| 48.902803095 1473.0 |
| 48.915933378 1420.0 |
| 48.929063660 1415.0 |
| 48.942193943 1375.0 |
| 48.955324226 1443.0 |
| 48.968454508 1425.0 |
| 48.981584791 1497.0 |
| 48.994715074 1415.0 |
| 49.007845356 1373.0 |
| 49.020975639 1463.0 |
| 49.034105922 1424.0 |
| 49.047236204 1458.0 |
| 49.060366487 1435.0 |
| 49.073496770 1450.0 |
| 49.086627052 1450.0 |
| 49.099757335 1385.0 |
| 49.112887618 1381.0 |
| 49.126017900 1461.0 |
| 49.139148183 1374.0 |
| 49.152278466 1388.0 |
| 49.165408748 1393.0 |
| 49.178539031 1434.0 |
| 49.191669314 1365.0 |
| 49.204799596 1320.0 |
| 49.217929879 1360.0 |
| 49.231060162 1441.0 |
| 49.244190444 1338.0 |
| 49.257320727 1428.0 |
| 49.270451010 1396.0 |
| 49.283581292 1368.0 |
| 49.296711575 1364.0 |
| 49.309841858 1398.0 |
| 49.322972140 1348.0 |
| 49.336102423 1369.0 |
| 49.349232706 1353.0 |
| 49.362362989 1369.0 |
| 49.375493271 1370.0 |
| 49.388623554 1421.0 |
| 49.401753837 1378.0 |
| 49.414884119 1291.0 |
| 49.428014402 1296.0 |
| 49.441144685 1354.0 |
| 49.454274967 1346.0 |
| 49.467405250 1366.0 |
| 49.480535533 1327.0 |
| 49.493665815 1340.0 |
| 49.506796098 1358.0 |
| 49.519926381 1360.0 |
| 49.533056663 1349.0 |
| 49.546186946 1314.0 |
| 49.559317229 1333.0 |
| 49.572447511 1328.0 |
| 49.585577794 1243.0 |
| 49.598708077 1356.0 |
| 49.611838359 1336.0 |
| 49.624968642 1298.0 |
| 49.638098925 1309.0 |
| 49.651229207 1322.0 |
| 49.664359490 1352.0 |
| 49.677489773 1345.0 |
| 49.690620055 1343.0 |
| 49.703750338 1346.0 |
| 49.716880621 1313.0 |
| 49.730010903 1325.0 |
| 49.743141186 1321.0 |
| 49.756271469 1285.0 |
| 49.769401751 1365.0 |
| 49.782532034 1270.0 |
| 49.795662317 1336.0 |
| 49.808792600 1289.0 |
| 49.821922882 1296.0 |
| 49.835053165 1287.0 |
| 49.848183448 1303.0 |
| 49.861313730 1324.0 |
| 49.874444013 1363.0 |
| 49.887574296 1297.0 |
| 49.900704578 1289.0 |
| 49.913834861 1264.0 |
| 49.926965144 1275.0 |
| 49.940095426 1400.0 |
| 49.953225709 1291.0 |
| 49.966355992 1248.0 |
| 49.979486274 1289.0 |
| 49.992616557 1336.0 |
| 50.005746840 1298.0 |
| 50.018877122 1242.0 |
| 50.032007405 1256.0 |
| 50.045137688 1200.0 |
| 50.058267970 1236.0 |
| 50.071398253 1337.0 |
| 50.084528536 1231.0 |
| 50.097658818 1291.0 |
| 50.110789101 1298.0 |
| 50.123919384 1275.0 |
| 50.137049666 1291.0 |
| 50.150179949 1258.0 |
| 50.163310232 1251.0 |
| 50.176440514 1337.0 |
| 50.189570797 1254.0 |
| 50.202701080 1304.0 |
| 50.215831362 1268.0 |
| 50.228961645 1286.0 |
| 50.242091928 1230.0 |
| 50.255222211 1239.0 |
| 50.268352493 1216.0 |
| 50.281482776 1238.0 |
| 50.294613059 1279.0 |
| 50.307743341 1341.0 |
| 50.320873624 1301.0 |
| 50.334003907 1239.0 |
| 50.347134189 1234.0 |
| 50.360264472 1257.0 |
| 50.373394755 1267.0 |
| 50.386525037 1271.0 |
| 50.399655320 1225.0 |
| 50.412785603 1202.0 |
| 50.425915885 1219.0 |
| 50.439046168 1274.0 |
| 50.452176451 1243.0 |
| 50.465306733 1252.0 |
| 50.478437016 1233.0 |
| 50.491567299 1166.0 |
| 50.504697581 1243.0 |
| 50.517827864 1291.0 |
| 50.530958147 1243.0 |
| 50.544088429 1230.0 |
| 50.557218712 1194.0 |
| 50.570348995 1228.0 |
| 50.583479277 1283.0 |
| 50.596609560 1277.0 |
| 50.609739843 1221.0 |
| 50.622870125 1211.0 |
| 50.636000408 1219.0 |
| 50.649130691 1239.0 |
| 50.662260973 1260.0 |
| 50.675391256 1243.0 |
| 50.688521539 1225.0 |
| 50.701651822 1197.0 |
| 50.714782104 1205.0 |
| 50.727912387 1265.0 |
| 50.741042670 1210.0 |
| 50.754172952 1195.0 |
| 50.767303235 1215.0 |
| 50.780433518 1159.0 |
| 50.793563800 1170.0 |
| 50.806694083 1193.0 |
| 50.819824366 1229.0 |
| 50.832954648 1271.0 |
| 50.846084931 1202.0 |
| 50.859215214 1233.0 |
| 50.872345496 1230.0 |
| 50.885475779 1265.0 |
| 50.898606062 1215.0 |
| 50.911736344 1234.0 |
| 50.924866627 1224.0 |
| 50.937996910 1167.0 |
| 50.951127192 1178.0 |
| 50.964257475 1244.0 |
| 50.977387758 1226.0 |
| 50.990518040 1150.0 |
| 51.003648323 1200.0 |
| 51.016778606 1238.0 |
| 51.029908888 1154.0 |
| 51.043039171 1239.0 |
| 51.056169454 1272.0 |
| 51.069299736 1196.0 |
| 51.082430019 1290.0 |
| 51.095560302 1206.0 |
| 51.108690584 1191.0 |
| 51.121820867 1205.0 |
| 51.134951150 1225.0 |
| 51.148081433 1135.0 |
| 51.161211715 1259.0 |
| 51.174341998 1219.0 |
| 51.187472281 1197.0 |
| 51.200602563 1145.0 |
| 51.213732846 1190.0 |
| 51.226863129 1179.0 |
| 51.239993411 1251.0 |
| 51.253123694 1223.0 |
| 51.266253977 1207.0 |
| 51.279384259 1144.0 |
| 51.292514542 1193.0 |
| 51.305644825 1235.0 |
| 51.318775107 1158.0 |
| 51.331905390 1158.0 |
| 51.345035673 1180.0 |
| 51.358165955 1187.0 |
| 51.371296238 1199.0 |
| 51.384426521 1256.0 |
| 51.397556803 1141.0 |
| 51.410687086 1168.0 |
| 51.423817369 1150.0 |
| 51.436947651 1222.0 |
| 51.450077934 1184.0 |
| 51.463208217 1158.0 |
| 51.476338499 1159.0 |
| 51.489468782 1169.0 |
| 51.502599065 1242.0 |
| 51.515729347 1262.0 |
| 51.528859630 1208.0 |
| 51.541989913 1221.0 |
| 51.555120196 1244.0 |
| 51.568250478 1192.0 |
| 51.581380761 1151.0 |
| 51.594511044 1176.0 |
| 51.607641326 1170.0 |
| 51.620771609 1164.0 |
| 51.633901892 1177.0 |
| 51.647032174 1268.0 |
| 51.660162457 1275.0 |
| 51.673292740 1168.0 |
| 51.686423022 1180.0 |
| 51.699553305 1149.0 |
| 51.712683588 1102.0 |
| 51.725813870 1155.0 |
| 51.738944153 1212.0 |
| 51.752074436 1192.0 |
| 51.765204718 1221.0 |
| 51.778335001 1224.0 |
| 51.791465284 1215.0 |
| 51.804595566 1148.0 |
| 51.817725849 1125.0 |
| 51.830856132 1214.0 |
| 51.843986414 1127.0 |
| 51.857116697 1196.0 |
| 51.870246980 1199.0 |
| 51.883377262 1171.0 |
| 51.896507545 1214.0 |
| 51.909637828 1192.0 |
| 51.922768110 1141.0 |
| 51.935898393 1168.0 |
| 51.949028676 1170.0 |
| 51.962158958 1226.0 |
| 51.975289241 1170.0 |
| 51.988419524 1222.0 |
| 52.001549807 1152.0 |
| 52.014680089 1173.0 |
| 52.027810372 1164.0 |
| 52.040940655 1151.0 |
| 52.054070937 1270.0 |
| 52.067201220 1130.0 |
| 52.080331503 1212.0 |
| 52.093461785 1119.0 |
| 52.106592068 1167.0 |
| 52.119722351 1243.0 |
| 52.132852633 1235.0 |
| 52.145982916 1102.0 |
| 52.159113199 1181.0 |
| 52.172243481 1161.0 |
| 52.185373764 1086.0 |
| 52.198504047 1194.0 |
| 52.211634329 1207.0 |
| 52.224764612 1149.0 |
| 52.237894895 1143.0 |
| 52.251025177 1188.0 |
| 52.264155460 1131.0 |
| 52.277285743 1107.0 |
| 52.290416025 1138.0 |
| 52.303546308 1148.0 |
| 52.316676591 1224.0 |
| 52.329806873 1207.0 |
| 52.342937156 1169.0 |
| 52.356067439 1146.0 |
| 52.369197721 1182.0 |
| 52.382328004 1144.0 |
| 52.395458287 1189.0 |
| 52.408588569 1166.0 |
| 52.421718852 1144.0 |
| 52.434849135 1095.0 |
| 52.447979418 1124.0 |
| 52.461109700 1143.0 |
| 52.474239983 1173.0 |
| 52.487370266 1169.0 |
| 52.500500548 1120.0 |
| 52.513630831 1176.0 |
| 52.526761114 1162.0 |
| 52.539891396 1192.0 |
| 52.553021679 1227.0 |
| 52.566151962 1095.0 |
| 52.579282244 1193.0 |
| 52.592412527 1139.0 |
| 52.605542810 1214.0 |
| 52.618673092 1179.0 |
| 52.631803375 1131.0 |
| 52.644933658 1111.0 |
| 52.658063940 1140.0 |
| 52.671194223 1191.0 |
| 52.684324506 1179.0 |
| 52.697454788 1153.0 |
| 52.710585071 1170.0 |
| 52.723715354 1162.0 |
| 52.736845636 1187.0 |
| 52.749975919 1127.0 |
| 52.763106202 1131.0 |
| 52.776236484 1188.0 |
| 52.789366767 1158.0 |
| 52.802497050 1161.0 |
| 52.815627332 1161.0 |
| 52.828757615 1194.0 |
| 52.841887898 1134.0 |
| 52.855018180 1135.0 |
| 52.868148463 1205.0 |
| 52.881278746 1163.0 |
| 52.894409029 1164.0 |
| 52.907539311 1079.0 |
| 52.920669594 1129.0 |
| 52.933799877 1202.0 |
| 52.946930159 1149.0 |
| 52.960060442 1159.0 |
| 52.973190725 1110.0 |
| 52.986321007 1120.0 |
| 52.999451290 1122.0 |
| 53.012581573 1077.0 |
| 53.025711855 1085.0 |
| 53.038842138 1145.0 |
| 53.051972421 1109.0 |
| 53.065102703 1141.0 |
| 53.078232986 1133.0 |
| 53.091363269 1065.0 |
| 53.104493551 1118.0 |
| 53.117623834 1086.0 |
| 53.130754117 1045.0 |
| 53.143884399 1109.0 |
| 53.157014682 1137.0 |
| 53.170144965 1116.0 |
| 53.183275247 1085.0 |
| 53.196405530 1170.0 |
| 53.209535813 1127.0 |
| 53.222666095 1138.0 |
| 53.235796378 1197.0 |
| 53.248926661 1117.0 |
| 53.262056943 1135.0 |
| 53.275187226 1139.0 |
| 53.288317509 1122.0 |
| 53.301447791 1148.0 |
| 53.314578074 1137.0 |
| 53.327708357 1125.0 |
| 53.340838640 1127.0 |
| 53.353968922 1215.0 |
| 53.367099205 1197.0 |
| 53.380229488 1163.0 |
| 53.393359770 1035.0 |
| 53.406490053 1153.0 |
| 53.419620336 1183.0 |
| 53.432750618 1138.0 |
| 53.445880901 1189.0 |
| 53.459011184 1124.0 |
| 53.472141466 1090.0 |
| 53.485271749 1158.0 |
| 53.498402032 1066.0 |
| 53.511532314 1124.0 |
| 53.524662597 1114.0 |
| 53.537792880 1071.0 |
| 53.550923162 1094.0 |
| 53.564053445 1113.0 |
| 53.577183728 1120.0 |
| 53.590314010 1128.0 |
| 53.603444293 1164.0 |
| 53.616574576 1129.0 |
| 53.629704858 1063.0 |
| 53.642835141 1106.0 |
| 53.655965424 1122.0 |
| 53.669095706 1152.0 |
| 53.682225989 1172.0 |
| 53.695356272 1116.0 |
| 53.708486554 1155.0 |
| 53.721616837 1178.0 |
| 53.734747120 1142.0 |
| 53.747877403 1108.0 |
| 53.761007685 1133.0 |
| 53.774137968 1130.0 |
| 53.787268251 1140.0 |
| 53.800398533 1167.0 |
| 53.813528816 1119.0 |
| 53.826659099 1126.0 |
| 53.839789381 1144.0 |
| 53.852919664 1119.0 |
| 53.866049947 1051.0 |
| 53.879180229 1196.0 |
| 53.892310512 1173.0 |
| 53.905440795 1123.0 |
| 53.918571077 1170.0 |
| 53.931701360 1124.0 |
| 53.944831643 1123.0 |
| 53.957961925 1132.0 |
| 53.971092208 1064.0 |
| 53.984222491 1134.0 |
| 53.997352773 1135.0 |
| 54.010483056 1164.0 |
| 54.023613339 1172.0 |
| 54.036743621 1170.0 |
| 54.049873904 1179.0 |
| 54.063004187 1123.0 |
| 54.076134469 1159.0 |
| 54.089264752 1217.0 |
| 54.102395035 1175.0 |
| 54.115525317 1171.0 |
| 54.128655600 1185.0 |
| 54.141785883 1153.0 |
| 54.154916165 1141.0 |
| 54.168046448 1081.0 |
| 54.181176731 1162.0 |
| 54.194307014 1131.0 |
| 54.207437296 1119.0 |
| 54.220567579 1097.0 |
| 54.233697862 1153.0 |
| 54.246828144 1127.0 |
| 54.259958427 1215.0 |
| 54.273088710 1098.0 |
| 54.286218992 1119.0 |
| 54.299349275 1149.0 |
| 54.312479558 1128.0 |
| 54.325609840 1169.0 |
| 54.338740123 1141.0 |
| 54.351870406 1121.0 |
| 54.365000688 1163.0 |
| 54.378130971 1117.0 |
| 54.391261254 1168.0 |
| 54.404391536 1173.0 |
| 54.417521819 1124.0 |
| 54.430652102 1119.0 |
| 54.443782384 1134.0 |
| 54.456912667 1161.0 |
| 54.470042950 1106.0 |
| 54.483173232 1136.0 |
| 54.496303515 1104.0 |
| 54.509433798 1145.0 |
| 54.522564080 1101.0 |
| 54.535694363 1044.0 |
| 54.548824646 1134.0 |
| 54.561954928 1096.0 |
| 54.575085211 1108.0 |
| 54.588215494 1034.0 |
| 54.601345776 1100.0 |
| 54.614476059 1161.0 |
| 54.627606342 1108.0 |
| 54.640736625 1122.0 |
| 54.653866907 1079.0 |
| 54.666997190 1092.0 |
| 54.680127473 1106.0 |
| 54.693257755 1088.0 |
| 54.706388038 1130.0 |
| 54.719518321 1126.0 |
| 54.732648603 1080.0 |
| 54.745778886 1050.0 |
| 54.758909169 1178.0 |
| 54.772039451 1081.0 |
| 54.785169734 1086.0 |
| 54.798300017 1076.0 |
| 54.811430299 1133.0 |
| 54.824560582 1021.0 |
| 54.837690865 1102.0 |
| 54.850821147 1155.0 |
| 54.863951430 1141.0 |
| 54.877081713 1054.0 |
| 54.890211995 1037.0 |
| 54.903342278 1089.0 |
| 54.916472561 1092.0 |
| 54.929602843 1120.0 |
| 54.942733126 1064.0 |
| 54.955863409 1121.0 |
| 54.968993691 1124.0 |
| 54.982123974 1070.0 |
| 54.995254257 1115.0 |
| 55.008384539 1000.0 |
| 55.021514822 1133.0 |
| 55.034645105 1118.0 |
| 55.047775387 1143.0 |
| 55.060905670 1152.0 |
| 55.074035953 1049.0 |
| 55.087166236 1122.0 |
| 55.100296518 1075.0 |
| 55.113426801 1081.0 |
| 55.126557084 1053.0 |
| 55.139687366 1047.0 |
| 55.152817649 1054.0 |
| 55.165947932 1129.0 |
| 55.179078214 1093.0 |
| 55.192208497 1076.0 |
| 55.205338780 960.0 |
| 55.218469062 1079.0 |
| 55.231599345 1121.0 |
| 55.244729628 1103.0 |
| 55.257859910 1069.0 |
| 55.270990193 1041.0 |
| 55.284120476 1014.0 |
| 55.297250758 1130.0 |
| 55.310381041 1000.0 |
| 55.323511324 1090.0 |
| 55.336641606 1058.0 |
| 55.349771889 1063.0 |
| 55.362902172 1089.0 |
| 55.376032454 1069.0 |
| 55.389162737 1081.0 |
| 55.402293020 1087.0 |
| 55.415423302 1070.0 |
| 55.428553585 1085.0 |
| 55.441683868 1060.0 |
| 55.454814150 1079.0 |
| 55.467944433 1057.0 |
| 55.481074716 1136.0 |
| 55.494204998 1090.0 |
| 55.507335281 1079.0 |
| 55.520465564 1072.0 |
| 55.533595847 1034.0 |
| 55.546726129 1088.0 |
| 55.559856412 998.0 |
| 55.572986695 1026.0 |
| 55.586116977 1068.0 |
| 55.599247260 1053.0 |
| 55.612377543 1048.0 |
| 55.625507825 1022.0 |
| 55.638638108 1033.0 |
| 55.651768391 1061.0 |
| 55.664898673 1090.0 |
| 55.678028956 1125.0 |
| 55.691159239 1083.0 |
| 55.704289521 1102.0 |
| 55.717419804 1057.0 |
| 55.730550087 1088.0 |
| 55.743680369 1059.0 |
| 55.756810652 1004.0 |
| 55.769940935 989.0 |
| 55.783071217 1022.0 |
| 55.796201500 1077.0 |
| 55.809331783 1088.0 |
| 55.822462065 1026.0 |
| 55.835592348 1059.0 |
| 55.848722631 1100.0 |
| 55.861852913 1062.0 |
| 55.874983196 1064.0 |
| 55.888113479 1054.0 |
| 55.901243761 1065.0 |
| 55.914374044 1128.0 |
| 55.927504327 1019.0 |
| 55.940634609 1070.0 |
| 55.953764892 1053.0 |
| 55.966895175 1022.0 |
| 55.980025458 1055.0 |
| 55.993155740 1091.0 |
| 56.006286023 1066.0 |
| 56.019416306 1071.0 |
| 56.032546588 1057.0 |
| 56.045676871 1002.0 |
| 56.058807154 1073.0 |
| 56.071937436 1129.0 |
| 56.085067719 1082.0 |
| 56.098198002 1073.0 |
| 56.111328284 1014.0 |
| 56.124458567 1027.0 |
| 56.137588850 1131.0 |
| 56.150719132 1097.0 |
| 56.163849415 1116.0 |
| 56.176979698 1049.0 |
| 56.190109980 1084.0 |
| 56.203240263 1042.0 |
| 56.216370546 961.0 |
| 56.229500828 1043.0 |
| 56.242631111 1050.0 |
| 56.255761394 1085.0 |
| 56.268891676 1050.0 |
| 56.282021959 988.0 |
| 56.295152242 1049.0 |
| 56.308282524 1044.0 |
| 56.321412807 988.0 |
| 56.334543090 1006.0 |
| 56.347673372 1130.0 |
| 56.360803655 999.0 |
| 56.373933938 1058.0 |
| 56.387064221 1103.0 |
| 56.400194503 1024.0 |
| 56.413324786 1074.0 |
| 56.426455069 1041.0 |
| 56.439585351 1063.0 |
| 56.452715634 1048.0 |
| 56.465845917 1047.0 |
| 56.478976199 1020.0 |
| 56.492106482 1084.0 |
| 56.505236765 1012.0 |
| 56.518367047 1051.0 |
| 56.531497330 1015.0 |
| 56.544627613 1042.0 |
| 56.557757895 1059.0 |
| 56.570888178 1009.0 |
| 56.584018461 1057.0 |
| 56.597148743 1065.0 |
| 56.610279026 1015.0 |
| 56.623409309 992.0 |
| 56.636539591 1066.0 |
| 56.649669874 1081.0 |
| 56.662800157 1009.0 |
| 56.675930439 1016.0 |
| 56.689060722 1020.0 |
| 56.702191005 983.0 |
| 56.715321287 1024.0 |
| 56.728451570 1067.0 |
| 56.741581853 1026.0 |
| 56.754712135 1016.0 |
| 56.767842418 958.0 |
| 56.780972701 1053.0 |
| 56.794102983 1010.0 |
| 56.807233266 1043.0 |
| 56.820363549 1001.0 |
| 56.833493832 1032.0 |
| 56.846624114 953.0 |
| 56.859754397 958.0 |
| 56.872884680 1052.0 |
| 56.886014962 1106.0 |
| 56.899145245 1066.0 |
| 56.912275528 1009.0 |
| 56.925405810 1043.0 |
| 56.938536093 1041.0 |
| 56.951666376 1044.0 |
| 56.964796658 1058.0 |
| 56.977926941 1020.0 |
| 56.991057224 1017.0 |
| 57.004187506 1017.0 |
| 57.017317789 1080.0 |
| 57.030448072 981.0 |
| 57.043578354 1053.0 |
| 57.056708637 1011.0 |
| 57.069838920 1029.0 |
| 57.082969202 994.0 |
| 57.096099485 1030.0 |
| 57.109229768 995.0 |
| 57.122360050 1065.0 |
| 57.135490333 1050.0 |
| 57.148620616 962.0 |
| 57.161750898 1016.0 |
| 57.174881181 978.0 |
| 57.188011464 1008.0 |
| 57.201141746 1015.0 |
| 57.214272029 1025.0 |
| 57.227402312 1081.0 |
| 57.240532594 1067.0 |
| 57.253662877 1045.0 |
| 57.266793160 1079.0 |
| 57.279923443 1032.0 |
| 57.293053725 1068.0 |
| 57.306184008 1008.0 |
| 57.319314291 1110.0 |
| 57.332444573 1012.0 |
| 57.345574856 1006.0 |
| 57.358705139 982.0 |
| 57.371835421 1019.0 |
| 57.384965704 979.0 |
| 57.398095987 986.0 |
| 57.411226269 1021.0 |
| 57.424356552 1036.0 |
| 57.437486835 992.0 |
| 57.450617117 1040.0 |
| 57.463747400 1042.0 |
| 57.476877683 1085.0 |
| 57.490007965 982.0 |
| 57.503138248 1023.0 |
| 57.516268531 1003.0 |
| 57.529398813 1072.0 |
| 57.542529096 1055.0 |
| 57.555659379 1057.0 |
| 57.568789661 1077.0 |
| 57.581919944 1085.0 |
| 57.595050227 1001.0 |
| 57.608180509 1033.0 |
| 57.621310792 1024.0 |
| 57.634441075 1082.0 |
| 57.647571357 1042.0 |
| 57.660701640 1024.0 |
| 57.673831923 1062.0 |
| 57.686962205 988.0 |
| 57.700092488 1052.0 |
| 57.713222771 1067.0 |
| 57.726353054 1092.0 |
| 57.739483336 1013.0 |
| 57.752613619 1036.0 |
| 57.765743902 1082.0 |
| 57.778874184 1089.0 |
| 57.792004467 1128.0 |
| 57.805134750 1019.0 |
| 57.818265032 1050.0 |
| 57.831395315 1134.0 |
| 57.844525598 1073.0 |
| 57.857655880 1088.0 |
| 57.870786163 1067.0 |
| 57.883916446 1012.0 |
| 57.897046728 1012.0 |
| 57.910177011 1012.0 |
| 57.923307294 1063.0 |
| 57.936437576 1004.0 |
| 57.949567859 1017.0 |
| 57.962698142 1013.0 |
| 57.975828424 1007.0 |
| 57.988958707 1000.0 |
| 58.002088990 976.0 |
| 58.015219272 971.0 |
| 58.028349555 1022.0 |
| 58.041479838 1039.0 |
| 58.054610120 1044.0 |
| 58.067740403 995.0 |
| 58.080870686 1013.0 |
| 58.094000968 1003.0 |
| 58.107131251 1008.0 |
| 58.120261534 1018.0 |
| 58.133391816 968.0 |
| 58.146522099 996.0 |
| 58.159652382 996.0 |
| 58.172782665 1035.0 |
| 58.185912947 973.0 |
| 58.199043230 1037.0 |
| 58.212173513 956.0 |
| 58.225303795 1000.0 |
| 58.238434078 1020.0 |
| 58.251564361 962.0 |
| 58.264694643 978.0 |
| 58.277824926 981.0 |
| 58.290955209 1034.0 |
| 58.304085491 989.0 |
| 58.317215774 934.0 |
| 58.330346057 1033.0 |
| 58.343476339 957.0 |
| 58.356606622 982.0 |
| 58.369736905 975.0 |
| 58.382867187 978.0 |
| 58.395997470 957.0 |
| 58.409127753 956.0 |
| 58.422258035 992.0 |
| 58.435388318 894.0 |
| 58.448518601 1011.0 |
| 58.461648883 1001.0 |
| 58.474779166 989.0 |
| 58.487909449 941.0 |
| 58.501039731 1032.0 |
| 58.514170014 994.0 |
| 58.527300297 965.0 |
| 58.540430579 914.0 |
| 58.553560862 976.0 |
| 58.566691145 969.0 |
| 58.579821428 999.0 |
| 58.592951710 1061.0 |
| 58.606081993 964.0 |
| 58.619212276 985.0 |
| 58.632342558 983.0 |
| 58.645472841 955.0 |
| 58.658603124 986.0 |
| 58.671733406 917.0 |
| 58.684863689 969.0 |
| 58.697993972 983.0 |
| 58.711124254 987.0 |
| 58.724254537 980.0 |
| 58.737384820 1019.0 |
| 58.750515102 943.0 |
| 58.763645385 911.0 |
| 58.776775668 942.0 |
| 58.789905950 999.0 |
| 58.803036233 914.0 |
| 58.816166516 927.0 |
| 58.829296798 960.0 |
| 58.842427081 953.0 |
| 58.855557364 979.0 |
| 58.868687646 981.0 |
| 58.881817929 981.0 |
| 58.894948212 938.0 |
| 58.908078494 1010.0 |
| 58.921208777 981.0 |
| 58.934339060 1014.0 |
| 58.947469342 1034.0 |
| 58.960599625 1022.0 |
| 58.973729908 913.0 |
| 58.986860190 964.0 |
| 58.999990473 955.0 |
| 59.013120756 945.0 |
| 59.026251039 973.0 |
| 59.039381321 964.0 |
| 59.052511604 936.0 |
| 59.065641887 946.0 |
| 59.078772169 889.0 |
| 59.091902452 913.0 |
| 59.105032735 939.0 |
| 59.118163017 928.0 |
| 59.131293300 1001.0 |
| 59.144423583 980.0 |
| 59.157553865 938.0 |
| 59.170684148 968.0 |
| 59.183814431 949.0 |
| 59.196944713 918.0 |
| 59.210074996 918.0 |
| 59.223205279 944.0 |
| 59.236335561 954.0 |
| 59.249465844 959.0 |
| 59.262596127 944.0 |
| 59.275726409 941.0 |
| 59.288856692 959.0 |
| 59.301986975 926.0 |
| 59.315117257 937.0 |
| 59.328247540 998.0 |
| 59.341377823 938.0 |
| 59.354508105 939.0 |
| 59.367638388 899.0 |
| 59.380768671 936.0 |
| 59.393898953 979.0 |
| 59.407029236 924.0 |
| 59.420159519 1007.0 |
| 59.433289801 964.0 |
| 59.446420084 991.0 |
| 59.459550367 966.0 |
| 59.472680650 984.0 |
| 59.485810932 1009.0 |
| 59.498941215 919.0 |
| 59.512071498 935.0 |
| 59.525201780 901.0 |
| 59.538332063 925.0 |
| 59.551462346 930.0 |
| 59.564592628 996.0 |
| 59.577722911 944.0 |
| 59.590853194 976.0 |
| 59.603983476 977.0 |
| 59.617113759 926.0 |
| 59.630244042 965.0 |
| 59.643374324 896.0 |
| 59.656504607 957.0 |
| 59.669634890 912.0 |
| 59.682765172 956.0 |
| 59.695895455 949.0 |
| 59.709025738 949.0 |
| 59.722156020 935.0 |
| 59.735286303 960.0 |
| 59.748416586 915.0 |
| 59.761546868 937.0 |
| 59.774677151 962.0 |
| 59.787807434 962.0 |
| 59.800937716 951.0 |
| 59.814067999 947.0 |
| 59.827198282 944.0 |
| 59.840328564 967.0 |
| 59.853458847 979.0 |
| 59.866589130 956.0 |
| 59.879719412 949.0 |
| 59.892849695 959.0 |
| 59.905979978 1005.0 |
| 59.919110261 917.0 |
| 59.932240543 942.0 |
| 59.945370826 978.0 |
| 59.958501109 911.0 |
| 59.971631391 947.0 |
| 59.984761674 900.0 |
| 59.997891957 927.0 |
| 60.011022239 982.0 |
| 60.024152522 915.0 |
| 60.037282805 945.0 |
| 60.050413087 966.0 |
| 60.063543370 985.0 |
| 60.076673653 927.0 |
| 60.089803935 988.0 |
| 60.102934218 884.0 |
| 60.116064501 923.0 |
| 60.129194783 950.0 |
| 60.142325066 928.0 |
| 60.155455349 945.0 |
| 60.168585631 938.0 |
| 60.181715914 963.0 |
| 60.194846197 934.0 |
| 60.207976479 967.0 |
| 60.221106762 919.0 |
| 60.234237045 957.0 |
| 60.247367327 965.0 |
| 60.260497610 925.0 |
| 60.273627893 994.0 |
| 60.286758175 940.0 |
| 60.299888458 913.0 |
| 60.313018741 1010.0 |
| 60.326149023 935.0 |
| 60.339279306 901.0 |
| 60.352409589 983.0 |
| 60.365539872 998.0 |
| 60.378670154 927.0 |
| 60.391800437 970.0 |
| 60.404930720 953.0 |
| 60.418061002 894.0 |
| 60.431191285 942.0 |
| 60.444321568 982.0 |
| 60.457451850 916.0 |
| 60.470582133 937.0 |
| 60.483712416 932.0 |
| 60.496842698 968.0 |
| 60.509972981 931.0 |
| 60.523103264 995.0 |
| 60.536233546 909.0 |
| 60.549363829 950.0 |
| 60.562494112 948.0 |
| 60.575624394 904.0 |
| 60.588754677 932.0 |
| 60.601884960 882.0 |
| 60.615015242 971.0 |
| 60.628145525 914.0 |
| 60.641275808 922.0 |
| 60.654406090 951.0 |
| 60.667536373 898.0 |
| 60.680666656 976.0 |
| 60.693796938 974.0 |
| 60.706927221 930.0 |
| 60.720057504 937.0 |
| 60.733187786 919.0 |
| 60.746318069 943.0 |
| 60.759448352 970.0 |
| 60.772578634 970.0 |
| 60.785708917 973.0 |
| 60.798839200 960.0 |
| 60.811969483 938.0 |
| 60.825099765 926.0 |
| 60.838230048 949.0 |
| 60.851360331 970.0 |
| 60.864490613 890.0 |
| 60.877620896 953.0 |
| 60.890751179 923.0 |
| 60.903881461 993.0 |
| 60.917011744 899.0 |
| 60.930142027 955.0 |
| 60.943272309 992.0 |
| 60.956402592 948.0 |
| 60.969532875 962.0 |
| 60.982663157 904.0 |
| 60.995793440 941.0 |
| 61.008923723 946.0 |
| 61.022054005 942.0 |
| 61.035184288 934.0 |
| 61.048314571 943.0 |
| 61.061444853 984.0 |
| 61.074575136 976.0 |
| 61.087705419 892.0 |
| 61.100835701 886.0 |
| 61.113965984 928.0 |
| 61.127096267 919.0 |
| 61.140226549 936.0 |
| 61.153356832 1011.0 |
| 61.166487115 904.0 |
| 61.179617397 939.0 |
| 61.192747680 902.0 |
| 61.205877963 948.0 |
| 61.219008246 993.0 |
| 61.232138528 871.0 |
| 61.245268811 960.0 |
| 61.258399094 871.0 |
| 61.271529376 974.0 |
| 61.284659659 880.0 |
| 61.297789942 969.0 |
| 61.310920224 976.0 |
| 61.324050507 912.0 |
| 61.337180790 939.0 |
| 61.350311072 894.0 |
| 61.363441355 946.0 |
| 61.376571638 932.0 |
| 61.389701920 922.0 |
| 61.402832203 923.0 |
| 61.415962486 1007.0 |
| 61.429092768 932.0 |
| 61.442223051 995.0 |
| 61.455353334 1004.0 |
| 61.468483616 910.0 |
| 61.481613899 938.0 |
| 61.494744182 917.0 |
| 61.507874464 883.0 |
| 61.521004747 910.0 |
| 61.534135030 879.0 |
| 61.547265312 908.0 |
| 61.560395595 937.0 |
| 61.573525878 917.0 |
| 61.586656160 991.0 |
| 61.599786443 875.0 |
| 61.612916726 964.0 |
| 61.626047008 930.0 |
| 61.639177291 908.0 |
| 61.652307574 891.0 |
| 61.665437857 938.0 |
| 61.678568139 882.0 |
| 61.691698422 901.0 |
| 61.704828705 942.0 |
| 61.717958987 967.0 |
| 61.731089270 925.0 |
| 61.744219553 914.0 |
| 61.757349835 887.0 |
| 61.770480118 971.0 |
| 61.783610401 898.0 |
| 61.796740683 899.0 |
| 61.809870966 937.0 |
| 61.823001249 858.0 |
| 61.836131531 920.0 |
| 61.849261814 899.0 |
| 61.862392097 942.0 |
| 61.875522379 939.0 |
| 61.888652662 900.0 |
| 61.901782945 882.0 |
| 61.914913227 926.0 |
| 61.928043510 918.0 |
| 61.941173793 920.0 |
| 61.954304075 922.0 |
| 61.967434358 968.0 |
| 61.980564641 866.0 |
| 61.993694923 898.0 |
| 62.006825206 934.0 |
| 62.019955489 925.0 |
| 62.033085771 919.0 |
| 62.046216054 953.0 |
| 62.059346337 933.0 |
| 62.072476619 923.0 |
| 62.085606902 931.0 |
| 62.098737185 912.0 |
| 62.111867468 904.0 |
| 62.124997750 942.0 |
| 62.138128033 950.0 |
| 62.151258316 933.0 |
| 62.164388598 932.0 |
| 62.177518881 895.0 |
| 62.190649164 955.0 |
| 62.203779446 917.0 |
| 62.216909729 941.0 |
| 62.230040012 913.0 |
| 62.243170294 908.0 |
| 62.256300577 913.0 |
| 62.269430860 937.0 |
| 62.282561142 894.0 |
| 62.295691425 841.0 |
| 62.308821708 890.0 |
| 62.321951990 927.0 |
| 62.335082273 921.0 |
| 62.348212556 893.0 |
| 62.361342838 914.0 |
| 62.374473121 911.0 |
| 62.387603404 959.0 |
| 62.400733686 961.0 |
| 62.413863969 967.0 |
| 62.426994252 918.0 |
| 62.440124534 919.0 |
| 62.453254817 934.0 |
| 62.466385100 939.0 |
| 62.479515382 873.0 |
| 62.492645665 902.0 |
| 62.505775948 879.0 |
| 62.518906230 884.0 |
| 62.532036513 889.0 |
| 62.545166796 881.0 |
| 62.558297079 892.0 |
| 62.571427361 941.0 |
| 62.584557644 869.0 |
| 62.597687927 932.0 |
| 62.610818209 934.0 |
| 62.623948492 943.0 |
| 62.637078775 867.0 |
| 62.650209057 896.0 |
| 62.663339340 890.0 |
| 62.676469623 880.0 |
| 62.689599905 908.0 |
| 62.702730188 898.0 |
| 62.715860471 913.0 |
| 62.728990753 876.0 |
| 62.742121036 884.0 |
| 62.755251319 875.0 |
| 62.768381601 864.0 |
| 62.781511884 877.0 |
| 62.794642167 912.0 |
| 62.807772449 852.0 |
| 62.820902732 891.0 |
| 62.834033015 896.0 |
| 62.847163297 934.0 |
| 62.860293580 937.0 |
| 62.873423863 886.0 |
| 62.886554145 888.0 |
| 62.899684428 898.0 |
| 62.912814711 940.0 |
| 62.925944993 906.0 |
| 62.939075276 929.0 |
| 62.952205559 937.0 |
| 62.965335841 860.0 |
| 62.978466124 900.0 |
| 62.991596407 865.0 |
| 63.004726690 928.0 |
| 63.017856972 893.0 |
| 63.030987255 880.0 |
| 63.044117538 836.0 |
| 63.057247820 936.0 |
| 63.070378103 881.0 |
| 63.083508386 883.0 |
| 63.096638668 910.0 |
| 63.109768951 920.0 |
| 63.122899234 875.0 |
| 63.136029516 900.0 |
| 63.149159799 886.0 |
| 63.162290082 886.0 |
| 63.175420364 923.0 |
| 63.188550647 902.0 |
| 63.201680930 861.0 |
| 63.214811212 870.0 |
| 63.227941495 907.0 |
| 63.241071778 881.0 |
| 63.254202060 890.0 |
| 63.267332343 855.0 |
| 63.280462626 868.0 |
| 63.293592908 891.0 |
| 63.306723191 872.0 |
| 63.319853474 901.0 |
| 63.332983756 939.0 |
| 63.346114039 889.0 |
| 63.359244322 872.0 |
| 63.372374604 911.0 |
| 63.385504887 959.0 |
| 63.398635170 901.0 |
| 63.411765453 902.0 |
| 63.424895735 877.0 |
| 63.438026018 881.0 |
| 63.451156301 921.0 |
| 63.464286583 899.0 |
| 63.477416866 900.0 |
| 63.490547149 949.0 |
| 63.503677431 968.0 |
| 63.516807714 922.0 |
| 63.529937997 932.0 |
| 63.543068279 908.0 |
| 63.556198562 855.0 |
| 63.569328845 885.0 |
| 63.582459127 945.0 |
| 63.595589410 895.0 |
| 63.608719693 928.0 |
| 63.621849975 914.0 |
| 63.634980258 925.0 |
| 63.648110541 883.0 |
| 63.661240823 915.0 |
| 63.674371106 866.0 |
| 63.687501389 862.0 |
| 63.700631671 947.0 |
| 63.713761954 956.0 |
| 63.726892237 890.0 |
| 63.740022519 930.0 |
| 63.753152802 810.0 |
| 63.766283085 874.0 |
| 63.779413367 851.0 |
| 63.792543650 883.0 |
| 63.805673933 843.0 |
| 63.818804215 844.0 |
| 63.831934498 891.0 |
| 63.845064781 943.0 |
| 63.858195064 911.0 |
| 63.871325346 900.0 |
| 63.884455629 933.0 |
| 63.897585912 868.0 |
| 63.910716194 961.0 |
| 63.923846477 860.0 |
| 63.936976760 842.0 |
| 63.950107042 883.0 |
| 63.963237325 906.0 |
| 63.976367608 910.0 |
| 63.989497890 905.0 |
| 64.002628173 946.0 |
| 64.015758456 885.0 |
| 64.028888738 863.0 |
| 64.042019021 917.0 |
| 64.055149304 863.0 |
| 64.068279586 888.0 |
| 64.081409869 899.0 |
| 64.094540152 940.0 |
| 64.107670434 850.0 |
| 64.120800717 940.0 |
| 64.133931000 900.0 |
| 64.147061282 870.0 |
| 64.160191565 847.0 |
| 64.173321848 934.0 |
| 64.186452130 888.0 |
| 64.199582413 888.0 |
| 64.212712696 863.0 |
| 64.225842978 903.0 |
| 64.238973261 852.0 |
| 64.252103544 868.0 |
| 64.265233826 855.0 |
| 64.278364109 878.0 |
| 64.291494392 885.0 |
| 64.304624675 906.0 |
| 64.317754957 883.0 |
| 64.330885240 923.0 |
| 64.344015523 843.0 |
| 64.357145805 935.0 |
| 64.370276088 924.0 |
| 64.383406371 912.0 |
| 64.396536653 847.0 |
| 64.409666936 917.0 |
| 64.422797219 958.0 |
| 64.435927501 826.0 |
| 64.449057784 915.0 |
| 64.462188067 890.0 |
| 64.475318349 889.0 |
| 64.488448632 888.0 |
| 64.501578915 936.0 |
| 64.514709197 877.0 |
| 64.527839480 894.0 |
| 64.540969763 878.0 |
| 64.554100045 874.0 |
| 64.567230328 852.0 |
| 64.580360611 925.0 |
| 64.593490893 863.0 |
| 64.606621176 926.0 |
| 64.619751459 865.0 |
| 64.632881741 973.0 |
| 64.646012024 888.0 |
| 64.659142307 867.0 |
| 64.672272589 937.0 |
| 64.685402872 855.0 |
| 64.698533155 925.0 |
| 64.711663437 883.0 |
| 64.724793720 905.0 |
| 64.737924003 940.0 |
| 64.751054286 948.0 |
| 64.764184568 935.0 |
| 64.777314851 870.0 |
| 64.790445134 926.0 |
| 64.803575416 865.0 |
| 64.816705699 896.0 |
| 64.829835982 922.0 |
| 64.842966264 949.0 |
| 64.856096547 878.0 |
| 64.869226830 901.0 |
| 64.882357112 921.0 |
| 64.895487395 885.0 |
| 64.908617678 898.0 |
| 64.921747960 922.0 |
| 64.934878243 900.0 |
| 64.948008526 911.0 |
| 64.961138808 832.0 |
| 64.974269091 798.0 |
| 64.987399374 854.0 |
| 65.000529656 849.0 |
| 65.013659939 820.0 |
| 65.026790222 895.0 |
| 65.039920504 936.0 |
| 65.053050787 898.0 |
| 65.066181070 856.0 |
| 65.079311352 886.0 |
| 65.092441635 892.0 |
| 65.105571918 913.0 |
| 65.118702200 890.0 |
| 65.131832483 859.0 |
| 65.144962766 872.0 |
| 65.158093048 891.0 |
| 65.171223331 893.0 |
| 65.184353614 906.0 |
| 65.197483897 942.0 |
| 65.210614179 921.0 |
| 65.223744462 882.0 |
| 65.236874745 821.0 |
| 65.250005027 890.0 |
| 65.263135310 886.0 |
| 65.276265593 897.0 |
| 65.289395875 892.0 |
| 65.302526158 899.0 |
| 65.315656441 877.0 |
| 65.328786723 900.0 |
| 65.341917006 901.0 |
| 65.355047289 886.0 |
| 65.368177571 832.0 |
| 65.381307854 874.0 |
| 65.394438137 852.0 |
| 65.407568419 855.0 |
| 65.420698702 859.0 |
| 65.433828985 841.0 |
| 65.446959267 872.0 |
| 65.460089550 868.0 |
| 65.473219833 926.0 |
| 65.486350115 859.0 |
| 65.499480398 877.0 |
| 65.512610681 902.0 |
| 65.525740963 899.0 |
| 65.538871246 865.0 |
| 65.552001529 926.0 |
| 65.565131811 923.0 |
| 65.578262094 939.0 |
| 65.591392377 900.0 |
| 65.604522659 872.0 |
| 65.617652942 865.0 |
| 65.630783225 893.0 |
| 65.643913508 869.0 |
| 65.657043790 885.0 |
| 65.670174073 903.0 |
| 65.683304356 874.0 |
| 65.696434638 876.0 |
| 65.709564921 872.0 |
| 65.722695204 854.0 |
| 65.735825486 815.0 |
| 65.748955769 876.0 |
| 65.762086052 892.0 |
| 65.775216334 916.0 |
| 65.788346617 918.0 |
| 65.801476900 919.0 |
| 65.814607182 857.0 |
| 65.827737465 927.0 |
| 65.840867748 886.0 |
| 65.853998030 938.0 |
| 65.867128313 861.0 |
| 65.880258596 921.0 |
| 65.893388878 875.0 |
| 65.906519161 917.0 |
| 65.919649444 900.0 |
| 65.932779726 880.0 |
| 65.945910009 918.0 |
| 65.959040292 884.0 |
| 65.972170574 890.0 |
| 65.985300857 944.0 |
| 65.998431140 884.0 |
| 66.011561422 844.0 |
| 66.024691705 934.0 |
| 66.037821988 890.0 |
| 66.050952271 864.0 |
| 66.064082553 861.0 |
| 66.077212836 875.0 |
| 66.090343119 919.0 |
| 66.103473401 885.0 |
| 66.116603684 841.0 |
| 66.129733967 913.0 |
| 66.142864249 925.0 |
| 66.155994532 889.0 |
| 66.169124815 884.0 |
| 66.182255097 879.0 |
| 66.195385380 904.0 |
| 66.208515663 876.0 |
| 66.221645945 904.0 |
| 66.234776228 951.0 |
| 66.247906511 857.0 |
| 66.261036793 848.0 |
| 66.274167076 927.0 |
| 66.287297359 893.0 |
| 66.300427641 907.0 |
| 66.313557924 882.0 |
| 66.326688207 880.0 |
| 66.339818489 873.0 |
| 66.352948772 899.0 |
| 66.366079055 925.0 |
| 66.379209337 898.0 |
| 66.392339620 935.0 |
| 66.405469903 851.0 |
| 66.418600185 914.0 |
| 66.431730468 879.0 |
| 66.444860751 875.0 |
| 66.457991033 924.0 |
| 66.471121316 893.0 |
| 66.484251599 900.0 |
| 66.497381882 867.0 |
| 66.510512164 901.0 |
| 66.523642447 887.0 |
| 66.536772730 850.0 |
| 66.549903012 845.0 |
| 66.563033295 914.0 |
| 66.576163578 860.0 |
| 66.589293860 978.0 |
| 66.602424143 907.0 |
| 66.615554426 927.0 |
| 66.628684708 975.0 |
| 66.641814991 933.0 |
| 66.654945274 920.0 |
| 66.668075556 897.0 |
| 66.681205839 905.0 |
| 66.694336122 924.0 |
| 66.707466404 891.0 |
| 66.720596687 881.0 |
| 66.733726970 939.0 |
| 66.746857252 938.0 |
| 66.759987535 866.0 |
| 66.773117818 853.0 |
| 66.786248100 931.0 |
| 66.799378383 901.0 |
| 66.812508666 838.0 |
| 66.825638948 842.0 |
| 66.838769231 880.0 |
| 66.851899514 865.0 |
| 66.865029796 881.0 |
| 66.878160079 919.0 |
| 66.891290362 863.0 |
| 66.904420644 897.0 |
| 66.917550927 977.0 |
| 66.930681210 861.0 |
| 66.943811493 932.0 |
| 66.956941775 893.0 |
| 66.970072058 933.0 |
| 66.983202341 893.0 |
| 66.996332623 900.0 |
| 67.009462906 916.0 |
| 67.022593189 891.0 |
| 67.035723471 883.0 |
| 67.048853754 877.0 |
| 67.061984037 888.0 |
| 67.075114319 860.0 |
| 67.088244602 862.0 |
| 67.101374885 889.0 |
| 67.114505167 912.0 |
| 67.127635450 879.0 |
| 67.140765733 890.0 |
| 67.153896015 914.0 |
| 67.167026298 937.0 |
| 67.180156581 862.0 |
| 67.193286863 893.0 |
| 67.206417146 885.0 |
| 67.219547429 887.0 |
| 67.232677711 924.0 |
| 67.245807994 898.0 |
| 67.258938277 905.0 |
| 67.272068559 891.0 |
| 67.285198842 925.0 |
| 67.298329125 932.0 |
| 67.311459407 867.0 |
| 67.324589690 942.0 |
| 67.337719973 961.0 |
| 67.350850255 919.0 |
| 67.363980538 891.0 |
| 67.377110821 917.0 |
| 67.390241104 868.0 |
| 67.403371386 919.0 |
| 67.416501669 885.0 |
| 67.429631952 962.0 |
| 67.442762234 897.0 |
| 67.455892517 846.0 |
| 67.469022800 865.0 |
| 67.482153082 940.0 |
| 67.495283365 947.0 |
| 67.508413648 893.0 |
| 67.521543930 963.0 |
| 67.534674213 924.0 |
| 67.547804496 914.0 |
| 67.560934778 892.0 |
| 67.574065061 879.0 |
| 67.587195344 942.0 |
| 67.600325626 935.0 |
| 67.613455909 923.0 |
| 67.626586192 895.0 |
| 67.639716474 869.0 |
| 67.652846757 894.0 |
| 67.665977040 889.0 |
| 67.679107322 885.0 |
| 67.692237605 890.0 |
| 67.705367888 895.0 |
| 67.718498170 933.0 |
| 67.731628453 879.0 |
| 67.744758736 873.0 |
| 67.757889018 871.0 |
| 67.771019301 946.0 |
| 67.784149584 862.0 |
| 67.797279866 905.0 |
| 67.810410149 897.0 |
| 67.823540432 901.0 |
| 67.836670715 885.0 |
| 67.849800997 895.0 |
| 67.862931280 902.0 |
| 67.876061563 910.0 |
| 67.889191845 950.0 |
| 67.902322128 867.0 |
| 67.915452411 889.0 |
| 67.928582693 911.0 |
| 67.941712976 907.0 |
| 67.954843259 917.0 |
| 67.967973541 913.0 |
| 67.981103824 950.0 |
| 67.994234107 902.0 |
| 68.007364389 894.0 |
| 68.020494672 906.0 |
| 68.033624955 935.0 |
| 68.046755237 863.0 |
| 68.059885520 898.0 |
| 68.073015803 934.0 |
| 68.086146085 924.0 |
| 68.099276368 935.0 |
| 68.112406651 914.0 |
| 68.125536933 963.0 |
| 68.138667216 907.0 |
| 68.151797499 936.0 |
| 68.164927781 948.0 |
| 68.178058064 924.0 |
| 68.191188347 922.0 |
| 68.204318629 896.0 |
| 68.217448912 853.0 |
| 68.230579195 907.0 |
| 68.243709478 878.0 |
| 68.256839760 927.0 |
| 68.269970043 928.0 |
| 68.283100326 892.0 |
| 68.296230608 932.0 |
| 68.309360891 951.0 |
| 68.322491174 921.0 |
| 68.335621456 925.0 |
| 68.348751739 919.0 |
| 68.361882022 937.0 |
| 68.375012304 905.0 |
| 68.388142587 905.0 |
| 68.401272870 955.0 |
| 68.414403152 929.0 |
| 68.427533435 929.0 |
| 68.440663718 940.0 |
| 68.453794000 864.0 |
| 68.466924283 930.0 |
| 68.480054566 886.0 |
| 68.493184848 942.0 |
| 68.506315131 888.0 |
| 68.519445414 928.0 |
| 68.532575696 952.0 |
| 68.545705979 935.0 |
| 68.558836262 893.0 |
| 68.571966544 901.0 |
| 68.585096827 899.0 |
| 68.598227110 960.0 |
| 68.611357392 938.0 |
| 68.624487675 939.0 |
| 68.637617958 922.0 |
| 68.650748240 961.0 |
| 68.663878523 854.0 |
| 68.677008806 935.0 |
| 68.690139089 897.0 |
| 68.703269371 924.0 |
| 68.716399654 964.0 |
| 68.729529937 958.0 |
| 68.742660219 937.0 |
| 68.755790502 881.0 |
| 68.768920785 920.0 |
| 68.782051067 924.0 |
| 68.795181350 927.0 |
| 68.808311633 909.0 |
| 68.821441915 960.0 |
| 68.834572198 904.0 |
| 68.847702481 927.0 |
| 68.860832763 848.0 |
| 68.873963046 890.0 |
| 68.887093329 935.0 |
| 68.900223611 932.0 |
| 68.913353894 985.0 |
| 68.926484177 929.0 |
| 68.939614459 938.0 |
| 68.952744742 920.0 |
| 68.965875025 913.0 |
| 68.979005307 907.0 |
| 68.992135590 955.0 |
| 69.005265873 900.0 |
| 69.018396155 938.0 |
| 69.031526438 934.0 |
| 69.044656721 989.0 |
| 69.057787003 940.0 |
| 69.070917286 944.0 |
| 69.084047569 889.0 |
| 69.097177851 952.0 |
| 69.110308134 962.0 |
| 69.123438417 930.0 |
| 69.136568700 869.0 |
| 69.149698982 927.0 |
| 69.162829265 927.0 |
| 69.175959548 979.0 |
| 69.189089830 965.0 |
| 69.202220113 917.0 |
| 69.215350396 922.0 |
| 69.228480678 935.0 |
| 69.241610961 979.0 |
| 69.254741244 936.0 |
| 69.267871526 933.0 |
| 69.281001809 887.0 |
| 69.294132092 900.0 |
| 69.307262374 983.0 |
| 69.320392657 895.0 |
| 69.333522940 952.0 |
| 69.346653222 911.0 |
| 69.359783505 886.0 |
| 69.372913788 947.0 |
| 69.386044070 900.0 |
| 69.399174353 957.0 |
| 69.412304636 934.0 |
| 69.425434918 933.0 |
| 69.438565201 898.0 |
| 69.451695484 918.0 |
| 69.464825766 912.0 |
| 69.477956049 904.0 |
| 69.491086332 915.0 |
| 69.504216614 947.0 |
| 69.517346897 925.0 |
| 69.530477180 928.0 |
| 69.543607462 946.0 |
| 69.556737745 948.0 |
| 69.569868028 970.0 |
| 69.582998311 964.0 |
| 69.596128593 962.0 |
| 69.609258876 896.0 |
| 69.622389159 968.0 |
| 69.635519441 937.0 |
| 69.648649724 998.0 |
| 69.661780007 950.0 |
| 69.674910289 943.0 |
| 69.688040572 959.0 |
| 69.701170855 969.0 |
| 69.714301137 953.0 |
| 69.727431420 933.0 |
| 69.740561703 935.0 |
| 69.753691985 945.0 |
| 69.766822268 879.0 |
| 69.779952551 955.0 |
| 69.793082833 932.0 |
| 69.806213116 1006.0 |
| 69.819343399 1005.0 |
| 69.832473681 987.0 |
| 69.845603964 938.0 |
| 69.858734247 921.0 |
| 69.871864529 942.0 |
| 69.884994812 853.0 |
| 69.898125095 960.0 |
| 69.911255377 877.0 |
| 69.924385660 915.0 |
| 69.937515943 977.0 |
| 69.950646225 967.0 |
| 69.963776508 942.0 |
| 69.976906791 955.0 |
| 69.990037073 913.0 |
| 70.003167356 904.0 |
| 70.016297639 954.0 |
| 70.029427922 904.0 |
| 70.042558204 923.0 |
| 70.055688487 935.0 |
| 70.068818770 948.0 |
| 70.081949052 930.0 |
| 70.095079335 903.0 |
| 70.108209618 935.0 |
| 70.121339900 975.0 |
| 70.134470183 952.0 |
| 70.147600466 951.0 |
| 70.160730748 942.0 |
| 70.173861031 953.0 |
| 70.186991314 916.0 |
| 70.200121596 898.0 |
| 70.213251879 928.0 |
| 70.226382162 881.0 |
| 70.239512444 939.0 |
| 70.252642727 894.0 |
| 70.265773010 949.0 |
| 70.278903292 937.0 |
| 70.292033575 954.0 |
| 70.305163858 888.0 |
| 70.318294140 900.0 |
| 70.331424423 891.0 |
| 70.344554706 939.0 |
| 70.357684988 930.0 |
| 70.370815271 941.0 |
| 70.383945554 892.0 |
| 70.397075836 953.0 |
| 70.410206119 979.0 |
| 70.423336402 933.0 |
| 70.436466684 921.0 |
| 70.449596967 908.0 |
| 70.462727250 928.0 |
| 70.475857533 970.0 |
| 70.488987815 914.0 |
| 70.502118098 947.0 |
| 70.515248381 944.0 |
| 70.528378663 896.0 |
| 70.541508946 932.0 |
| 70.554639229 955.0 |
| 70.567769511 905.0 |
| 70.580899794 932.0 |
| 70.594030077 934.0 |
| 70.607160359 899.0 |
| 70.620290642 931.0 |
| 70.633420925 941.0 |
| 70.646551207 948.0 |
| 70.659681490 933.0 |
| 70.672811773 930.0 |
| 70.685942055 926.0 |
| 70.699072338 845.0 |
| 70.712202621 900.0 |
| 70.725332903 948.0 |
| 70.738463186 911.0 |
| 70.751593469 962.0 |
| 70.764723751 921.0 |
| 70.777854034 900.0 |
| 70.790984317 933.0 |
| 70.804114599 922.0 |
| 70.817244882 939.0 |
| 70.830375165 911.0 |
| 70.843505447 926.0 |
| 70.856635730 937.0 |
| 70.869766013 968.0 |
| 70.882896296 907.0 |
| 70.896026578 842.0 |
| 70.909156861 901.0 |
| 70.922287144 932.0 |
| 70.935417426 984.0 |
| 70.948547709 880.0 |
| 70.961677992 942.0 |
| 70.974808274 889.0 |
| 70.987938557 949.0 |
| 71.001068840 937.0 |
| 71.014199122 888.0 |
| 71.027329405 936.0 |
| 71.040459688 937.0 |
| 71.053589970 863.0 |
| 71.066720253 888.0 |
| 71.079850536 909.0 |
| 71.092980818 877.0 |
| 71.106111101 896.0 |
| 71.119241384 906.0 |
| 71.132371666 904.0 |
| 71.145501949 922.0 |
| 71.158632232 895.0 |
| 71.171762514 848.0 |
| 71.184892797 946.0 |
| 71.198023080 914.0 |
| 71.211153362 868.0 |
| 71.224283645 922.0 |
| 71.237413928 909.0 |
| 71.250544210 943.0 |
| 71.263674493 919.0 |
| 71.276804776 863.0 |
| 71.289935058 880.0 |
| 71.303065341 886.0 |
| 71.316195624 903.0 |
| 71.329325907 901.0 |
| 71.342456189 912.0 |
| 71.355586472 893.0 |
| 71.368716755 913.0 |
| 71.381847037 863.0 |
| 71.394977320 909.0 |
| 71.408107603 907.0 |
| 71.421237885 885.0 |
| 71.434368168 958.0 |
| 71.447498451 920.0 |
| 71.460628733 869.0 |
| 71.473759016 891.0 |
| 71.486889299 920.0 |
| 71.500019581 932.0 |
| 71.513149864 893.0 |
| 71.526280147 912.0 |
| 71.539410429 905.0 |
| 71.552540712 903.0 |
| 71.565670995 876.0 |
| 71.578801277 904.0 |
| 71.591931560 861.0 |
| 71.605061843 894.0 |
| 71.618192125 904.0 |
| 71.631322408 886.0 |
| 71.644452691 939.0 |
| 71.657582973 928.0 |
| 71.670713256 888.0 |
| 71.683843539 903.0 |
| 71.696973821 916.0 |
| 71.710104104 886.0 |
| 71.723234387 842.0 |
| 71.736364669 943.0 |
| 71.749494952 861.0 |
| 71.762625235 825.0 |
| 71.775755518 906.0 |
| 71.788885800 869.0 |
| 71.802016083 841.0 |
| 71.815146366 812.0 |
| 71.828276648 884.0 |
| 71.841406931 891.0 |
| 71.854537214 922.0 |
| 71.867667496 868.0 |
| 71.880797779 862.0 |
| 71.893928062 830.0 |
| 71.907058344 897.0 |
| 71.920188627 882.0 |
| 71.933318910 840.0 |
| 71.946449192 915.0 |
| 71.959579475 880.0 |
| 71.972709758 924.0 |
| 71.985840040 894.0 |
| 71.998970323 886.0 |
| 72.012100606 867.0 |
| 72.025230888 869.0 |
| 72.038361171 848.0 |
| 72.051491454 876.0 |
| 72.064621736 923.0 |
| 72.077752019 858.0 |
| 72.090882302 889.0 |
| 72.104012584 859.0 |
| 72.117142867 912.0 |
| 72.130273150 854.0 |
| 72.143403432 829.0 |
| 72.156533715 881.0 |
| 72.169663998 849.0 |
| 72.182794280 845.0 |
| 72.195924563 902.0 |
| 72.209054846 806.0 |
| 72.222185129 854.0 |
| 72.235315411 856.0 |
| 72.248445694 896.0 |
| 72.261575977 903.0 |
| 72.274706259 927.0 |
| 72.287836542 891.0 |
| 72.300966825 848.0 |
| 72.314097107 852.0 |
| 72.327227390 907.0 |
| 72.340357673 789.0 |
| 72.353487955 908.0 |
| 72.366618238 772.0 |
| 72.379748521 864.0 |
| 72.392878803 837.0 |
| 72.406009086 876.0 |
| 72.419139369 874.0 |
| 72.432269651 833.0 |
| 72.445399934 848.0 |
| 72.458530217 873.0 |
| 72.471660499 845.0 |
| 72.484790782 868.0 |
| 72.497921065 840.0 |
| 72.511051347 816.0 |
| 72.524181630 863.0 |
| 72.537311913 907.0 |
| 72.550442195 848.0 |
| 72.563572478 842.0 |
| 72.576702761 838.0 |
| 72.589833043 908.0 |
| 72.602963326 824.0 |
| 72.616093609 847.0 |
| 72.629223891 829.0 |
| 72.642354174 864.0 |
| 72.655484457 827.0 |
| 72.668614740 825.0 |
| 72.681745022 800.0 |
| 72.694875305 806.0 |
| 72.708005588 851.0 |
| 72.721135870 858.0 |
| 72.734266153 866.0 |
| 72.747396436 852.0 |
| 72.760526718 793.0 |
| 72.773657001 851.0 |
| 72.786787284 854.0 |
| 72.799917566 818.0 |
| 72.813047849 832.0 |
| 72.826178132 901.0 |
| 72.839308414 858.0 |
| 72.852438697 906.0 |
| 72.865568980 853.0 |
| 72.878699262 854.0 |
| 72.891829545 852.0 |
| 72.904959828 849.0 |
| 72.918090110 829.0 |
| 72.931220393 891.0 |
| 72.944350676 818.0 |
| 72.957480958 852.0 |
| 72.970611241 833.0 |
| 72.983741524 882.0 |
| 72.996871806 808.0 |
| 73.010002089 846.0 |
| 73.023132372 804.0 |
| 73.036262654 883.0 |
| 73.049392937 860.0 |
| 73.062523220 876.0 |
| 73.075653503 825.0 |
| 73.088783785 823.0 |
| 73.101914068 832.0 |
| 73.115044351 834.0 |
| 73.128174633 830.0 |
| 73.141304916 805.0 |
| 73.154435199 845.0 |
| 73.167565481 786.0 |
| 73.180695764 822.0 |
| 73.193826047 831.0 |
| 73.206956329 833.0 |
| 73.220086612 846.0 |
| 73.233216895 829.0 |
| 73.246347177 843.0 |
| 73.259477460 834.0 |
| 73.272607743 846.0 |
| 73.285738025 824.0 |
| 73.298868308 857.0 |
| 73.311998591 876.0 |
| 73.325128873 870.0 |
| 73.338259156 859.0 |
| 73.351389439 794.0 |
| 73.364519721 815.0 |
| 73.377650004 823.0 |
| 73.390780287 815.0 |
| 73.403910569 857.0 |
| 73.417040852 865.0 |
| 73.430171135 837.0 |
| 73.443301417 834.0 |
| 73.456431700 842.0 |
| 73.469561983 804.0 |
| 73.482692265 830.0 |
| 73.495822548 859.0 |
| 73.508952831 848.0 |
| 73.522083114 816.0 |
| 73.535213396 862.0 |
| 73.548343679 879.0 |
| 73.561473962 816.0 |
| 73.574604244 841.0 |
| 73.587734527 804.0 |
| 73.600864810 841.0 |
| 73.613995092 854.0 |
| 73.627125375 855.0 |
| 73.640255658 838.0 |
| 73.653385940 838.0 |
| 73.666516223 845.0 |
| 73.679646506 780.0 |
| 73.692776788 839.0 |
| 73.705907071 825.0 |
| 73.719037354 822.0 |
| 73.732167636 847.0 |
| 73.745297919 872.0 |
| 73.758428202 845.0 |
| 73.771558484 835.0 |
| 73.784688767 880.0 |
| 73.797819050 859.0 |
| 73.810949332 853.0 |
| 73.824079615 799.0 |
| 73.837209898 844.0 |
| 73.850340180 860.0 |
| 73.863470463 797.0 |
| 73.876600746 809.0 |
| 73.889731028 839.0 |
| 73.902861311 813.0 |
| 73.915991594 801.0 |
| 73.929121876 834.0 |
| 73.942252159 862.0 |
| 73.955382442 833.0 |
| 73.968512725 836.0 |
| 73.981643007 835.0 |
| 73.994773290 836.0 |
| 74.007903573 775.0 |
| 74.021033855 826.0 |
| 74.034164138 827.0 |
| 74.047294421 822.0 |
| 74.060424703 886.0 |
| 74.073554986 816.0 |
| 74.086685269 819.0 |
| 74.099815551 832.0 |
| 74.112945834 787.0 |
| 74.126076117 840.0 |
| 74.139206399 834.0 |
| 74.152336682 810.0 |
| 74.165466965 793.0 |
| 74.178597247 817.0 |
| 74.191727530 781.0 |
| 74.204857813 832.0 |
| 74.217988095 818.0 |
| 74.231118378 822.0 |
| 74.244248661 804.0 |
| 74.257378943 808.0 |
| 74.270509226 845.0 |
| 74.283639509 818.0 |
| 74.296769791 846.0 |
| 74.309900074 768.0 |
| 74.323030357 782.0 |
| 74.336160639 863.0 |
| 74.349290922 794.0 |
| 74.362421205 807.0 |
| 74.375551487 844.0 |
| 74.388681770 851.0 |
| 74.401812053 796.0 |
| 74.414942336 813.0 |
| 74.428072618 861.0 |
| 74.441202901 832.0 |
| 74.454333184 817.0 |
| 74.467463466 796.0 |
| 74.480593749 835.0 |
| 74.493724032 773.0 |
| 74.506854314 804.0 |
| 74.519984597 839.0 |
| 74.533114880 798.0 |
| 74.546245162 866.0 |
| 74.559375445 787.0 |
| 74.572505728 795.0 |
| 74.585636010 833.0 |
| 74.598766293 751.0 |
| 74.611896576 848.0 |
| 74.625026858 818.0 |
| 74.638157141 790.0 |
| 74.651287424 824.0 |
| 74.664417706 778.0 |
| 74.677547989 835.0 |
| 74.690678272 797.0 |
| 74.703808554 802.0 |
| 74.716938837 847.0 |
| 74.730069120 816.0 |
| 74.743199402 788.0 |
| 74.756329685 772.0 |
| 74.769459968 811.0 |
| 74.782590250 781.0 |
| 74.795720533 824.0 |
| 74.808850816 842.0 |
| 74.821981098 828.0 |
| 74.835111381 824.0 |
| 74.848241664 802.0 |
| 74.861371947 804.0 |
| 74.874502229 799.0 |
| 74.887632512 769.0 |
| 74.900762795 820.0 |
| 74.913893077 863.0 |
| 74.927023360 816.0 |
| 74.940153643 820.0 |
| 74.953283925 785.0 |
| 74.966414208 822.0 |
| 74.979544491 827.0 |
| 74.992674773 819.0 |
| 75.005805056 805.0 |
| 75.018935339 764.0 |
| 75.032065621 777.0 |
| 75.045195904 848.0 |
| 75.058326187 804.0 |
| 75.071456469 825.0 |
| 75.084586752 826.0 |
| 75.097717035 817.0 |
| 75.110847317 832.0 |
| 75.123977600 791.0 |
| 75.137107883 874.0 |
| 75.150238165 774.0 |
| 75.163368448 826.0 |
| 75.176498731 778.0 |
| 75.189629013 790.0 |
| 75.202759296 787.0 |
| 75.215889579 802.0 |
| 75.229019861 815.0 |
| 75.242150144 816.0 |
| 75.255280427 838.0 |
| 75.268410709 814.0 |
| 75.281540992 799.0 |
| 75.294671275 828.0 |
| 75.307801558 793.0 |
| 75.320931840 763.0 |
| 75.334062123 815.0 |
| 75.347192406 828.0 |
| 75.360322688 814.0 |
| 75.373452971 794.0 |
| 75.386583254 771.0 |
| 75.399713536 810.0 |
| 75.412843819 803.0 |
| 75.425974102 777.0 |
| 75.439104384 855.0 |
| 75.452234667 808.0 |
| 75.465364950 804.0 |
| 75.478495232 803.0 |
| 75.491625515 831.0 |
| 75.504755798 814.0 |
| 75.517886080 837.0 |
| 75.531016363 774.0 |
| 75.544146646 775.0 |
| 75.557276928 810.0 |
| 75.570407211 818.0 |
| 75.583537494 805.0 |
| 75.596667776 748.0 |
| 75.609798059 804.0 |
| 75.622928342 810.0 |
| 75.636058624 821.0 |
| 75.649188907 791.0 |
| 75.662319190 776.0 |
| 75.675449472 856.0 |
| 75.688579755 775.0 |
| 75.701710038 804.0 |
| 75.714840321 855.0 |
| 75.727970603 777.0 |
| 75.741100886 796.0 |
| 75.754231169 735.0 |
| 75.767361451 821.0 |
| 75.780491734 799.0 |
| 75.793622017 771.0 |
| 75.806752299 770.0 |
| 75.819882582 808.0 |
| 75.833012865 782.0 |
| 75.846143147 754.0 |
| 75.859273430 835.0 |
| 75.872403713 784.0 |
| 75.885533995 812.0 |
| 75.898664278 811.0 |
| 75.911794561 762.0 |
| 75.924924843 825.0 |
| 75.938055126 789.0 |
| 75.951185409 869.0 |
| 75.964315691 924.0 |
| 75.977445974 823.0 |
| 75.990576257 821.0 |
| 76.003706539 777.0 |
| 76.016836822 833.0 |
| 76.029967105 766.0 |
| 76.043097387 861.0 |
| 76.056227670 805.0 |
| 76.069357953 847.0 |
| 76.082488235 832.0 |
| 76.095618518 814.0 |
| 76.108748801 782.0 |
| 76.121879083 822.0 |
| 76.135009366 807.0 |
| 76.148139649 782.0 |
| 76.161269932 809.0 |
| 76.174400214 757.0 |
| 76.187530497 803.0 |
| 76.200660780 773.0 |
| 76.213791062 784.0 |
| 76.226921345 802.0 |
| 76.240051628 775.0 |
| 76.253181910 804.0 |
| 76.266312193 815.0 |
| 76.279442476 788.0 |
| 76.292572758 852.0 |
| 76.305703041 788.0 |
| 76.318833324 773.0 |
| 76.331963606 837.0 |
| 76.345093889 820.0 |
| 76.358224172 783.0 |
| 76.371354454 826.0 |
| 76.384484737 775.0 |
| 76.397615020 746.0 |
| 76.410745302 764.0 |
| 76.423875585 812.0 |
| 76.437005868 827.0 |
| 76.450136150 794.0 |
| 76.463266433 804.0 |
| 76.476396716 823.0 |
| 76.489526998 786.0 |
| 76.502657281 815.0 |
| 76.515787564 791.0 |
| 76.528917846 805.0 |
| 76.542048129 797.0 |
| 76.555178412 840.0 |
| 76.568308694 790.0 |
| 76.581438977 809.0 |
| 76.594569260 776.0 |
| 76.607699543 877.0 |
| 76.620829825 775.0 |
| 76.633960108 823.0 |
| 76.647090391 769.0 |
| 76.660220673 808.0 |
| 76.673350956 785.0 |
| 76.686481239 789.0 |
| 76.699611521 740.0 |
| 76.712741804 843.0 |
| 76.725872087 865.0 |
| 76.739002369 849.0 |
| 76.752132652 834.0 |
| 76.765262935 795.0 |
| 76.778393217 784.0 |
| 76.791523500 790.0 |
| 76.804653783 795.0 |
| 76.817784065 825.0 |
| 76.830914348 818.0 |
| 76.844044631 774.0 |
| 76.857174913 797.0 |
| 76.870305196 844.0 |
| 76.883435479 856.0 |
| 76.896565761 787.0 |
| 76.909696044 799.0 |
| 76.922826327 822.0 |
| 76.935956609 806.0 |
| 76.949086892 849.0 |
| 76.962217175 809.0 |
| 76.975347457 761.0 |
| 76.988477740 730.0 |
| 77.001608023 844.0 |
| 77.014738305 826.0 |
| 77.027868588 819.0 |
| 77.040998871 787.0 |
| 77.054129154 805.0 |
| 77.067259436 778.0 |
| 77.080389719 776.0 |
| 77.093520002 790.0 |
| 77.106650284 797.0 |
| 77.119780567 822.0 |
| 77.132910850 830.0 |
| 77.146041132 803.0 |
| 77.159171415 814.0 |
| 77.172301698 756.0 |
| 77.185431980 794.0 |
| 77.198562263 818.0 |
| 77.211692546 853.0 |
| 77.224822828 758.0 |
| 77.237953111 793.0 |
| 77.251083394 810.0 |
| 77.264213676 838.0 |
| 77.277343959 879.0 |
| 77.290474242 776.0 |
| 77.303604524 810.0 |
| 77.316734807 832.0 |
| 77.329865090 769.0 |
| 77.342995372 828.0 |
| 77.356125655 822.0 |
| 77.369255938 868.0 |
| 77.382386220 829.0 |
| 77.395516503 840.0 |
| 77.408646786 832.0 |
| 77.421777068 797.0 |
| 77.434907351 801.0 |
| 77.448037634 797.0 |
| 77.461167916 824.0 |
| 77.474298199 800.0 |
| 77.487428482 808.0 |
| 77.500558765 839.0 |
| 77.513689047 795.0 |
| 77.526819330 824.0 |
| 77.539949613 783.0 |
| 77.553079895 862.0 |
| 77.566210178 843.0 |
| 77.579340461 813.0 |
| 77.592470743 807.0 |
| 77.605601026 832.0 |
| 77.618731309 791.0 |
| 77.631861591 811.0 |
| 77.644991874 861.0 |
| 77.658122157 791.0 |
| 77.671252439 862.0 |
| 77.684382722 798.0 |
| 77.697513005 802.0 |
| 77.710643287 835.0 |
| 77.723773570 821.0 |
| 77.736903853 888.0 |
| 77.750034135 849.0 |
| 77.763164418 921.0 |
| 77.776294701 852.0 |
| 77.789424983 830.0 |
| 77.802555266 783.0 |
| 77.815685549 808.0 |
| 77.828815831 796.0 |
| 77.841946114 818.0 |
| 77.855076397 803.0 |
| 77.868206679 822.0 |
| 77.881336962 830.0 |
| 77.894467245 839.0 |
| 77.907597528 848.0 |
| 77.920727810 809.0 |
| 77.933858093 816.0 |
| 77.946988376 816.0 |
| 77.960118658 811.0 |
| 77.973248941 846.0 |
| 77.986379224 855.0 |
| 77.999509506 800.0 |
| 78.012639789 825.0 |
| 78.025770072 834.0 |
| 78.038900354 821.0 |
| 78.052030637 862.0 |
| 78.065160920 870.0 |
| 78.078291202 871.0 |
| 78.091421485 821.0 |
| 78.104551768 810.0 |
| 78.117682050 851.0 |
| 78.130812333 847.0 |
| 78.143942616 828.0 |
| 78.157072898 817.0 |
| 78.170203181 839.0 |
| 78.183333464 893.0 |
| 78.196463746 806.0 |
| 78.209594029 844.0 |
| 78.222724312 844.0 |
| 78.235854594 862.0 |
| 78.248984877 843.0 |
| 78.262115160 834.0 |
| 78.275245442 845.0 |
| 78.288375725 840.0 |
| 78.301506008 765.0 |
| 78.314636290 794.0 |
| 78.327766573 856.0 |
| 78.340896856 803.0 |
| 78.354027139 807.0 |
| 78.367157421 865.0 |
| 78.380287704 827.0 |
| 78.393417987 792.0 |
| 78.406548269 795.0 |
| 78.419678552 878.0 |
| 78.432808835 810.0 |
| 78.445939117 811.0 |
| 78.459069400 817.0 |
| 78.472199683 815.0 |
| 78.485329965 788.0 |
| 78.498460248 791.0 |
| 78.511590531 811.0 |
| 78.524720813 884.0 |
| 78.537851096 841.0 |
| 78.550981379 849.0 |
| 78.564111661 778.0 |
| 78.577241944 823.0 |
| 78.590372227 808.0 |
| 78.603502509 862.0 |
| 78.616632792 800.0 |
| 78.629763075 831.0 |
| 78.642893357 838.0 |
| 78.656023640 826.0 |
| 78.669153923 811.0 |
| 78.682284205 852.0 |
| 78.695414488 860.0 |
| 78.708544771 798.0 |
| 78.721675053 858.0 |
| 78.734805336 779.0 |
| 78.747935619 874.0 |
| 78.761065901 808.0 |
| 78.774196184 791.0 |
| 78.787326467 871.0 |
| 78.800456750 855.0 |
| 78.813587032 833.0 |
| 78.826717315 850.0 |
| 78.839847598 820.0 |
| 78.852977880 859.0 |
| 78.866108163 822.0 |
| 78.879238446 853.0 |
| 78.892368728 784.0 |
| 78.905499011 819.0 |
| 78.918629294 818.0 |
| 78.931759576 838.0 |
| 78.944889859 811.0 |
| 78.958020142 831.0 |
| 78.971150424 874.0 |
| 78.984280707 817.0 |
| 78.997410990 823.0 |
| 79.010541272 857.0 |
| 79.023671555 825.0 |
| 79.036801838 865.0 |
| 79.049932120 862.0 |
| 79.063062403 824.0 |
| 79.076192686 881.0 |
| 79.089322968 841.0 |
| 79.102453251 840.0 |
| 79.115583534 797.0 |
| 79.128713816 851.0 |
| 79.141844099 825.0 |
| 79.154974382 869.0 |
| 79.168104664 808.0 |
| 79.181234947 863.0 |
| 79.194365230 824.0 |
| 79.207495512 792.0 |
| 79.220625795 823.0 |
| 79.233756078 843.0 |
| 79.246886361 857.0 |
| 79.260016643 749.0 |
| 79.273146926 771.0 |
| 79.286277209 771.0 |
| 79.299407491 801.0 |
| 79.312537774 820.0 |
| 79.325668057 841.0 |
| 79.338798339 872.0 |
| 79.351928622 845.0 |
| 79.365058905 895.0 |
| 79.378189187 835.0 |
| 79.391319470 760.0 |
| 79.404449753 840.0 |
| 79.417580035 885.0 |
| 79.430710318 825.0 |
| 79.443840601 847.0 |
| 79.456970883 833.0 |
| 79.470101166 807.0 |
| 79.483231449 801.0 |
| 79.496361731 846.0 |
| 79.509492014 768.0 |
| 79.522622297 796.0 |
| 79.535752579 762.0 |
| 79.548882862 785.0 |
| 79.562013145 774.0 |
| 79.575143427 840.0 |
| 79.588273710 764.0 |
| 79.601403993 808.0 |
| 79.614534275 799.0 |
| 79.627664558 856.0 |
| 79.640794841 828.0 |
| 79.653925123 797.0 |
| 79.667055406 826.0 |
| 79.680185689 858.0 |
| 79.693315972 802.0 |
| 79.706446254 844.0 |
| 79.719576537 787.0 |
| 79.732706820 809.0 |
| 79.745837102 819.0 |
| 79.758967385 851.0 |
| 79.772097668 814.0 |
| 79.785227950 827.0 |
| 79.798358233 791.0 |
| 79.811488516 854.0 |
| 79.824618798 914.0 |
| 79.837749081 812.0 |
| 79.850879364 823.0 |
| 79.864009646 864.0 |
| 79.877139929 935.0 |
| 79.890270212 858.0 |
| 79.903400494 793.0 |
| 79.916530777 847.0 |
| 79.929661060 791.0 |
| 79.942791342 927.0 |
| 79.955921625 793.0 |
| 79.969051908 857.0 |
| 79.982182190 800.0 |
| 79.995312473 792.0 |
| 80.008442756 891.0 |
| 80.021573038 835.0 |
| 80.034703321 821.0 |
| 80.047833604 845.0 |
| 80.060963886 852.0 |
| 80.074094169 823.0 |
| 80.087224452 866.0 |
| 80.100354734 793.0 |
| 80.113485017 846.0 |
| 80.126615300 833.0 |
| 80.139745583 851.0 |
| 80.152875865 842.0 |
| 80.166006148 798.0 |
| 80.179136431 817.0 |
| 80.192266713 832.0 |
| 80.205396996 833.0 |
| 80.218527279 818.0 |
| 80.231657561 817.0 |
| 80.244787844 847.0 |
| 80.257918127 845.0 |
| 80.271048409 799.0 |
| 80.284178692 773.0 |
| 80.297308975 836.0 |
| 80.310439257 815.0 |
| 80.323569540 837.0 |
| 80.336699823 817.0 |
| 80.349830105 862.0 |
| 80.362960388 829.0 |
| 80.376090671 807.0 |
| 80.389220953 791.0 |
| 80.402351236 781.0 |
| 80.415481519 769.0 |
| 80.428611801 816.0 |
| 80.441742084 841.0 |
| 80.454872367 801.0 |
| 80.468002649 785.0 |
| 80.481132932 824.0 |
| 80.494263215 850.0 |
| 80.507393497 801.0 |
| 80.520523780 827.0 |
| 80.533654063 830.0 |
| 80.546784346 871.0 |
| 80.559914628 795.0 |
| 80.573044911 810.0 |
| 80.586175194 833.0 |
| 80.599305476 820.0 |
| 80.612435759 834.0 |
| 80.625566042 853.0 |
| 80.638696324 869.0 |
| 80.651826607 847.0 |
| 80.664956890 849.0 |
| 80.678087172 838.0 |
| 80.691217455 865.0 |
| 80.704347738 839.0 |
| 80.717478020 817.0 |
| 80.730608303 775.0 |
| 80.743738586 840.0 |
| 80.756868868 848.0 |
| 80.769999151 822.0 |
| 80.783129434 825.0 |
| 80.796259716 841.0 |
| 80.809389999 842.0 |
| 80.822520282 828.0 |
| 80.835650564 828.0 |
| 80.848780847 793.0 |
| 80.861911130 833.0 |
| 80.875041412 861.0 |
| 80.888171695 806.0 |
| 80.901301978 843.0 |
| 80.914432260 848.0 |
| 80.927562543 832.0 |
| 80.940692826 854.0 |
| 80.953823108 836.0 |
| 80.966953391 849.0 |
| 80.980083674 768.0 |
| 80.993213957 807.0 |
| 81.006344239 852.0 |
| 81.019474522 849.0 |
| 81.032604805 823.0 |
| 81.045735087 873.0 |
| 81.058865370 813.0 |
| 81.071995653 720.0 |
| 81.085125935 760.0 |
| 81.098256218 829.0 |
| 81.111386501 802.0 |
| 81.124516783 807.0 |
| 81.137647066 785.0 |
| 81.150777349 836.0 |
| 81.163907631 877.0 |
| 81.177037914 866.0 |
| 81.190168197 860.0 |
| 81.203298479 875.0 |
| 81.216428762 845.0 |
| 81.229559045 794.0 |
| 81.242689327 857.0 |
| 81.255819610 843.0 |
| 81.268949893 839.0 |
| 81.282080175 812.0 |
| 81.295210458 865.0 |
| 81.308340741 801.0 |
| 81.321471023 840.0 |
| 81.334601306 862.0 |
| 81.347731589 822.0 |
| 81.360861871 879.0 |
| 81.373992154 915.0 |
| 81.387122437 906.0 |
| 81.400252719 842.0 |
| 81.413383002 817.0 |
| 81.426513285 832.0 |
| 81.439643568 853.0 |
| 81.452773850 901.0 |
| 81.465904133 834.0 |
| 81.479034416 760.0 |
| 81.492164698 872.0 |
| 81.505294981 838.0 |
| 81.518425264 900.0 |
| 81.531555546 813.0 |
| 81.544685829 825.0 |
| 81.557816112 833.0 |
| 81.570946394 811.0 |
| 81.584076677 830.0 |
| 81.597206960 836.0 |
| 81.610337242 867.0 |
| 81.623467525 829.0 |
| 81.636597808 789.0 |
| 81.649728090 823.0 |
| 81.662858373 874.0 |
| 81.675988656 844.0 |
| 81.689118938 885.0 |
| 81.702249221 801.0 |
| 81.715379504 860.0 |
| 81.728509786 912.0 |
| 81.741640069 814.0 |
| 81.754770352 863.0 |
| 81.767900634 853.0 |
| 81.781030917 832.0 |
| 81.794161200 900.0 |
| 81.807291482 859.0 |
| 81.820421765 809.0 |
| 81.833552048 857.0 |
| 81.846682330 831.0 |
| 81.859812613 846.0 |
| 81.872942896 841.0 |
| 81.886073179 874.0 |
| 81.899203461 887.0 |
| 81.912333744 876.0 |
| 81.925464027 806.0 |
| 81.938594309 910.0 |
| 81.951724592 822.0 |
| 81.964854875 827.0 |
| 81.977985157 827.0 |
| 81.991115440 831.0 |
| 82.004245723 891.0 |
| 82.017376005 814.0 |
| 82.030506288 860.0 |
| 82.043636571 855.0 |
| 82.056766853 813.0 |
| 82.069897136 863.0 |
| 82.083027419 864.0 |
| 82.096157701 863.0 |
| 82.109287984 790.0 |
| 82.122418267 900.0 |
| 82.135548549 844.0 |
| 82.148678832 854.0 |
| 82.161809115 850.0 |
| 82.174939397 840.0 |
| 82.188069680 857.0 |
| 82.201199963 890.0 |
| 82.214330245 821.0 |
| 82.227460528 822.0 |
| 82.240590811 815.0 |
| 82.253721093 855.0 |
| 82.266851376 804.0 |
| 82.279981659 826.0 |
| 82.293111941 792.0 |
| 82.306242224 843.0 |
| 82.319372507 886.0 |
| 82.332502790 800.0 |
| 82.345633072 846.0 |
| 82.358763355 844.0 |
| 82.371893638 837.0 |
| 82.385023920 833.0 |
| 82.398154203 880.0 |
| 82.411284486 882.0 |
| 82.424414768 842.0 |
| 82.437545051 872.0 |
| 82.450675334 882.0 |
| 82.463805616 840.0 |
| 82.476935899 827.0 |
| 82.490066182 871.0 |
| 82.503196464 841.0 |
| 82.516326747 896.0 |
| 82.529457030 847.0 |
| 82.542587312 842.0 |
| 82.555717595 849.0 |
| 82.568847878 867.0 |
| 82.581978160 805.0 |
| 82.595108443 893.0 |
| 82.608238726 860.0 |
| 82.621369008 829.0 |
| 82.634499291 855.0 |
| 82.647629574 888.0 |
| 82.660759856 817.0 |
| 82.673890139 831.0 |
| 82.687020422 844.0 |
| 82.700150704 834.0 |
| 82.713280987 842.0 |
| 82.726411270 875.0 |
| 82.739541553 849.0 |
| 82.752671835 832.0 |
| 82.765802118 793.0 |
| 82.778932401 848.0 |
| 82.792062683 830.0 |
| 82.805192966 852.0 |
| 82.818323249 859.0 |
| 82.831453531 891.0 |
| 82.844583814 865.0 |
| 82.857714097 858.0 |
| 82.870844379 857.0 |
| 82.883974662 893.0 |
| 82.897104945 901.0 |
| 82.910235227 822.0 |
| 82.923365510 859.0 |
| 82.936495793 832.0 |
| 82.949626075 831.0 |
| 82.962756358 842.0 |
| 82.975886641 841.0 |
| 82.989016923 871.0 |
| 83.002147206 861.0 |
| 83.015277489 891.0 |
| 83.028407771 832.0 |
| 83.041538054 910.0 |
| 83.054668337 863.0 |
| 83.067798619 842.0 |
| 83.080928902 869.0 |
| 83.094059185 801.0 |
| 83.107189467 861.0 |
| 83.120319750 877.0 |
| 83.133450033 906.0 |
| 83.146580315 856.0 |
| 83.159710598 819.0 |
| 83.172840881 866.0 |
| 83.185971164 910.0 |
| 83.199101446 871.0 |
| 83.212231729 847.0 |
| 83.225362012 864.0 |
| 83.238492294 858.0 |
| 83.251622577 903.0 |
| 83.264752860 860.0 |
| 83.277883142 824.0 |
| 83.291013425 855.0 |
| 83.304143708 876.0 |
| 83.317273990 859.0 |
| 83.330404273 841.0 |
| 83.343534556 827.0 |
| 83.356664838 868.0 |
| 83.369795121 823.0 |
| 83.382925404 885.0 |
| 83.396055686 899.0 |
| 83.409185969 867.0 |
| 83.422316252 823.0 |
| 83.435446534 839.0 |
| 83.448576817 849.0 |
| 83.461707100 819.0 |
| 83.474837382 843.0 |
| 83.487967665 834.0 |
| 83.501097948 828.0 |
| 83.514228230 819.0 |
| 83.527358513 850.0 |
| 83.540488796 859.0 |
| 83.553619078 847.0 |
| 83.566749361 828.0 |
| 83.579879644 838.0 |
| 83.593009926 846.0 |
| 83.606140209 848.0 |
| 83.619270492 898.0 |
| 83.632400775 901.0 |
| 83.645531057 869.0 |
| 83.658661340 854.0 |
| 83.671791623 848.0 |
| 83.684921905 894.0 |
| 83.698052188 826.0 |
| 83.711182471 844.0 |
| 83.724312753 860.0 |
| 83.737443036 830.0 |
| 83.750573319 830.0 |
| 83.763703601 848.0 |
| 83.776833884 836.0 |
| 83.789964167 850.0 |
| 83.803094449 922.0 |
| 83.816224732 908.0 |
| 83.829355015 902.0 |
| 83.842485297 911.0 |
| 83.855615580 826.0 |
| 83.868745863 862.0 |
| 83.881876145 834.0 |
| 83.895006428 850.0 |
| 83.908136711 864.0 |
| 83.921266993 844.0 |
| 83.934397276 832.0 |
| 83.947527559 893.0 |
| 83.960657841 832.0 |
| 83.973788124 907.0 |
| 83.986918407 925.0 |
| 84.000048689 895.0 |
| 84.013178972 834.0 |
| 84.026309255 879.0 |
| 84.039439537 855.0 |
| 84.052569820 843.0 |
| 84.065700103 825.0 |
| 84.078830386 841.0 |
| 84.091960668 794.0 |
| 84.105090951 871.0 |
| 84.118221234 796.0 |
| 84.131351516 843.0 |
| 84.144481799 812.0 |
| 84.157612082 845.0 |
| 84.170742364 893.0 |
| 84.183872647 907.0 |
| 84.197002930 875.0 |
| 84.210133212 796.0 |
| 84.223263495 839.0 |
| 84.236393778 855.0 |
| 84.249524060 838.0 |
| 84.262654343 882.0 |
| 84.275784626 877.0 |
| 84.288914908 885.0 |
| 84.302045191 884.0 |
| 84.315175474 835.0 |
| 84.328305756 860.0 |
| 84.341436039 841.0 |
| 84.354566322 876.0 |
| 84.367696604 834.0 |
| 84.380826887 846.0 |
| 84.393957170 900.0 |
| 84.407087452 848.0 |
| 84.420217735 845.0 |
| 84.433348018 847.0 |
| 84.446478300 833.0 |
| 84.459608583 869.0 |
| 84.472738866 920.0 |
| 84.485869148 793.0 |
| 84.498999431 799.0 |
| 84.512129714 821.0 |
| 84.525259997 881.0 |
| 84.538390279 862.0 |
| 84.551520562 835.0 |
| 84.564650845 867.0 |
| 84.577781127 870.0 |
| 84.590911410 846.0 |
| 84.604041693 823.0 |
| 84.617171975 875.0 |
| 84.630302258 882.0 |
| 84.643432541 844.0 |
| 84.656562823 822.0 |
| 84.669693106 821.0 |
| 84.682823389 862.0 |
| 84.695953671 870.0 |
| 84.709083954 847.0 |
| 84.722214237 866.0 |
| 84.735344519 866.0 |
| 84.748474802 851.0 |
| 84.761605085 855.0 |
| 84.774735367 836.0 |
| 84.787865650 845.0 |
| 84.800995933 824.0 |
| 84.814126215 832.0 |
| 84.827256498 882.0 |
| 84.840386781 886.0 |
| 84.853517063 817.0 |
| 84.866647346 797.0 |
| 84.879777629 860.0 |
| 84.892907911 827.0 |
| 84.906038194 858.0 |
| 84.919168477 832.0 |
| 84.932298759 832.0 |
| 84.945429042 866.0 |
| 84.958559325 838.0 |
| 84.971689608 860.0 |
| 84.984819890 866.0 |
| 84.997950173 882.0 |
| 85.011080456 864.0 |
| 85.024210738 819.0 |
| 85.037341021 867.0 |
| 85.050471304 848.0 |
| 85.063601586 882.0 |
| 85.076731869 876.0 |
| 85.089862152 819.0 |
| 85.102992434 881.0 |
| 85.116122717 847.0 |
| 85.129253000 817.0 |
| 85.142383282 858.0 |
| 85.155513565 842.0 |
| 85.168643848 841.0 |
| 85.181774130 851.0 |
| 85.194904413 894.0 |
| 85.208034696 844.0 |
| 85.221164978 867.0 |
| 85.234295261 847.0 |
| 85.247425544 809.0 |
| 85.260555826 864.0 |
| 85.273686109 801.0 |
| 85.286816392 835.0 |
| 85.299946674 853.0 |
| 85.313076957 855.0 |
| 85.326207240 821.0 |
| 85.339337522 870.0 |
| 85.352467805 860.0 |
| 85.365598088 847.0 |
| 85.378728371 799.0 |
| 85.391858653 858.0 |
| 85.404988936 813.0 |
| 85.418119219 781.0 |
| 85.431249501 864.0 |
| 85.444379784 880.0 |
| 85.457510067 749.0 |
| 85.470640349 867.0 |
| 85.483770632 908.0 |
| 85.496900915 824.0 |
| 85.510031197 864.0 |
| 85.523161480 824.0 |
| 85.536291763 812.0 |
| 85.549422045 844.0 |
| 85.562552328 859.0 |
| 85.575682611 808.0 |
| 85.588812893 835.0 |
| 85.601943176 815.0 |
| 85.615073459 851.0 |
| 85.628203741 814.0 |
| 85.641334024 816.0 |
| 85.654464307 862.0 |
| 85.667594589 806.0 |
| 85.680724872 818.0 |
| 85.693855155 848.0 |
| 85.706985437 844.0 |
| 85.720115720 833.0 |
| 85.733246003 789.0 |
| 85.746376285 782.0 |
| 85.759506568 763.0 |
| 85.772636851 872.0 |
| 85.785767133 855.0 |
| 85.798897416 838.0 |
| 85.812027699 851.0 |
| 85.825157982 840.0 |
| 85.838288264 877.0 |
| 85.851418547 904.0 |
| 85.864548830 825.0 |
| 85.877679112 844.0 |
| 85.890809395 808.0 |
| 85.903939678 813.0 |
| 85.917069960 837.0 |
| 85.930200243 886.0 |
| 85.943330526 784.0 |
| 85.956460808 861.0 |
| 85.969591091 754.0 |
| 85.982721374 790.0 |
| 85.995851656 832.0 |
| 86.008981939 821.0 |
| 86.022112222 865.0 |
| 86.035242504 842.0 |
| 86.048372787 777.0 |
| 86.061503070 799.0 |
| 86.074633352 760.0 |
| 86.087763635 784.0 |
| 86.100893918 798.0 |
| 86.114024200 733.0 |
| 86.127154483 783.0 |
| 86.140284766 818.0 |
| 86.153415048 807.0 |
| 86.166545331 761.0 |
| 86.179675614 853.0 |
| 86.192805896 795.0 |
| 86.205936179 778.0 |
| 86.219066462 793.0 |
| 86.232196744 847.0 |
| 86.245327027 772.0 |
| 86.258457310 864.0 |
| 86.271587593 797.0 |
| 86.284717875 870.0 |
| 86.297848158 853.0 |
| 86.310978441 800.0 |
| 86.324108723 782.0 |
| 86.337239006 795.0 |
| 86.350369289 818.0 |
| 86.363499571 895.0 |
| 86.376629854 804.0 |
| 86.389760137 822.0 |
| 86.402890419 808.0 |
| 86.416020702 774.0 |
| 86.429150985 814.0 |
| 86.442281267 859.0 |
| 86.455411550 888.0 |
| 86.468541833 901.0 |
| 86.481672115 749.0 |
| 86.494802398 843.0 |
| 86.507932681 722.0 |
| 86.521062963 817.0 |
| 86.534193246 793.0 |
| 86.547323529 805.0 |
| 86.560453811 784.0 |
| 86.573584094 839.0 |
| 86.586714377 770.0 |
| 86.599844659 789.0 |
| 86.612974942 763.0 |
| 86.626105225 809.0 |
| 86.639235507 807.0 |
| 86.652365790 761.0 |
| 86.665496073 774.0 |
| 86.678626355 737.0 |
| 86.691756638 793.0 |
| 86.704886921 767.0 |
| 86.718017204 847.0 |
| 86.731147486 800.0 |
| 86.744277769 795.0 |
| 86.757408052 827.0 |
| 86.770538334 793.0 |
| 86.783668617 761.0 |
| 86.796798900 782.0 |
| 86.809929182 790.0 |
| 86.823059465 762.0 |
| 86.836189748 835.0 |
| 86.849320030 786.0 |
| 86.862450313 756.0 |
| 86.875580596 767.0 |
| 86.888710878 804.0 |
| 86.901841161 742.0 |
| 86.914971444 753.0 |
| 86.928101726 826.0 |
| 86.941232009 862.0 |
| 86.954362292 815.0 |
| 86.967492574 781.0 |
| 86.980622857 822.0 |
| 86.993753140 843.0 |
| 87.006883422 769.0 |
| 87.020013705 762.0 |
| 87.033143988 810.0 |
| 87.046274270 781.0 |
| 87.059404553 819.0 |
| 87.072534836 759.0 |
| 87.085665118 782.0 |
| 87.098795401 820.0 |
| 87.111925684 803.0 |
| 87.125055966 761.0 |
| 87.138186249 825.0 |
| 87.151316532 797.0 |
| 87.164446815 821.0 |
| 87.177577097 801.0 |
| 87.190707380 796.0 |
| 87.203837663 739.0 |
| 87.216967945 790.0 |
| 87.230098228 788.0 |
| 87.243228511 799.0 |
| 87.256358793 844.0 |
| 87.269489076 770.0 |
| 87.282619359 782.0 |
| 87.295749641 776.0 |
| 87.308879924 757.0 |
| 87.322010207 781.0 |
| 87.335140489 783.0 |
| 87.348270772 790.0 |
| 87.361401055 734.0 |
| 87.374531337 785.0 |
| 87.387661620 759.0 |
| 87.400791903 763.0 |
| 87.413922185 807.0 |
| 87.427052468 732.0 |
| 87.440182751 803.0 |
| 87.453313033 799.0 |
| 87.466443316 820.0 |
| 87.479573599 783.0 |
| 87.492703881 798.0 |
| 87.505834164 818.0 |
| 87.518964447 740.0 |
| 87.532094729 756.0 |
| 87.545225012 829.0 |
| 87.558355295 766.0 |
| 87.571485578 794.0 |
| 87.584615860 768.0 |
| 87.597746143 786.0 |
| 87.610876426 828.0 |
| 87.624006708 756.0 |
| 87.637136991 748.0 |
| 87.650267274 741.0 |
| 87.663397556 818.0 |
| 87.676527839 789.0 |
| 87.689658122 765.0 |
| 87.702788404 735.0 |
| 87.715918687 810.0 |
| 87.729048970 756.0 |
| 87.742179252 790.0 |
| 87.755309535 756.0 |
| 87.768439818 780.0 |
| 87.781570100 738.0 |
| 87.794700383 825.0 |
| 87.807830666 815.0 |
| 87.820960948 806.0 |
| 87.834091231 760.0 |
| 87.847221514 795.0 |
| 87.860351796 782.0 |
| 87.873482079 815.0 |
| 87.886612362 774.0 |
| 87.899742644 729.0 |
| 87.912872927 781.0 |
| 87.926003210 770.0 |
| 87.939133492 788.0 |
| 87.952263775 806.0 |
| 87.965394058 745.0 |
| 87.978524340 764.0 |
| 87.991654623 796.0 |
| 88.004784906 759.0 |
| 88.017915189 733.0 |
| 88.031045471 768.0 |
| 88.044175754 739.0 |
| 88.057306037 761.0 |
| 88.070436319 777.0 |
| 88.083566602 770.0 |
| 88.096696885 717.0 |
| 88.109827167 744.0 |
| 88.122957450 752.0 |
| 88.136087733 748.0 |
| 88.149218015 791.0 |
| 88.162348298 746.0 |
| 88.175478581 759.0 |
| 88.188608863 731.0 |
| 88.201739146 783.0 |
| 88.214869429 794.0 |
| 88.227999711 753.0 |
| 88.241129994 746.0 |
| 88.254260277 784.0 |
| 88.267390559 737.0 |
| 88.280520842 801.0 |
| 88.293651125 854.0 |
| 88.306781407 757.0 |
| 88.319911690 747.0 |
| 88.333041973 786.0 |
| 88.346172255 771.0 |
| 88.359302538 783.0 |
| 88.372432821 749.0 |
| 88.385563103 755.0 |
| 88.398693386 756.0 |
| 88.411823669 781.0 |
| 88.424953951 760.0 |
| 88.438084234 796.0 |
| 88.451214517 794.0 |
| 88.464344800 735.0 |
| 88.477475082 791.0 |
| 88.490605365 739.0 |
| 88.503735648 739.0 |
| 88.516865930 769.0 |
| 88.529996213 790.0 |
| 88.543126496 785.0 |
| 88.556256778 735.0 |
| 88.569387061 787.0 |
| 88.582517344 781.0 |
| 88.595647626 785.0 |
| 88.608777909 775.0 |
| 88.621908192 744.0 |
| 88.635038474 776.0 |
| 88.648168757 769.0 |
| 88.661299040 761.0 |
| 88.674429322 762.0 |
| 88.687559605 826.0 |
| 88.700689888 758.0 |
| 88.713820170 796.0 |
| 88.726950453 789.0 |
| 88.740080736 805.0 |
| 88.753211018 762.0 |
| 88.766341301 776.0 |
| 88.779471584 707.0 |
| 88.792601866 711.0 |
| 88.805732149 719.0 |
| 88.818862432 732.0 |
| 88.831992714 759.0 |
| 88.845122997 770.0 |
| 88.858253280 828.0 |
| 88.871383562 802.0 |
| 88.884513845 751.0 |
| 88.897644128 729.0 |
| 88.910774411 748.0 |
| 88.923904693 736.0 |
| 88.937034976 798.0 |
| 88.950165259 797.0 |
| 88.963295541 790.0 |
| 88.976425824 759.0 |
| 88.989556107 793.0 |
| 89.002686389 739.0 |
| 89.015816672 707.0 |
| 89.028946955 738.0 |
| 89.042077237 752.0 |
| 89.055207520 709.0 |
| 89.068337803 704.0 |
| 89.081468085 766.0 |
| 89.094598368 755.0 |
| 89.107728651 782.0 |
| 89.120858933 773.0 |
| 89.133989216 754.0 |
| 89.147119499 777.0 |
| 89.160249781 732.0 |
| 89.173380064 757.0 |
| 89.186510347 703.0 |
| 89.199640629 744.0 |
| 89.212770912 727.0 |
| 89.225901195 771.0 |
| 89.239031477 779.0 |
| 89.252161760 741.0 |
| 89.265292043 740.0 |
| 89.278422325 760.0 |
| 89.291552608 747.0 |
| 89.304682891 822.0 |
| 89.317813173 814.0 |
| 89.330943456 735.0 |
| 89.344073739 778.0 |
| 89.357204022 748.0 |
| 89.370334304 719.0 |
| 89.383464587 718.0 |
| 89.396594870 757.0 |
| 89.409725152 791.0 |
| 89.422855435 776.0 |
| 89.435985718 732.0 |
| 89.449116000 803.0 |
| 89.462246283 796.0 |
| 89.475376566 731.0 |
| 89.488506848 792.0 |
| 89.501637131 780.0 |
| 89.514767414 718.0 |
| 89.527897696 698.0 |
| 89.541027979 691.0 |
| 89.554158262 752.0 |
| 89.567288544 720.0 |
| 89.580418827 744.0 |
| 89.593549110 828.0 |
| 89.606679392 785.0 |
| 89.619809675 726.0 |
| 89.632939958 747.0 |
| 89.646070240 740.0 |
| 89.659200523 699.0 |
| 89.672330806 742.0 |
| 89.685461088 769.0 |
| 89.698591371 792.0 |
| 89.711721654 773.0 |
| 89.724851936 703.0 |
| 89.737982219 691.0 |
| 89.751112502 758.0 |
| 89.764242784 741.0 |
| 89.777373067 705.0 |
| 89.790503350 740.0 |
| 89.803633633 757.0 |
| 89.816763915 713.0 |
| 89.829894198 733.0 |
| 89.843024481 774.0 |
| 89.856154763 757.0 |
| 89.869285046 725.0 |
| 89.882415329 729.0 |
| 89.895545611 787.0 |
| 89.908675894 772.0 |
| 89.921806177 792.0 |
| 89.934936459 750.0 |
| 89.948066742 750.0 |
| 89.961197025 781.0 |
| 89.974327307 808.0 |
| 89.987457590 802.0 |

**^1^H NMR Spectra for the Primary Amine Products**


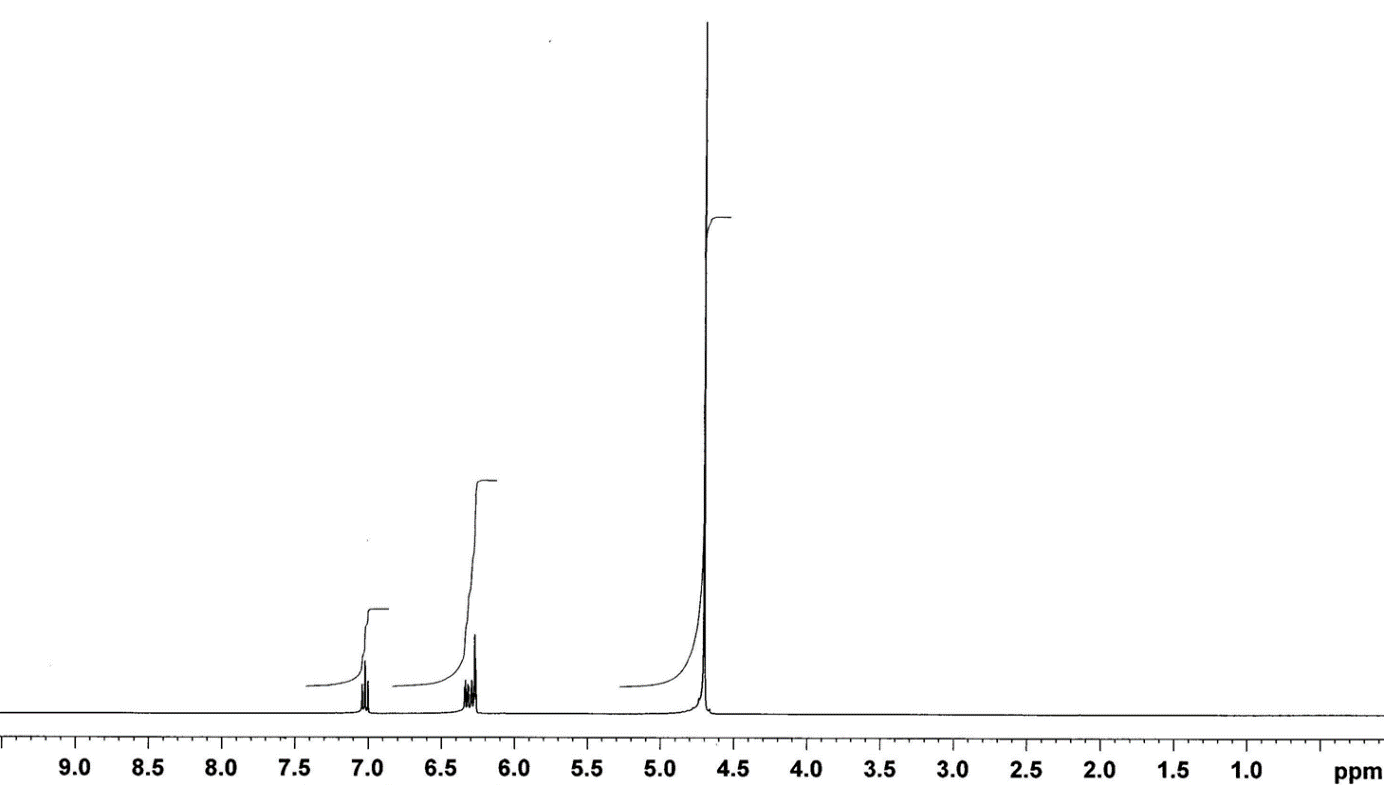


**3-Aminophenol.** ^1^H NMR (400 MHz, D_2_O): *δ* 7.04 (m, 1H), 6.31 (m, 3H).


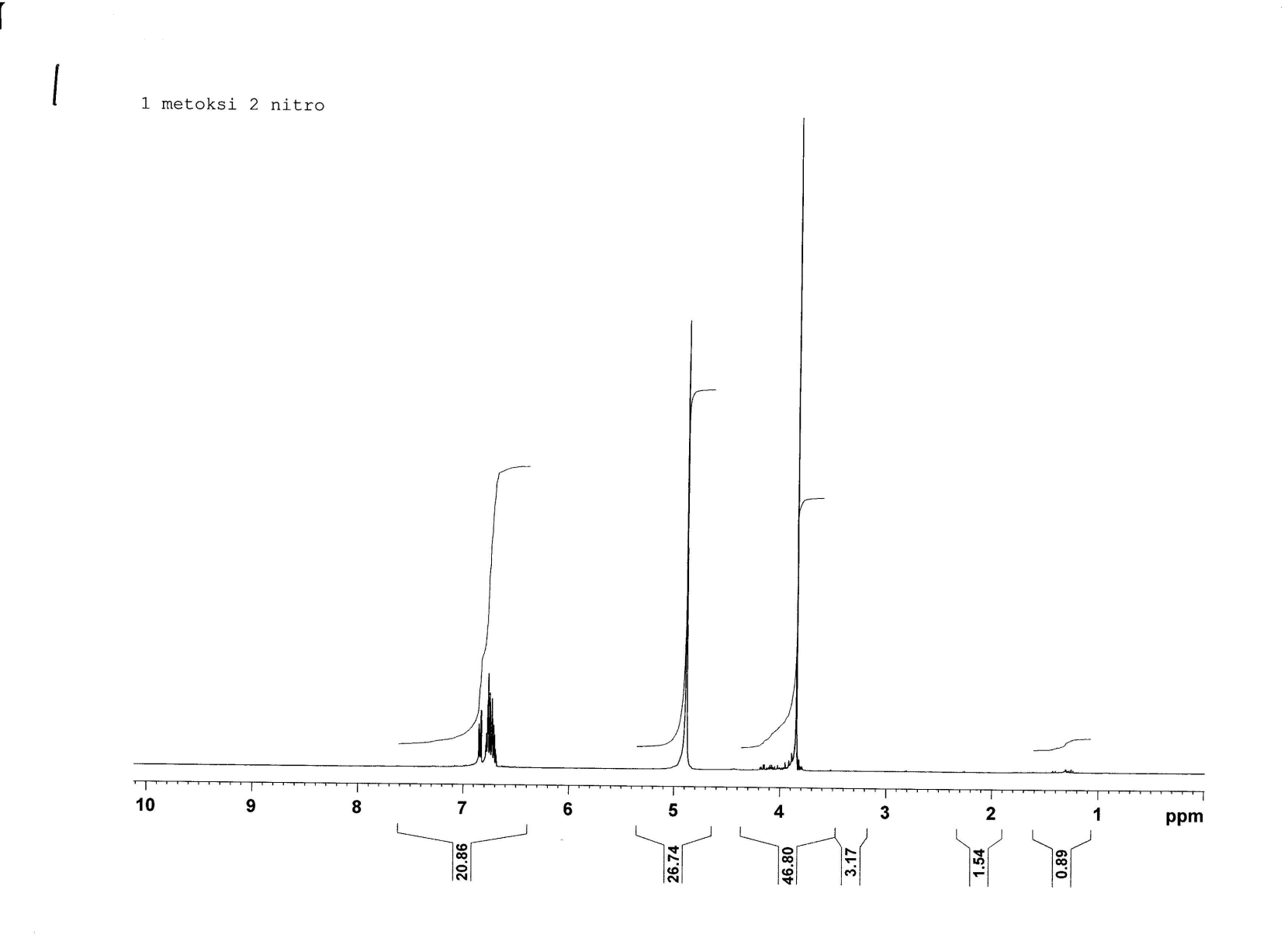


**2-Methoxy aniline.** ^1^H NMR (400 MHz, D_2_O) *δ* 6.85-6.77 (m, 2H), 6.77-6.68 (m, 2H), 3.85 (s, 3H).


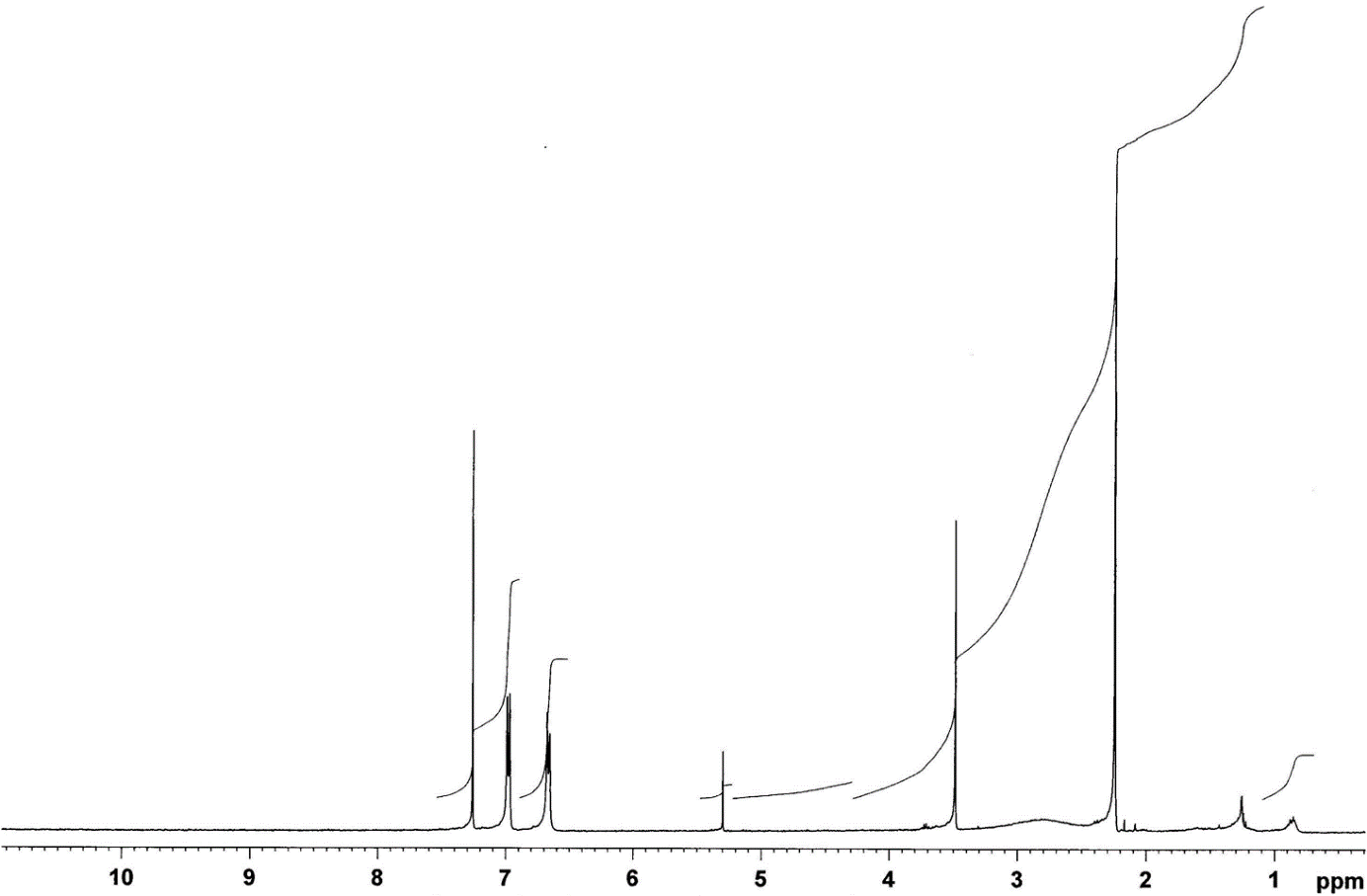


**4-Aminotoluene.** ^1^H NMR (400 MHz, CDCl_3_): *δ* 7.00 (t, *J* = 8.0 Hz, 2H), 6.63 (t, *J* = 6.4 Hz, 2H), 2.26 (s, 3H).


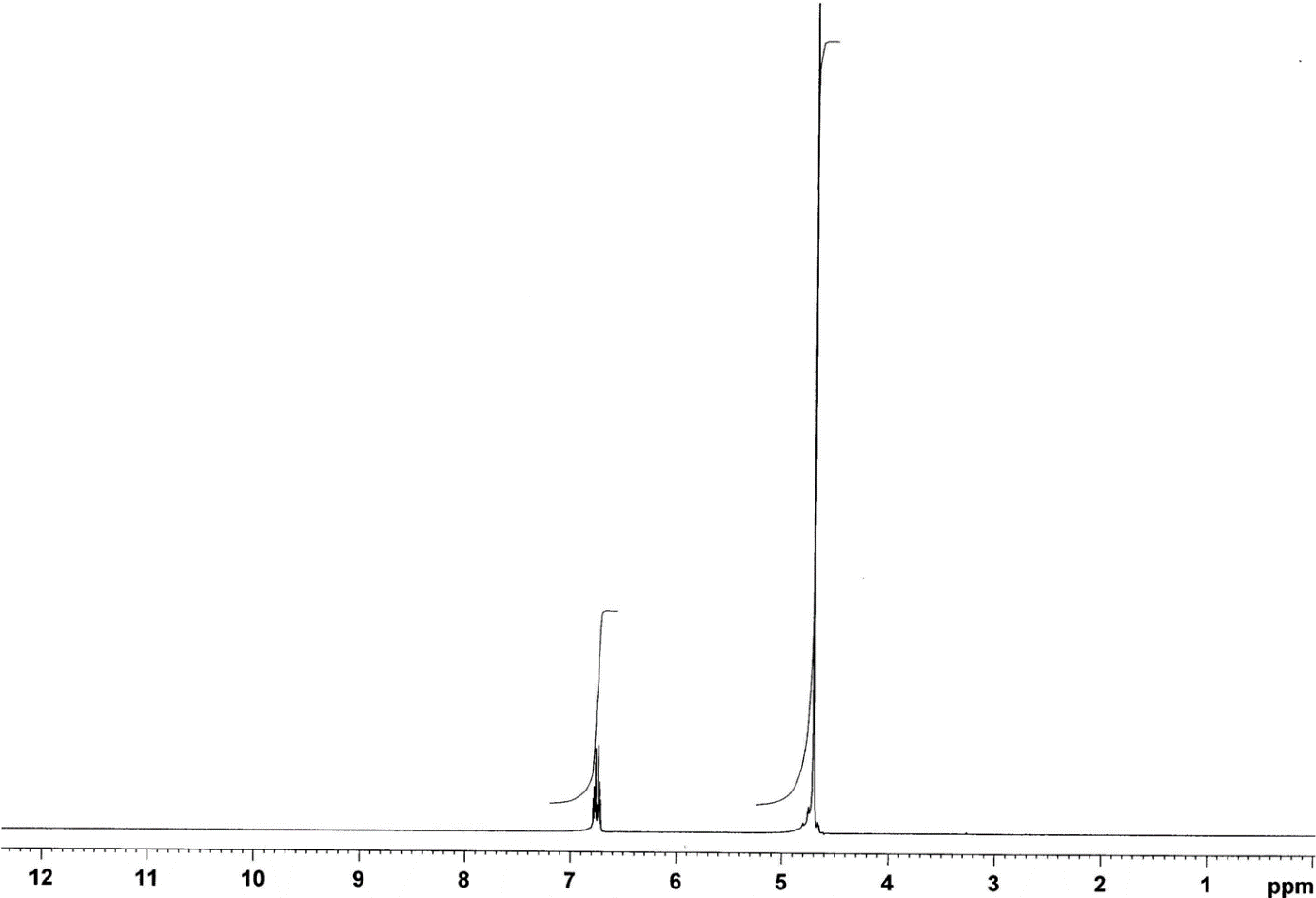


**1,2-Diaminobenzene.** ^1^H NMR (400 MHz, D_2_O): *δ* 6.71-6.69 (m, 4H).


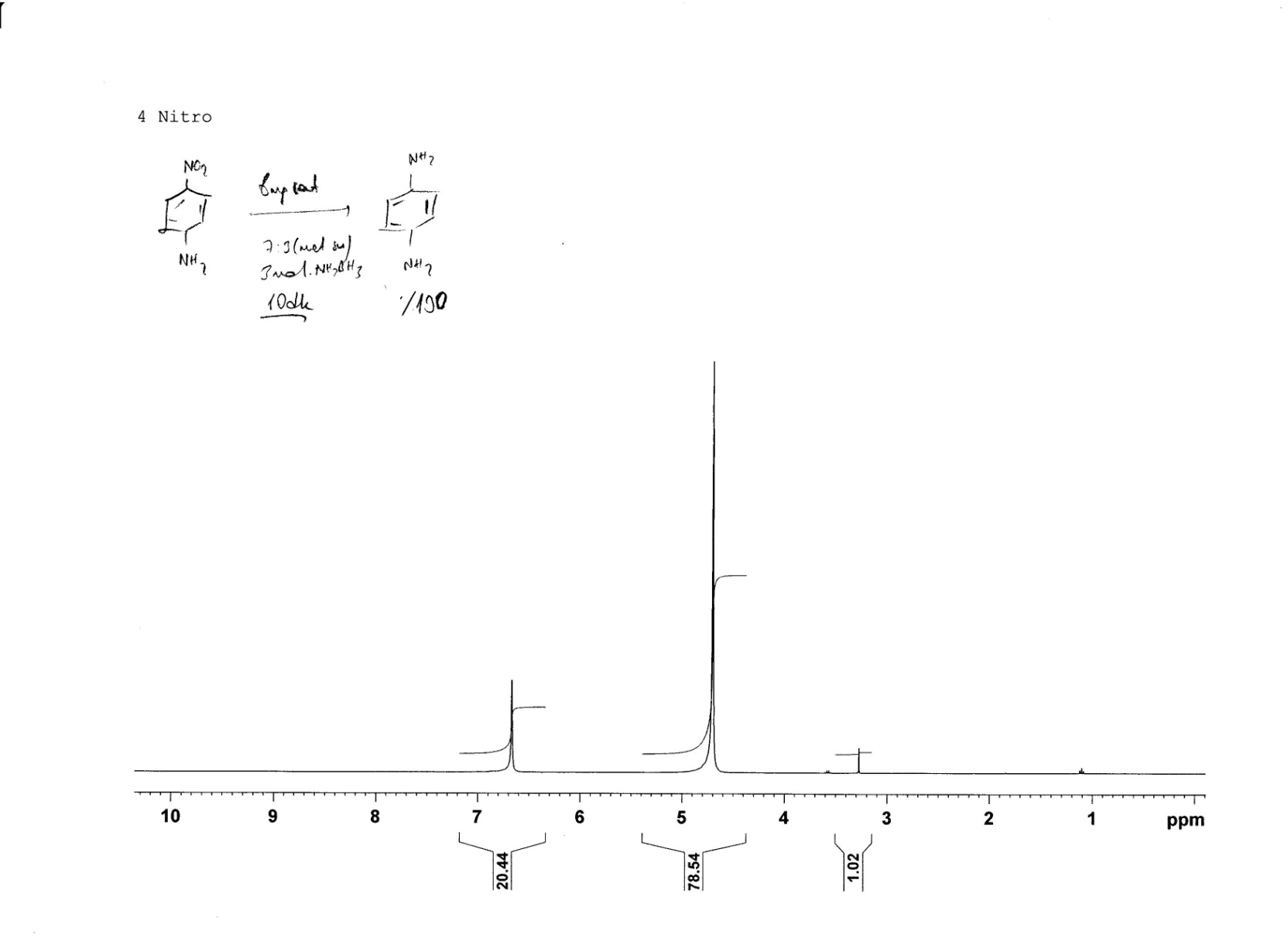


**1,4-Diaminobenzene**. ^1^H NMR (400 MHz, D_2_O): *δ* 6.68 (s, 4H).

**2,6-dimethylaniline.** ^1^H NMR (400MHz, CDCl_3_): *δ* 6.96 (d, *J* = 7.4 Hz, 2H), 6.65 (t, *J* = 7.4 Hz, 1H), 2.19 (s, 6H).

**2-propylaniline.** ^1^H NMR (400MHz, CDCl_3_): *δ* 7.14-6.96 (m, 2H), 6.77 (td, *J* = 7.3, 5.0 Hz, 1H), 6.70 (d, *J* = 7.8 Hz, 1H), 2.62-2.38 (m, 2H), 1.78-1.55 (m, 2H), 1.12-0.91 (m, 3H)

**4-bromoaniline.** ^1^H NMR (400MHz, CDCl_3_): *δ* 7.24-7.19 (m, 2H), 6.58-6.52 (m, 2H).


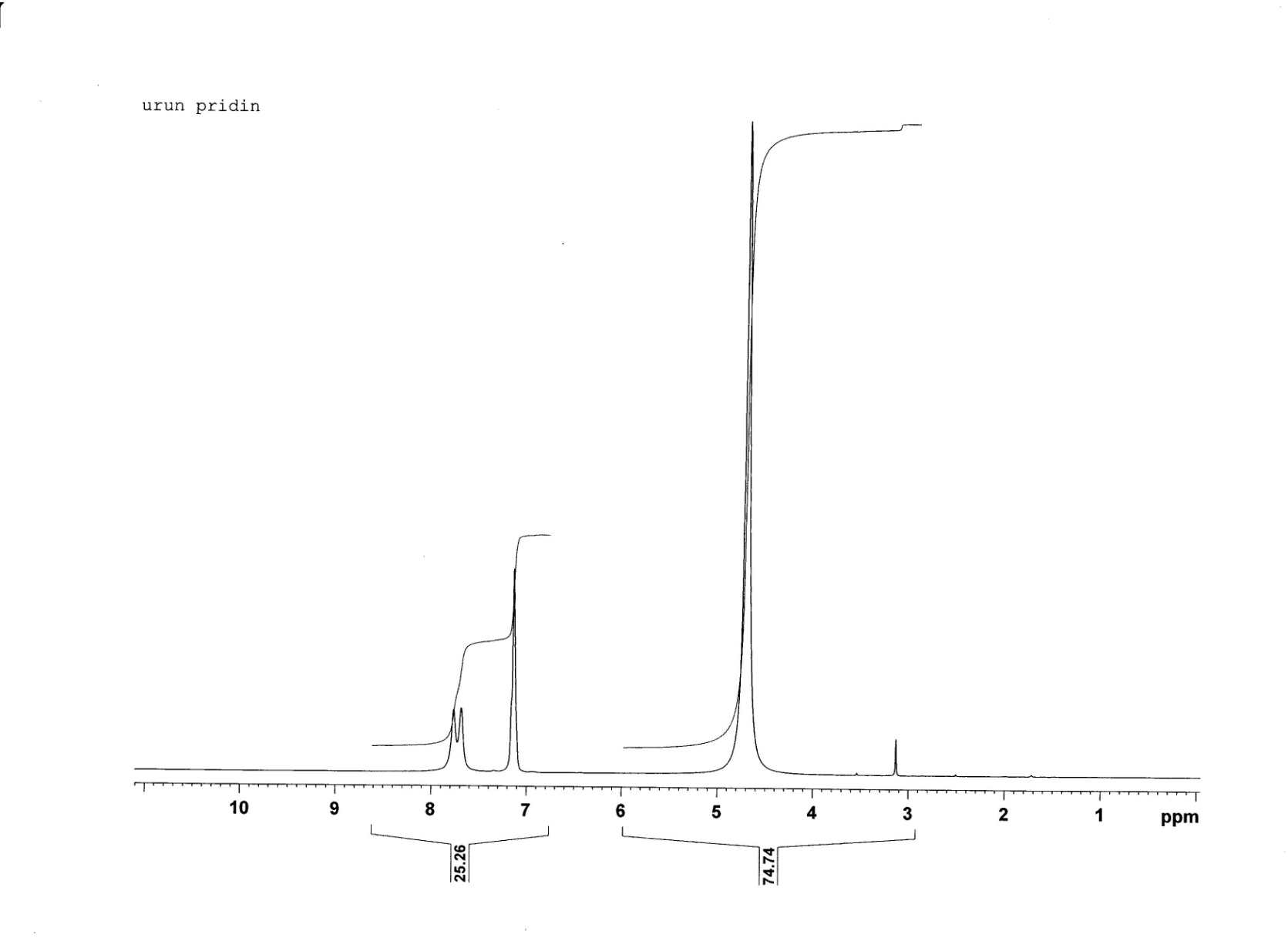


**2-Chloro-5-aminopyridine.** ^1^H NMR (400 MHz, D_2_O): *δ* 7.73 (m, 1H), 7.15 (m, 2H).


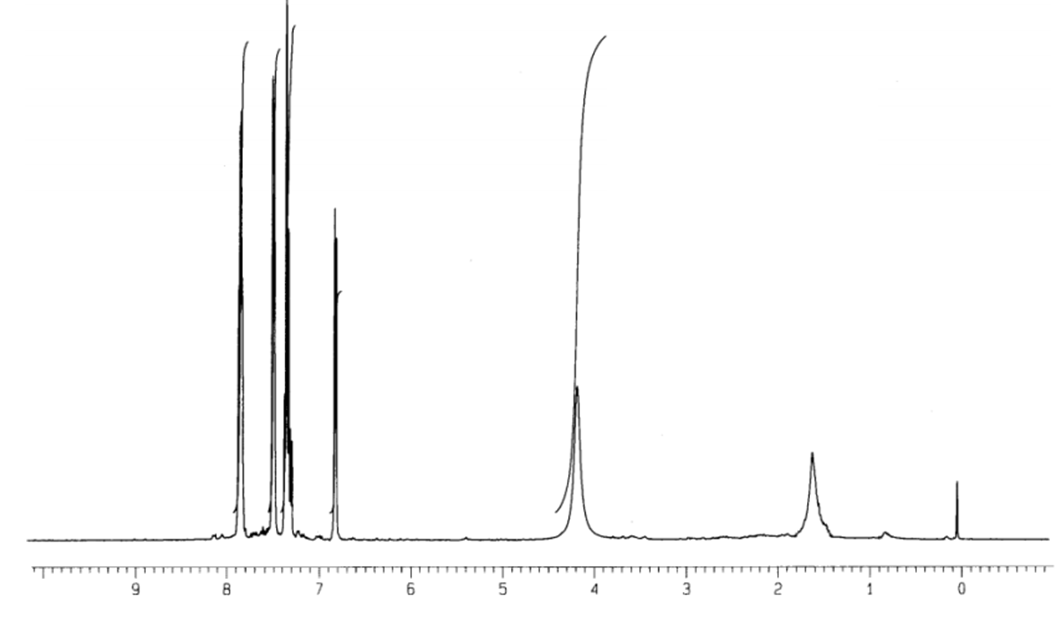


**Naphthalen-1-amine.** ^1^H NMR (400MHz, CDCl_3_): *δ* 7.80 (dd, *J* = 8.8, 5.6 Hz, 2H), 7.45 (d, *J* = 8.0 Hz, 2H), 7.30 (t, *J* = 8.8 Hz, 2H), 6.77 (d, *J* = 6.8 Hz, 1H), 4.13 (s, 2H).


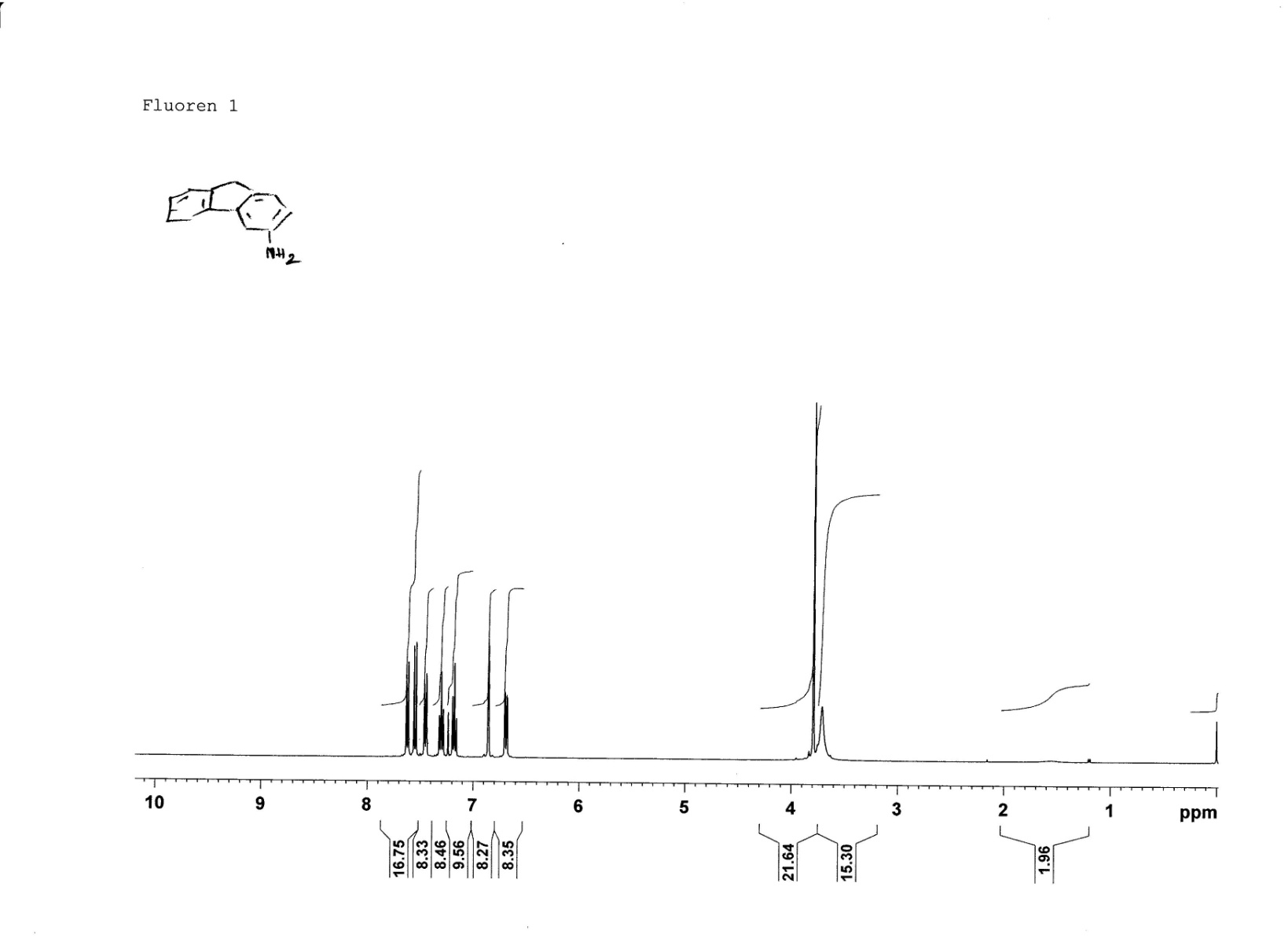


**2-Aminofluorene.** ^1^H NMR (400 MHz, CDCl_3_): *δ* 7.64 (d, *J* = 7.5 Hz, 1H), 7.57 (d, *J* = 8.1 Hz, 1H), 7.47 (d, *J* = 7.5 Hz, 1H), 7.32 (t, *J* = 7.5 Hz, 1H), 7.21 (t, *J* = 7.5 Hz, 1H), 6.89 (s, 1H), 6.69 (dd, *J* = 8.1, 2.2 Hz, 1H), 3.71 (s, 2H).

**2,3-dihydrobenzo[b][1,4]dioxin-6-amine.** ^1^H NMR (400MHz, CDCl_3_): *δ* 6.65 (t, *J* = 6.6 Hz, 1H), 6.23 (dd, *J* = 4.1, 1.5 Hz, 1H), 6.21-6.15 (m, 1H), 4.24-1.10 (m, 4H), 3.40 (bs, 2H).

**2-amino-9H-fluoren-9-ol.** ^1^H NMR (400MHz, CDCl_3_): *δ* 7.54 (d, *J* = 7.3 Hz, 1H), 7.42-7.36 (m, 1H), 7.35-7.30 (m, 1H), 7.25 (s, 1H), 7.13 (t, *J* = 7.4 Hz, 1H), 6.95 (t, *J* = 4.0 Hz, 1H), 6.70 (dt, *J* = 12.0, 6.0 Hz, 1H).

**2-Aminobenzonitrile .** ^1^H NMR (400MHz, CD_3_OD): *δ* 7.60–7.39 (m, 1H), 7.16 (ddd, *J* = 7.2, 1.3, 0.7 Hz, 1H), 6.72 (t, *J* = 11.6 Hz, 1H), 6.64-6.47 (m, 1H).


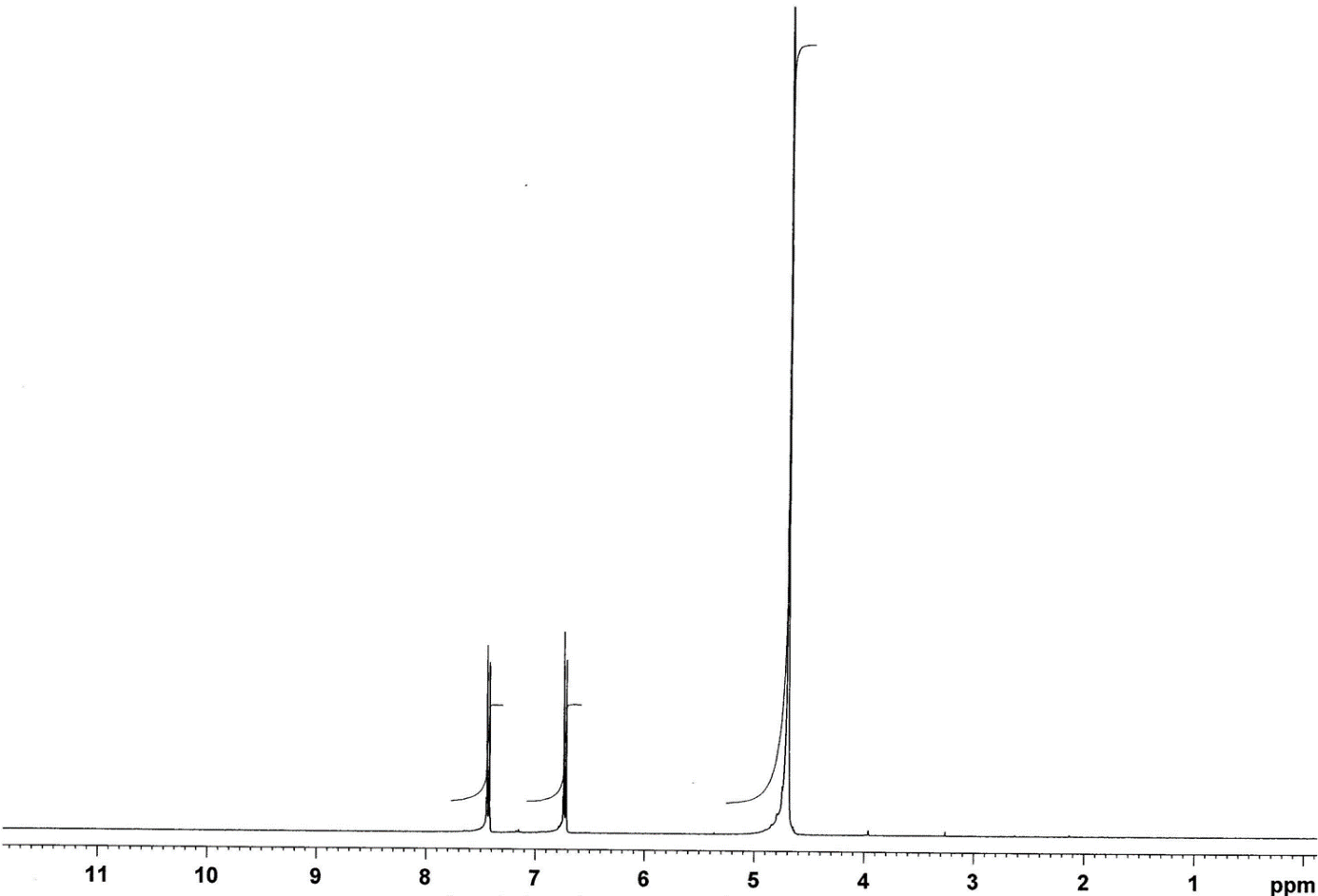


**4-Aminobenzonitrile.** ^1^H NMR (400 MHz, D_2_O): *δ* 7.42 (d, *J* = 8.6 Hz, 2H), 6.71 (d, *J* = 8.6 Hz, 2H).
